# Obesity-Related Metabolome and Gut Microbiota Profiles of Juvenile Göttingen Minipigs—Long-Term Intake of Fructose and Resistant Starch

**DOI:** 10.3390/metabo10110456

**Published:** 2020-11-12

**Authors:** Mihai V. Curtasu, Valeria Tafintseva, Zachary A. Bendiks, Maria L. Marco, Achim Kohler, Yetong Xu, Natalja P. Nørskov, Helle Nygaard Lærke, Knud Erik Bach Knudsen, Mette Skou Hedemann

**Affiliations:** 1 Department of Animal Science, Aarhus University, Blichers Alle 20, DK-8830 Tjele, Denmark; yetongxu@anis.au.dk (Y.X.); natalja.norskov@anis.au.dk (N.P.N.); hellen.laerke@anis.au.dk (H.N.L.); knuderik.bachknudsen@anis.au.dk (K.E.B.K.); mette.hedemann@anis.au.dk (M.S.H.); 2 Faculty of Science and Technology (RealTek), Norwegian University of Life Sciences, Drøbakveien 31, 1430 Ås, Norway; valeria.tafintseva@nmbu.no (V.T.); achim.kohler@nmbu.no (A.K.); 3 Department of Food Science and Technology, University of California, Davis, CA 95616, USA; zabendiks@ucdavis.edu (Z.A.B.); mmarco@ucdavis.edu (M.L.M.)

**Keywords:** miniature pigs, nutrition, metabolic syndrome, multi-block analysis, obesity

## Abstract

The metabolome and gut microbiota were investigated in a juvenile Göttingen minipig model. This study aimed to explore the metabolic effects of two carbohydrate sources with different degrees of risk in obesity development when associated with a high fat intake. A high-risk (HR) high-fat diet containing 20% fructose was compared to a control lower-risk (LR) high-fat diet where a similar amount of carbohydrate was provided as a mix of digestible and resistant starch from high amylose maize. Both diets were fed *ad libitum*. Non-targeted metabolomics was used to explore plasma, urine, and feces samples over five months. Plasma and fecal short-chain fatty acids were targeted and quantified. Fecal microbiota was analyzed using genomic sequencing. Data analysis was performed using sparse multi-block partial least squares regression. The LR diet increased concentrations of fecal and plasma total short-chain fatty acids, primarily acetate, and there was a higher relative abundance of microbiota associated with acetate production such as *Bacteroidetes* and *Ruminococcus*. A higher proportion of *Firmicutes* was measured with the HR diet, together with a lower alpha diversity compared to the LR diet. Irrespective of diet, the *ad libitum* exposure to the high-energy diets was accompanied by well-known biomarkers associated with obesity and diabetes, particularly branched-chain amino acids, keto acids, and other catabolism metabolites.

## 1. Introduction

The abnormal accumulation of fat in adipose tissues, i.e., obesity, is a major component in the development of metabolic syndrome (MetS) [1]. Obesity and MetS affect the human population through a series of co-morbidities, primarily cardiovascular disease (CVD), diabetes, and hypertension, and are nowadays considered as worldwide epidemics [2]. Fructose has been suggested as a contributing risk factor to the development of obesity and MetS [3,4]. The modern human population has been increasingly exposed to fructose, through the addition of high-fructose corn syrup to pre-processed foods and soft drinks [3,5]. Compared to glucose, fructose only modestly stimulates insulin secretion and insulin is not necessary for the cellular uptake of this carbohydrate. Once available for hepatic metabolism, fructose is rapidly converted to fructose-1-phosphate, bypassing regulating mechanisms that are found in glycolysis [6]. Furthermore, in the context of excessive intake, fructose has a potential lipogenic effect through precursors that can be used for de novo lipogenesis (DNL), leading to fat accumulation and hepatic steatosis [6,7]. Recently, young Göttingen Minipigs fed *ad libitum* fructose-rich diets developed fewer signs of metabolic syndrome but presented mild hepatic tissue inflammation without dyslipidemia when compared to a control diet based on high amylose maize starch (HiMaize) [8].

As a tool for mitigating obesity and MetS development, food sources rich in resistant starch (RS) such as HiMaize have been widely investigated in nutritional studies for their beneficial effects [9,10,11]. RS escapes enzymatic digestion in the stomach and small intestine and provides a substrate for microbial growth in the gut [11]. Replacing energy-dense carbohydrates and thus decreasing the overall energy density of diets is one beneficial physiological effect of RS. Through gut metabolism, RS promotes the formation of short-chain fatty acids (SCFA) with a wide variety of positive effects on systemic parameters [12,13] and helps to maintain the integrity of the gut barrier [13]. SCFA also promotes the secretion of satiety gut hormones such as peptide tyrosine-tyrosine (PYY) and glucagon-like-peptide-1 (GLP-1) [14,15]. This leads to increased satiation and ultimately a decrease in energy intake. However, recent work has shown that although a HiMaize-rich diet increased PYY, the effect on satiety was minimal and the minipigs developed obesity at the same rate as minipigs fed a diet high in fructose [8].

Metabolomics is currently an essential tool for screening biomarkers and the exploration of underlying mechanisms of disease [16]. In dietary intervention studies, low molecular weight metabolites reflect changes in specific pathways linked to disease, nutrition, and even effects of the microbiota [17]. These approaches are complementary to taxonomic and phylogenetic investigations of bacteria by DNA sequencing. The intestinal microbiota can be affected by diet and these microorganisms are now understood to provide pivotal contributions to energy balance, metabolism, and body composition [18,19]. Previous studies in pigs and miniature pigs have revealed differential changes in the gut communities of obese animals compared to lean control groups [20,21]. However, less is known presently about the effects of a fructose-rich diet on microbiota ecology and the gut metabolome.

In order to further identify metabolic mechanisms associated with the intake of simple carbohydrates, samples from a Göttingen minipig obesity model [8] were analyzed using metabolomics and metataxonomics techniques. This would allow us to further explore the physiological effects of two specific dietary ingredients, which are considered to increase (fructose) or lower (HiMaize starch) the risk for developing metabolic abnormalities such as the progression of obesity and associated co-morbidities. The fecal microbiota was examined by 16S rRNA gene amplicon DNA sequencing and fecal SCFA by gas chromatography. Targeted liquid-chromatography mass spectrometry (LC-MS) was used to quantify plasma SCFA and a non-targeted LC-MS metabolomics approach was used to explore the metabolome of plasma, urine, and fecal samples. In the study, we aimed at examining the longitudinal changes in metabolome and gut microbiome over five months of *ad libitum* feed intake of a high-risk (HR) fructose-based diet compared to a lower-risk (LR) diet with a mix of digestible and resistant starch from HiMaize, with both diets being high in fat and presenting obesogenic qualities. Integrating the metabolomics and metataxonomics data in a multi-block analysis model was used to examine possible connections between the gut microbiome and fecal metabolome.

## 2. Results

### 2.1. Fecal Microbial Composition

**Figure 1:**
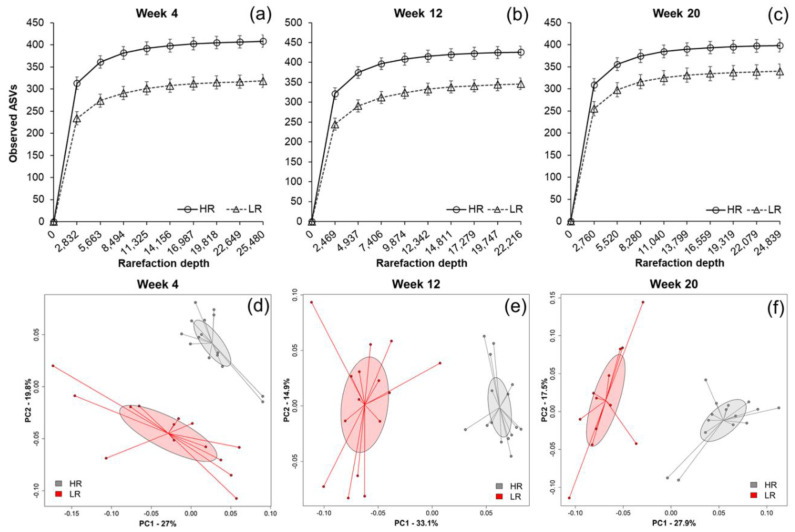
Alpha rarefaction curves (**a**–**c**) at each time collection point describing the number of observed amplicon sequence variants (ASVs) (*y*-axis) as a function of sequencing depth (*x*-axis); principal coordinates analysis (PCoA) plots of Weighted UniFrac metrics (**d**–**f**) representing individual fecal communities (●, high-risk (HR); ●, lower-risk (LR)) at each collection point. Confidence ellipses are presented in color for each dietary group (99% CI based on standard error).

The alpha-diversity of fecal microbiota collected from pigs fed the LR diet containing HiMaize was reduced compared to that of HR-fed pigs at each time point tested over the 20-week intervention (week 4, *p* < 0.001; week 12, *p* < 0.001; week 20, *p* = 0.01) (Figure 1a–c). Bacterial beta diversity also differed according to diet as shown by Principal Coordinates Analysis (PCoA) of Weighted UniFrac distance metric (Figure 1d–f). These differences were significant at all-time points according to permutational multivariate analysis of variance (PERMANOVA) (week 4, *p* = 0.001; week 12, *p* = 0.001; week 20, *p* = 0.001). Notably, there were no within-group differences in alpha diversity (HR, *p* = 0.423; LR, *p* = 0.155) nor beta diversity (HR, *p* = 0.137; LR, *p* = 0.102) between time points (Figure S1).

**Figure 2:**
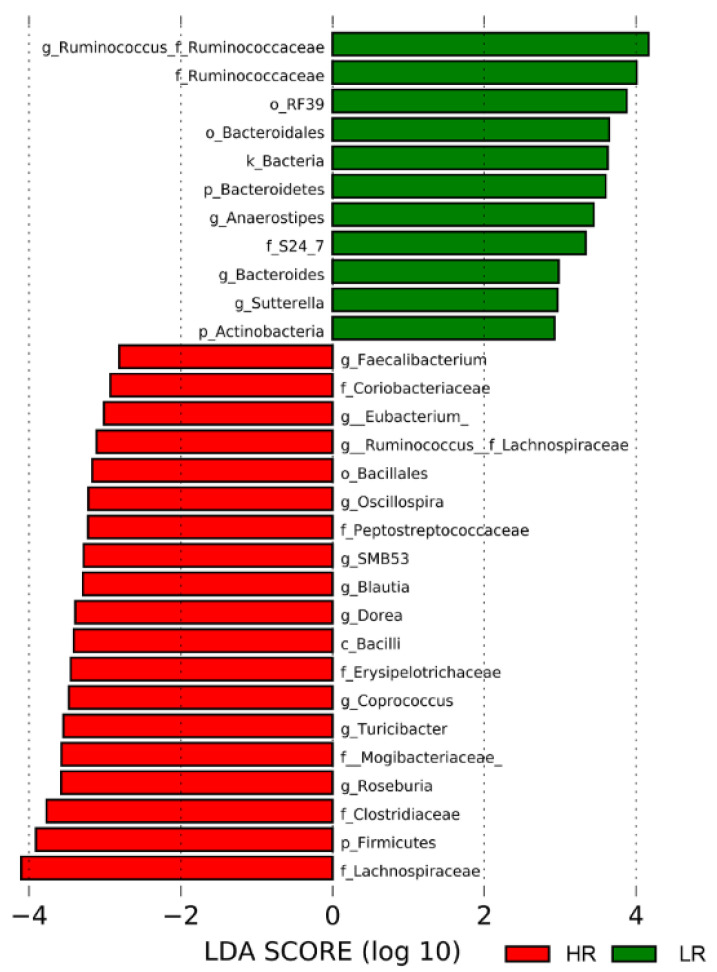
Linear discriminant analysis effect size (LEfSe) plot describing the enriched taxa identified in each treatment group across all time points (HR, n = 45; LR, n = 41). The plot displays taxa with linear discriminant analysis (LDA) scores above 2.0 and *p*-values < 0.01. Abbreviations: g, genus; f, family; o, order; k, kingdom; p, phylum; c, class.

The most abundant taxa detected in the fecal samples were *Firmicutes*, followed by *Bacteroidetes* and *Tenericutes*. At the taxonomic level, the microbiota of pigs fed the HR diet was enriched in bacteria from the *Lachnospiraceae* family, including members of the genera *Roseburia*, *Coprococcus*, *Dorea*, *Blautia*, and *Ruminococcus* (Figure 2). *Clostridiaceae*, *Erysipelotrichaceae*, *Peptostreptococcaceae*, *Mogibacteriaceae*, and *Coriobacteriaceae* families were also enriched with the HR diet (Figure 2). Consumption of the LR diet resulted in a higher relative abundance of *Ruminococcaceae*, particularly the *Ruminococcus* genus, as well as members of the uncultured RF39 order and the genus *Sutterella* (Figure 2). Further, we observed enrichment of the *Bacteroidetes* phylum, with species from the *Bacteroides* genus and members of the uncultured S24-7 family.

### 2.2. Short-Chain Fatty Acids

**Figure 3:**
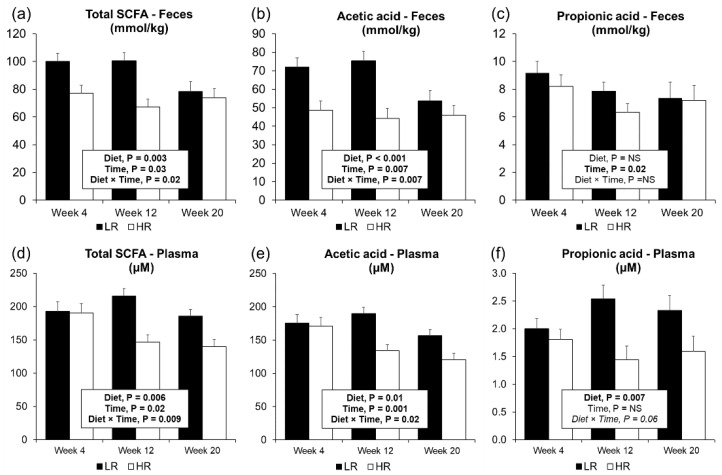
Short-chain fatty acids (SCFA) measured in feces and plasma from Gottingen Minipigs fed the HR or LR diets. Fecal SCFA were measured by gas chromatography (**a**–**c**). In fasting plasma SCFA were analyzed by targeted liquid chromatography mass spectrometry (**d**–**f**). Abbreviations: NA, non-significant (*p*-values > 0.1).

Short-chain fatty acids analyzed in feces and plasma can be found in detail in Tables S1 and S2, respectively. In feces, LR diet concentrations of total SCFA (Figure 3a), acetic acid (Figure 3b), total organic acids (Table S1), and total APB (acetic acid, propionic acid, butyric acid; Table S1) were higher when compared to the HR diet. Butyric acid as a proportion of total SCFA was measured higher with the HR diet compared to the LR diet and a tendency was observed for higher valeric acid attributed to the HR treatment (Table S1). Fecal SCFA decreased over time with lower concentrations from week 4 to week 20 of total SCFA (Figure 3a), acetic acid (Figure 3b), propionic acid (Figure 3c), APB, and acetic acid (%TSCFA) (Table S1). Plasma concentrations of total SCFA (Figure 3d), total organic acids, total branched-chain fatty acids (BCFA), and total APB were higher in the LR diet when compared to the HR diet (Table S2). Particularly higher levels of acetic acid (Figure 3e), propionic acid (Figure 3f), isovaleric acid, and isobutyric acid were measured in the plasma of LR diet pigs. Over time, a more pronounced decrease in plasma total SCFA (Figure 3d), acetic acid (Figure 3e), and total ABP (Table S2) was observed in the HR diet compared to the LR. Increasing concentrations of total BCFA and isobutyric acid were measured over time in plasma, at higher concentrations with the LR diet (Table S2).

### 2.3. Multi-Compartmental Non-Targeted Metabolomics—Diet Classification

**Figure 4:**
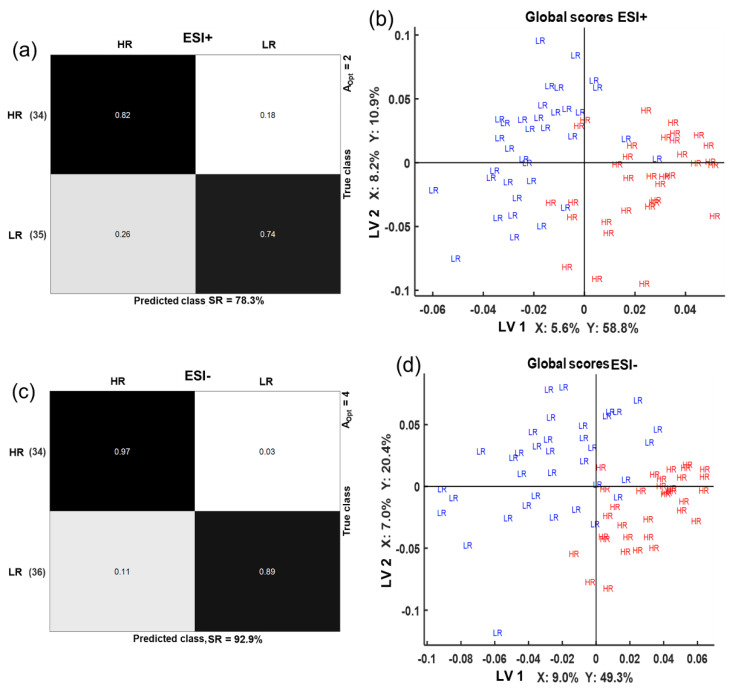
Sparse multi-block partial least squares regression (sMBPLSR) confusion matrices (**a**,**c**) and global score plots (**b**,**d**) from classification analysis of HR and LR groups using plasma, urine, and feces metabolomics in positive (**a**,**b**) and negative (**c**,**d**) ionization mode. The success rate (SR) of cross-validation is provided together with the number of latent variables of the models (A_Opt_). Numbers in parentheses (**a**,**c**) reflect the number of samples used in the multi-block analysis after a sample-to-sample correspondence. Explained variances (%) in matrices X and Y by the corresponding latent variables (LV) are provided (**b**,**d**).

Multivariate modeling was performed using metabolomics data in a multi-block setting by using sparse multi-block partial least square regression (sMBPLSR). In this study, the blocks of data are urine, plasma, and feces metabolomics data sets. Data from the positive electrospray ionization (ESI+) and negative electrospray ionization (ESI−) were analyzed separately, resulting in two dietary classification models. Models with a good classification success rate after cross-validation (SRcv) were obtained, SR_CV_ = 78% in ESI+ and SR_CV_ = 93% in ESI− (Figure 4a,c). The global score plot, obtained when all the blocks are combined, was also examined because it summarizes the sample pattern in a global space of the model. In this analysis, the global score plots show a good separation between the metabolic profiles of the LR and the HR groups along latent variable (LV) 1 for ESI+ (Figure 4b) and along the first two LVs for ESI− (Figure 4d). Block scores were also examined because they provide insight into the contribution of each blocks’ sample pattern into the global score sample pattern. Block scores and super weights of both sMBPLSR models are presented in Figures S2 and S3. We observed that the plasma block contributed most in differentiating the diets for the ESI+ model (Figure S2a) and the ESI− models (Figure S3a), whereas the feces block had a very high contribution for the diet separation in the ESI− model (Figure S3a). Urine had a very low contribution to the classification of diets in both ESI+ and ESI−.

**Figure 5:**
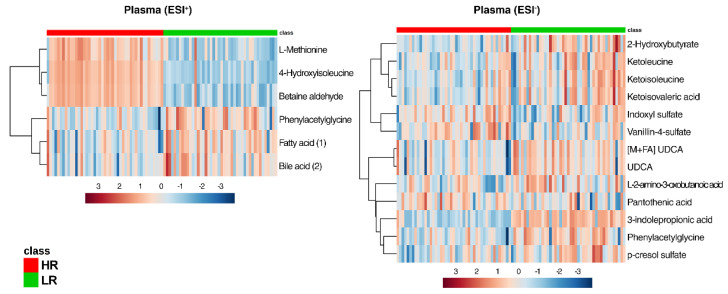
Heat maps of metabolites discriminating (*p* < 0.05) between the HR (red class) and the LR (green class) diet in plasma of Göttingen Minipigs in both positive and negative ionization mode (electrospray ionization (ESI+, ESI−)). Abbreviations: UDCA, Ursodeoxycholic acid; FA, formic acid adduct.

**Figure 6:**
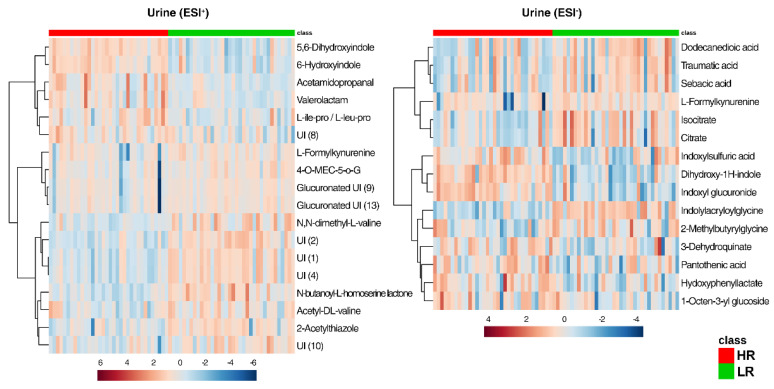
Heat maps of metabolites discriminating (*p* < 0.05) between the HR (red class) and the LR (green class) diet in the urine of Göttingen Minipigs in both positive and negative ionization mode (ESI+, ESI−). Abbreviations: 4-O-MEC-5-O-G, 4-O-methyl-epicatechin-5-O-beta-glucuronide; UI, unidentified compound.

**Figure 7:**
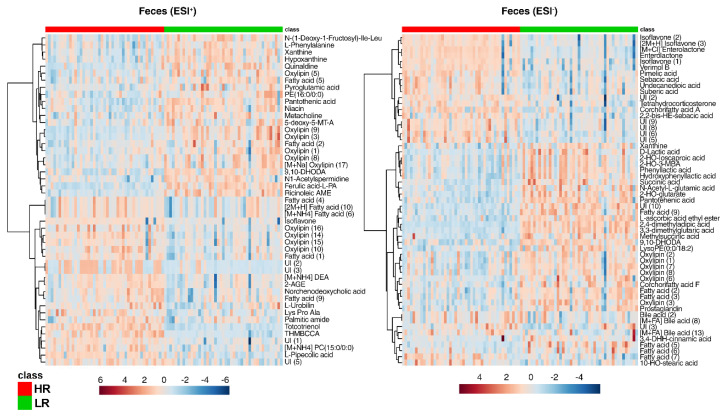
Heat maps of metabolites discriminating (*p* < 0.05) between the HR (red class) and the LR (green class) diet in feces of Göttingen Minipigs in both positive and negative ionization mode (ESI+, ESI−). Abbreviations: PE, phosphatidylethanolamine; 5-deoxy-5-MT-A, 5-deoxy-5-(methylthio)adenosine; 9,10-DHODA, 9,10-dihydroxy-octadecanedioic acid; L-PA, L-pipecolic acid; Ricinoleic AME, Ricinoleic acid methyl ester; DEA, docosatetraenoyl ethanolamide; 2-AGE, 2-arachidonyl glycerol ether; THMBCCA, tetrahydro-1-methyl-beta-carboline-3-carboxylic acid; PC, phosphatidylcholine; UI, unidentified; HO, hydroxyl; 2-HO-3-MBA, 2-hydroxy-3methylbutyric acid; LysoPE, lysophosphatidylethanolamine; DHH, dihydroxyhydro.

All metabolites identified by the sMBPLSR models (ESI+/ESI−) between the LR and HR dietary intervention are presented in Table S3. Selection based on the sparse regression coefficients revealed 66, 72, and 152 discriminating compounds found in plasma, urine, and feces, respectively. Univariate ANOVA showed 19 significantly distinct metabolites (for exact *p*-Values refer to Table S3) between the two dietary groups in plasma (Figure 5), 33 in urine (Figure 6), and 79 in feces (Figure 7). Only a few plasma metabolites were increased with the HR diet (methionine, 4-hydroxyisoleucine, betaine aldehyde, indoxyl sulfate, pantothenic acid, and vanillin-4-sulfate) compared to LR (Figure 5). The remaining metabolites were higher with the LR diet (phenylacetylglycine, fatty acid (1) (*m*/*z* 357.2800), bile acid (2) (*m*/*z* 391.2855), 2-hydroxybutyrate, L-amino-3-oxobutanoic acid, p-cresol sulfate, 3-indole propionic acid, and ursodeoxycholic acid). Ketoleucine, ketoisoleucine, and α-ketoisovaleric acid were found higher in the plasma of the LR group (Figure 5). These metabolites are found in the leucine, isoleucine, and valine catabolic pathways representing the first degradation products of the well-known branched-chain amino acids (BCAA). In ESI, phenylacetylglycine was significantly increased in the plasma of the LR group, whereas hippuric acid only presented a tendency (Table S2). These two metabolites are associated with phenylalanine metabolism.

The urinary metabolome identified increased levels of δ-valerolactam, acetamidopropanal, 6-hydroxyindole, 5,6-dihydroxyindole, isoleucyl-proline (Ile-Pro)/leucyl-proline (Leu-Pro), hydroxyphenyllactic acid, 3-dehydroquinate, indoxylsulfuric acid, pantothenic acid, and the two glucuronated compounds indoxyl glucuronide and dihydroxy-1-H-indole glucuronide in the HR diet group (Figure 6). In the urine of LR group pigs, two derived forms of valine (n,n-dimethyl-l-valine and acetyl-dl-valine) were found in higher intensity compared to the HR group. Furthermore, the LR diet led to higher levels of the glycine-conjugated compounds phenylacetylglycine, indolylacryloylglycine and the putatively identified compound *m*/*z* 158.0823 (2-methylbutyrylglucine or the isomers: isovalerylglycine, valerylglycine). Several unidentified and glucuronated unidentified compounds were associated with the LR diet (Figure 6, ESI+), together with compounds that belong to the fatty acid and conjugates metabolism, namely dodecanedioic acid, sebacic acid, traumatic acid. Isocitrate and citrate, part of the citrate cycle (TCA) metabolism, were increased with the LR diet, whereas compounds involved in the tyrosine metabolism, such as 5,6-dihydroxyindole and hydroxyphenyllactic acid, were higher with the HR diet. Similarly, 3-dehydroquinate, which is part of the phenylalanine, tyrosine, and tryptophan biosynthesis pathway, was measured higher in pigs fed the HR diet.

The fecal metabolome analyzed in ESI+ and ESI- revealed two distinct clustering patterns associated with the LR and HR diets (Figure 7). However, because the metabolites could not be specifically identified, they were instead assigned to the following classes of compounds: fatty acids, oxylipins, isoflavones, and bile acids (level 3 identification). Where possible a putative annotation was made and noted in parenthesis (Table S3). Fatty acids were the largest class group of metabolites annotated; the HR diet resulted in a higher excretion of 16 fatty acid moieties (Figure 7), whereas a total of 15 other fatty acids were elevated in the LR group. Oxylipins represented the second largest group with seven oxylipin metabolites enriched in the feces of pigs fed a HR diet and 10 oxylipins higher in the feces of pigs fed the LR diet (Figure 7). Other notable metabolites that were found higher in the HR diet compared to the LR diet were urobilin, peptide Lys-Pro-Ala, γ-tocotrienol, pipecolic acid, (±)-enterolactone, and pimelic acid. In pigs fed a LR diet, there were increased levels of phenylalanine, xanthine, hypoxanthine, quinaldine, pyroglutamic acid, pantothenic acid, niacin, N1-acetylspermidine, lactic acid, 2-hydroxyisocaproic acid, 2-hydroxyisovaleric acid, phenyllactic acid, hydroxyphenyllactic acid, n-acetyl-l-glutamic acid, 2-hydroxy-glutarate, methylsuccinic acid, succinic acid, and 3,4-dihydroxyhydrocinnamic acid.

### 2.4. Multi-Compartmental Non-Targeted Metabolomics—Time Classification

**Figure 8:**
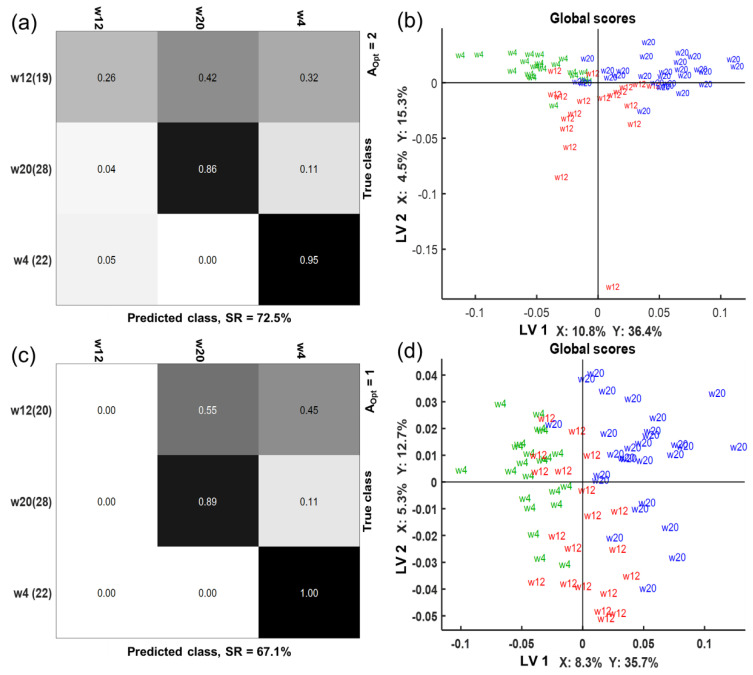
sMBPLSR confusion matrices (**a**,**c**) and global score plots (**b**,**d**) from time classification analysis using plasma, urine, and feces metabolomics in positive (**a**,**b**) and negative (**c**,**d**) ionization mode. The success rate (SR) of cross-validation is provided together with the number of latent variables of the models (A_Opt_). Numbers in parentheses (**a**,**c**) reflect the number of samples used in the multi-block analysis after a sample-to-sample correspondence. Explained variances (%) in matrices X and Y by the corresponding latent variables (LV) are provided (**b**,**d**).

For the time classification of the ESI+ and ESI− metabolomics data, two sMBPLSR models were constructed using plasma, urine, and feces as a multi-block X matrix and the time intervention period as the Y matrix (week 4, week 12, and week 20). The regression analysis resulted in good classification models with SR_CV_ = 73% for ESI+ and SR_CV_ = 67% for ESI− (Figure 8a,c). The global score plots showed that a good separation of week 4 and week 20 was obtained in the first LV in both models (Figure 8b,d). However, we observed that clustering of the metabolic profiles at week 12 was generally weaker as indicated by the sensitivity of 0.26 for the ESI+ model, and even worse sensitivity was observed for the ESI− model (Figure 8a,c). Super weights of the blocks in the sMBPLSR analysis revealed that the plasma block had the highest influence on the separation observed in LV1 in both, ESI+ and ESI− models (Figure S4).

**Figure 9:**
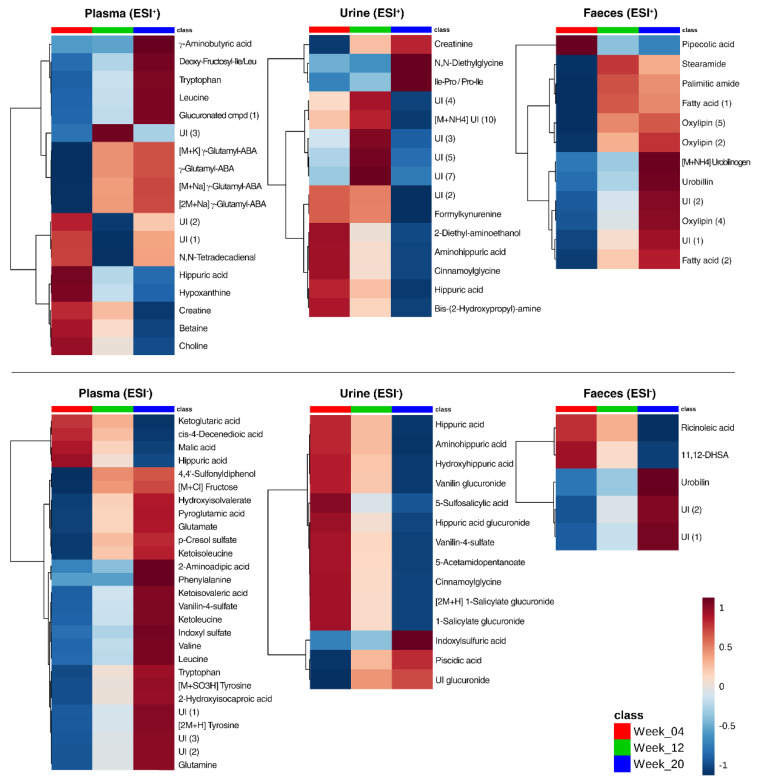
Heat map of metabolites discriminating (*p* < 0.05) between the weeks of dietary intervention in plasma, urine, and feces of Göttingen Minipigs analyzed in positive (ESI+) and negative (ESI−) ionization mode, presented as individual means per class group. Abbreviations: cmpd, compound; UI, unidentified; γ-glutamyl-ABA, γ-glutamyl-aminobutyraldehyde; 11,12-DHSA, 11,12-dihydroxystearic acid.

The selection of variables based on regression coefficients from the sMBPLSR models revealed 61, 54, and 35 discriminating metabolites between the three collection points in plasma, urine, and feces, respectively (Table S4). A heat map of significantly different features between week 4, week 12, and week 20 based on the univariate ANOVA analysis (for exact *p*-values refer to Table S4) is shown in Figure 9. Amino acid levels in plasma exhibited a time-dependent effect. The two BCAA, leucine, and valine, together with known degradation products of BCAA, such as ketoisoleucine, ketoisovaleric acid, and ketoleucine, increased in intensity over the 20-week intervention. Furthermore, tryptophan, glutamate, phenylalanine, tyrosine, and glutamine increased from week 4 to week 20 of the dietary intervention period (Figure 9). The intensities of several metabolites associated with other amino acid metabolic pathways also increased over time in plasma, namely γ-aminobutyric acid, n-(1-deoxy-1-fructosyl)-isoleucine/leucine, γ-glutamyl-γ-aminobutyraldehyde, pyroglutamic acid, hydroxyisovalerate, and 2-aminoadipic acid. In contrast, the intensity of five other plasma metabolites decreased with time: hippuric acid, ketoglutaric acid, creatine, betaine, and choline as observed in Figure 9.

Several acyl glycine compounds and derivatives were identified in urine and their intensity decreased with over time: hippuric acid, aminohippuric acid, hydroxyhippuric acid, hippuric acid glucuronide, cinnamoylglycine (Figure 9). Generally, it was observed that intensities of urinary metabolites were lower at week 20 compared to week 4, particularly formylkynurenine, 2-diethylaminoethanol, bis-(2-hydroxypropyl)-amine, vanillin glucuronide, 5-sulfosalicylic acid, vanillin-4-sulfate, 5-acetamidopentanoate, and salicylate glucuronide. Only six metabolites were increased in intensity during the dietary intervention: creatinine, n,n-diethylglycine, Ile-Pro/Leu-Pro, indoxylsulfuric acid, piscidic acid, and one unidentified glucuronide compound (*m*/*z* 345.0978; RT5.52).

The fecal metabolome contained only a few discriminating metabolites over time. The majority of those metabolites increased over time and included fatty acids and oxylipins, namely fatty acid (1) (*m*/*z* 282.2801, RT16.28), stearamide, palmitic amide, fatty acid (2) (*m*/*z* 283.2639, RT14.41), oxylipin (5) (*m*/*z* 319.2253, RT13.97), oxylipin (2) (*m*/*z* 295.2277, RT13.42), and oxylipin (4) (*m*/*z* 299.2589, RT14.88). Urobilinogen, urobilin, UI (1), and UI (2) in ESI+, UI (1) and UI (2) in ESI− were also found to increase over time. Conversely, pipecolic acid, ricinoleic acid, and 11,12-dihydroxystearic acid decreased in intensity over time.

### 2.5. Correlation of Variables in a Multi-Block Analysis of Fecal Microbiota, Metabolome, and SCFA

**Figure 10:**
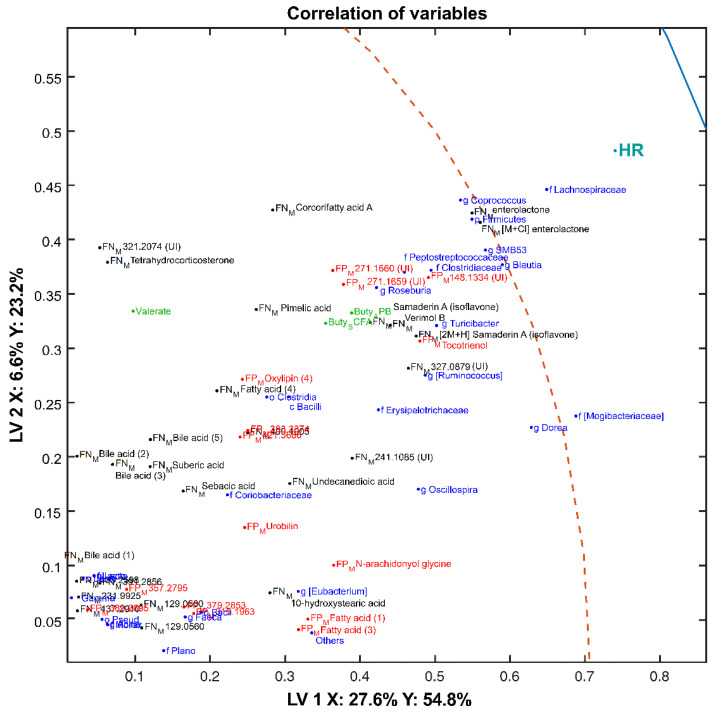
A detailed view of the correlation loading plot focusing on variables strongly associated with the HR diet (for a global overview of the correlation plot, we refer to Figure S5). Scores of a 4-block model: fecal ESI+ metabolomics (FP_M_, red), fecal ESI− metabolomics (FN_M_, black), SCFAs (green), metataxonomics (blue) are used to plot the correlations. The first two components are shown with the explained variances in sample matrix X and the dietary treatment Y. Abbreviations: g, genus; f, family; o, order; k, kingdom; p, phylum; c, class; UI, unidentified compounds.

To better understand the relationship between the fecal microbiota, fecal SCFA concentrations, and the fecal metabolome, an sMBPLSR model combining these data blocks was established for the classification of the two diets. Details on model performance can be observed in Figure S5, and metabolites selected based on regression coefficients are found in Table S5. The classification model showed good separation of the HR and LR groups along LV1 (Figure S5b) with a SR_CV_ = 95.3% (Figure S5a). The superweight plot revealed that bacterial composition and SCFA concentrations contributed most to the separation of the groups followed by the fecal metabolome in ESI− (Figure S5c). A correlation loading plot is provided to visualize the co-variation tendencies of variables from the four different blocks (Figure S6). Based on the distances between variables in the correlation loading plot (Figure 10), variables clustering together are strongly correlated to each other. On the other hand, variables distant in the LV1 and LV2 spaces present a weaker correlation. This is illustrated in Figures S7 and S8 where the correlations among variables are presented as a heat map.

Certain metabolites, microbiota, and SCFAs correlated with the HR diet (Figure 10). We observed that levels of members of the *Firmicutes* phylum, *Lachnospiraceae* family, and the genera *Coprococcus*, *Blautia*, *and* SMB53 were positively correlated with enterolactone (*p* < 0.001) and the [M + Cl] enterolactone adduct (*p* < 0.001). Bacteria from the *Clostridiaceae* family and the *Roseburia*, *Turicibacter* genera positively correlated with butyric acid (as a proportion of total SCFA and APB) and fecal metabolites such as Verimol B (*m*/*z* 315.1242), putatively annotated isoflavone Samaderin A (isoflavone (1), *m*/*z* 329.1034; isoflavone (2), [2M + H] *m*/*z* 329.1034), tocotrienol (*m*/*z* 411.3269), pimelic acid (*m*/*z* 159.0666), and some unidentified compounds (*m*/*z* 327.0879, *m*/*z* 271.1660, *m*/*z* 270.1659, *m*/*z* 148.1334). Members of the *Clostridiales* and *Bacilli* were closely correlated to each other but also positively correlated with oxylipin (4) (*m*/*z* 319.2253) and fatty acid (4) (*m*/*z* 315.2538).

**Figure 11:**
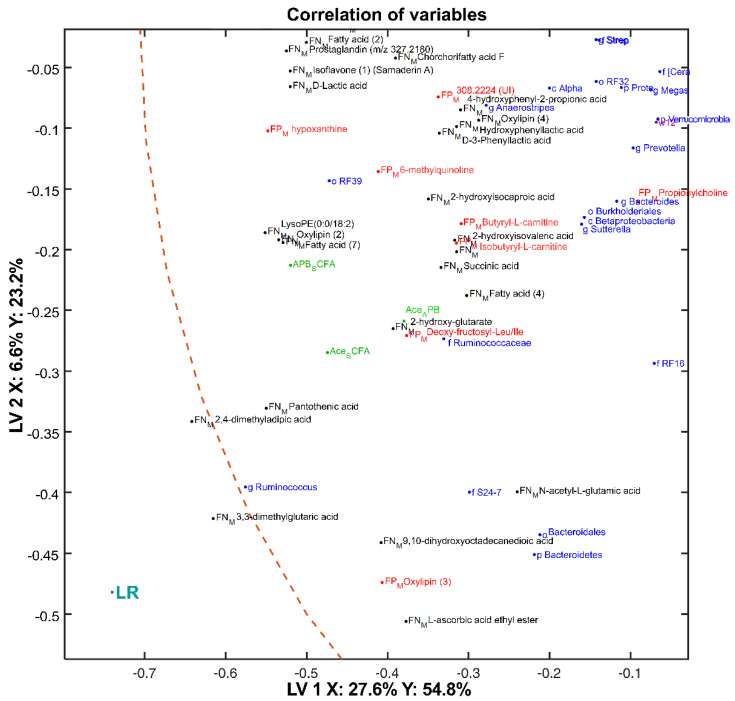
A detailed view of the correlation loading plot focusing on variables strongly associated with the LR diet (for a global overview of the correlation plot, we refer to Figure S5). Scores of a 4-block model: fecal ESI+ metabolomics (FP_M_, red), fecal ESI− metabolomics (FN_M_, black), SCFAs (green), metataxonomics (blue) are used to plot the correlations. The first two components are shown with the explained variances in sample matrix X and the dietary treatment Y. Abbreviations: g, genus; f, family; o, order; k, kingdom; p, phylum; c, class; UI, unidentified compounds.

Figure 11 provides an overview of the metabolites, microbiota, and SCFAs strongly correlated with LR diet consumption. Similar to the previous analysis, the LV1 and LV2 distances in the correlation of variables plot (Figure 11) were analyzed to determine the degree of correlation and presented in Figure S8 in the form of a heat map. Proportions of bacteria in the *Ruminococcus* genus were positively correlated with 3,3-dimethylglutaric acid (*m*/*z* 159.0666), 2,4-dimethyladipic acid (*m*/*z* 173.0822), and pantothenic acid (*m*/*z* 218.1037). In addition, *Bacteroidetes*, *Bacteroidales,* and members of uncultured S24_7 were similarly correlated with the following metabolites: n-acetyl-l-glutamic acid (*m*/*z* 188.0566), 9,10-dihydroxyoctadecanedioic acid (*m*/*z* 345.2285), l-ascorbic acid ethyl ester (*m*/*z* 203.0564), and Oxylipin (3). Furthermore, members of the *Ruminococcaceae* family were strongly positively correlated to n-1-deoxy-1-fructosyl-ile/leu (*m*/*z* 294.1546), 2-hydroxy-glutarate (*m*/*z* 147.0300), and the fecal acetate (proportion of total SCFA and proportion of A + P + B). Bacteria from the RF39 order were closely associated with hypoxanthine (*m*/*z* 137.0457), 6-methylquinoline (*m*/*z* 144.0811), 2-hydroxy-isocaproic acid (*m*/*z* 131.0716), 2-hydroxy-isovaleric acid (*m*/*z* 117.0559), and a series of fatty acid compounds: fatty acid (7) (*m*/*z* 537.3285), LysoPE (0:0/18:2(9Z,12Z)) (*m*/*z* 479.3016), oxylipin (2) (*m*/*z* 311.2230).

## 3. Discussion

In this study, we explored the metabolic fingerprints of plasma, urine, and feces together with the microbiota composition of feces in a longitudinal study with juvenile Göttingen Minipigs. Given the high-fat-high-carbohydrate nature of the two diets, the focus of the study was on the higher degree of risk presented by the fructose ingredient when compared to a combination of digestible and fermentable starch from HiMaize, which presents a lower risk of developing obesity and signs of MetS based on the known physiological and chemical effects. Contrary to our expectations regarding RS action and suppression of appetite, the *ad libitum* feeding strategy resulted in obesity development and increasing insulin and glucose levels over time in both groups, without changes in dyslipidemia markers between fructose and HiMaize [8]. Further, it was shown that fructose did not accelerate MetS development, but it induced an upregulated gene response in the liver tissue for C-reactive protein and chemokine ligand 5 (CCL5) genes [8]. Although no clinical biomarkers of disease were differently affected by the two carbohydrate sources [8], the multi-compartmental metabolomics analysis presented here revealed several differences in the metabolome of plasma, urine, and feces between minipigs fed these diets.

The diet classification analysis using sMBPLSR revealed that, among all data blocks, plasma was mostly affected by diet. Degradation products of BCAA, i.e., α-ketoisovaleric acid, ketoleucine, and ketoisoleucine, were increased in the plasma of pigs fed the LR diet. In addition, we detected higher levels of two valine-derived compounds (n,n-dimethyl-l-valine and acetyl-dl-valine) in the urine of the LR group. Presently, increased blood levels of circulating BCAA are considered well-established metabolic biomarkers for the development of diabetes [22,23].

Decreased activities and levels of tissue-specific branched-chain amino acid transaminase (BCAT) and branched-chain α-keto acid dehydrogenase complex (BCKDH) have been observed in obese Yucatan minipigs [24] and in ob/ob mice models [25]. In this study, no diet difference was observed for plasma BCAA, but the catabolism products of BCAA were increased with the LR diet. It might be of future interest to explore in more depth the mechanism involving HiMaize and the effects on keto acids and enzymes involved in BCAA catabolism. The presence of BCAA and BCAA catabolism products has also been linked to the time progression of obesity in this minipig model and is further discussed in the subsequent paragraphs.

Other differences between the two dietary groups revealed pantothenic acid (PA) to be significantly altered in all the explored metabolic compartments. PA plays an important role in coenzyme A synthesis and has a direct impact on the TCA cycle through acetyl-CoA. Higher levels of PA were observed in plasma and urine with the HR diet, but lower levels were found in feces when compared to the LR diet. Increased intensity of PA in the feces of the LR pigs could be attributed to the higher abundance of bacteria from the *Bacteroidetes* phylum, which is better at PA synthesis than *Firmicutes* based on genome functionality analysis [26]. Urinary excretion of PA and TCA cycle intermediates (citrate, isocitrate, malic acid) has been suggested as a protective mechanism against the effects of energy-dense diets by controlling carbon-containing molecules available for lipogenesis and fat accumulation [24,27,28]. Interestingly, the urinary levels of isocitrate and citrate were higher in the LR group but, on the other hand, PA was not.

The time classification sMBPLSR models provided valuable information about the metabolome evolution of the Göttingen Minipigs under a longitudinal aspect over 5 months of dietary intervention. Interestingly, the classification of metabolomics data at week 12 was poor according to the regression models pointing toward a less distinct metabotype when compared to week 20 of dietary intervention. Previously analyzed urinary metabolome of Yucatan minipigs showed that the first two weeks of dietary intervention have a substantial impact on the metabolic adaptation mechanisms to an obesogenic diet, followed by a period that is less metabolically distinguishable [24]. This miss-classification result indicates that the development of obesity is a lengthy process even in *ad libitum* fed Göttingen Minipigs, and possibly reversible at the stage between 4 and 12 weeks but not at 20 weeks on a high-fat-high-carbohydrate diet.

Irrespective of diet, the time-course analysis showed an accumulation of BCAA (leucine, valine) and metabolism products (ketoisoleucine, ketoisovaleric acid, 2-hydroxyisovaleric acid, and ketoleucine) in plasma from week 4 to week 20, similar to what has been described in a previous Yucatan minipig study [29]. Serum of Yucatan minipigs that were fed high-fat-high-sucrose diets *ad libitum* exhibited no upregulation of BCAA or degradation products compared to a restricted diet [30]. This suggests that although BCAA are essential amino acids, their relation with obesity development is linked more to diet composition rather than feed intake. Metabolomics on large cohort studies in humans [22,31] revealed several other biomarkers associated with the development of diabetes such as the amino acids tyrosine, phenylalanine, and the lysine degradation product 2-aminoadipic acid. All three metabolites were increased in plasma from week 4 to week 20 of the dietary intervention in our study.

Other amino acids and degradation products also showed a time-dependent variation reflected in two interconnected pathways: (1) alanine, aspartate, and glutamate metabolism, and (2) glutamine and glutamate metabolism. The link between them is γ-aminobutyric acid, which can be derived enzymatically from glutamate or γ-glutamyl-γ-aminobutyraldehyde. These metabolites (γ-aminobutyric acid, glutamate, and γ-glutamyl-γ-aminobutyraldehyde) were observed to increase with exposure to the diets. Furthermore, glutamine (Gln), an important metabolite in amino acid transamination [32], was also increased with time. Gln has previously been associated with obesity [30,33] and diabetes [33] as Gln plays a valuable role in gluconeogenesis and insulin secretion [34]. The higher levels of Gln and associated metabolites could be described as a systemic response to a higher concentration of plasma glucose in an attempt to stimulate insulin secretion and decrease glucose levels [34]. Increased glutamate exportation from the liver to the peripheral circulation has been observed in Yucatan minipigs on high-fat-high-sugar diets, indicating a potential nitrogen sparing mechanism which has been described in a fasting phase but also in a postprandial phase [35,36]. Similar to the time-dependent body weight development in our study, glutamate release from the liver was positively correlated with homeostatic model assessment of insulin resistance (HOMA-IR) measurements and body weight of obese Yucatan minipigs [36]. Further, glutamate and glutamine are important substrates for epithelium regeneration, expansion, and turn over, and arterial-venous balance was increased with time in obese Yucatan minipigs [35].

Significant changes in the gut environment were observed as a response to the two different carbohydrate substrates. Although the effects of RS consumption in humans and animal models on the microbiome and metabolome has been examined [37], less is known about the effects of fructose-rich diets on Göttingen Minipigs metabolome and microbiota. Firstly, although there were diet-specific differences in fecal bacterial species richness and diversity, those differences remained constant throughout the experiment, suggesting that the adaptation to the two carbohydrate sources already had taken place prior to the first sample collection at week 4. Secondly, a lower species richness (ASVs count) associated with the LR diet compared to HR was observed, presumably because of the specialized capacity of certain intestinal bacteria to utilize RS [37,38] compared to the more generalized metabolism of other diet components that escape intestinal absorption.

A higher relative abundance of *Bacteroidetes* and S24_7 in LR-fed pigs is consistent with studies on the high intake of RS in humans, swine, and rodents [37]. These bacterial species have a higher capacity to bind to and metabolize starch through the presence of multiple polysaccharide utilization loci, which encode for a glycan-uptake system, namely the starch utilization system (Sus). This system has been well-studied in *Bacteroides thetaiotamicron* and is analogous in all mammalian gut *Bacteoridetes* [38]. Generally, pigs fed the LR diet contained higher total SCFAs, total organic acids, and total APB in the feces compared to those fed the HR diet in accordance with previous studies on RS in swine using HiMaize starch [39,40]. In this study, however, the bacteria enriched with the LR diet were less associated with beneficial butyrate and more with an increased fecal acetate production. Strong positive associations were found between acetate and species from the *Ruminococcus* genus. To that regard, *Ruminococcus bromii* is considered a primary degrader of RS and a known producer of acetate together with other *Bacteroides* species [41]. Butyrate-producing *Eubacterium rectale* has been positively correlated with a change in abundance of *R. bromii*, indicating a cross-feeding mechanism and utilization of acetate for butyrate production [42]. Butyrate levels as a proportion of APB and total SCFA were higher in the pigs fed the HR diet and were correlated with several taxonomic groups: *Roseburia*, *Clostridiaceae*, *Turicibacter*, and *Ruminococcus* (*Lachnospiraceae* family). *Roseburia* and *Turicibacter* as well as other members of the *Lachnospiraceae* have been previously enriched in a rat model of obesity fed high levels of fructose [43]. The difference in SCFA found in feces was also reflected in the plasma of the LR group as concentrations of total SCFA, total BCFA, and total ABP were higher when compared to the HR diet. Plasma concentrations of acetate were also increased by the intake of HiMaize. Recently, a rodent study revealed how acetate might aid in promoting hyperphagia and weight gain [44]. The acetate-mediated hyperphagia in an *ad libitum* context could explain why the LR-fed pigs had a higher feed intake than pigs fed the HR diet, despite having significantly higher levels of circulating PYY [8]. It is interesting to note that a slight increase in plasma propionic acid occurred with the LR diet, which is not directly explained by a rise in fecal concentrations of propionic acid, as observed for acetic acid or total SCFA. It is known that the liver plays an important role in metabolizing SCFAs from portal blood and thus the liver is exposed to propionate as a gluconeogenesis precursor [45]. Given the slightly higher levels of systemic propionate with the LR diet, we speculate that the extra load of rapidly digestible glucose from HiMaize reduces the liver gluconeogenesis from propionate.

Finally, explorative analysis of the fecal metabolome identified correlations between bacteria found in the LR group and a series of fecal metabolites, many of which can be categorized as dicarboxylic acids (2,4-dimethyladipic acid, 3,3-dimethylglutaric acid, n-acetyl-l-glutamic acid, 2-hydroxy-glutarate, and succinic acid). Succinate is an anaerobic fermentation by-product of bacteria in the *Bacteroidetes* phylum [46] and is usually regarded as an intermediary metabolite. Recently, however, succinate was proposed as a substrate for intestinal gluconeogenesis, inhibiting hepatic glucose release and improving glucose and energy metabolism in rats [47]. Although we cannot account for the improved glucose or insulin homeostasis related to the LR diet [8], the presence of succinate or other dicarboxylic acids should not be disregarded. Conflicting results exist, as an increased accumulation of succinate in feces has been associated with certain pathophysiological situations [48], especially with cases of inflammation and metabolic stress, which in this case could be well attributed to the continuous intake of high-energy-high-fat diets [49]. Evidence from obese Yucatan minipigs supports this idea, as the intestinal release of succinate was increased in obese animals compared to a neutral arterial-venous exchange during a healthy condition [36]. Furthermore, the liver uptake of succinate has been positively correlated with HOMA-IR [36]. Regarding the HR diet, several bacterial species were correlated to the presence of enterolactone (Enl). Through dehydrogenation by colonic microbiota, enterodiol is converted to Enl, a principal degradation product of plant lignans [50]. We believe that the presence of Enl might reflect a colonic degradation of other dietary components that escape intestinal absorption, particularly plant lignans from whole grain wheat or wheat bran within the HR diet.

## 4. Materials and Methods

### 4.1. Diets, Animals, and Experimental Design

Animal handling and experimental procedures were done in accordance with the license obtained from Danish Animal Experimentation Inspectorate, Ministry of Food, Agriculture, and Fisheries. The intervention study followed the Danish laws and regulations regarding the humane care and use of animals in research (The Danish Ministry of Justice, Act on Animal Experiments no. 474 of 15 May 2014, as stipulated in the executive order no. 12 or 7 January 2016).

Two diets were formulated for the study of long-term effects (20 weeks) of high-fat diets supplemented with either fructose in the HR diet or polymeric glucose from high amylose maize resistant starch in the LR diet. On a weight-basis, the diets were similar in crude protein, fat, and ash, and deviated only in the exchange of 20% Hi-Maize© (Ingredion UK Limited, Manchester, UK) with 20% fructose (Table S6). The relative energy contribution of the HR diet components were 34.6% from fat, 10.3% from protein, and 4.3% from total dietary fiber, whereas the LR diet contributed with 37.9% energy from fat, 11.7% energy from protein, and 8.7% energy from total dietary fiber (Table 1). The fat source used in this experiment was lard and contained the following fatty acid profile: 45.1% saturated, 45.1% monounsaturated, and 9.8% poly-unsaturated fatty acids (Table S6).

Thirty female Göttingen Minipigs (Ellegaard Göttingen Minipigs, Dalmose, Denmark) used in this experiment were delivered at 8 weeks of age. For the first week after arrival, the pigs had access to a restricted fed standard SDS (Special Diet Services, Dietex International, Witham, UK) chow, followed by a gradual transition to the experimental diets lasting one additional week. Fifteen animals were allotted to each group and diets were provided *ad libitum* for a period of 20 weeks, during which the animals were housed individually, in visual and physical contact with each other, but separated by metal railings. Animals were provided wood shavings for bedding and had *ad libitum* access to water.

Further details on the experimental design, diets, and main outcomes of the experiment related to obesity development and signs of metabolic syndrome are described by Curtasu et al. (2019) [8].

### 4.2. Sample Collection

At 4, 12, and 20 weeks of the dietary intervention, the animals were fasted overnight (16 h), anesthetized, and jugular blood plasma for metabolomics was collected using 6 mL LiHep tubes, centrifuged for 12 min at 3300 rpm and 4 °C, separated into aliquots and stored at −80 °C. Before the procedures, fresh feces were collected from the animals (natural bowel movement as an effect of anesthesia), frozen at −20 °C, and stored until analysis at −80 °C. Spot urine samples were collected using absorbent tampons, placed at the rear of the minipigs using Omniplast adhesive fabric tape (Hartmann, Heidenheim, Germany). While of great importance for the study, sample collection at baseline (week 0) was avoided due to safety and welfare considerations toward the minipigs given their age and size. Lack of blood, tissue biopsies, and other organic material at the start of intervention are considered a limitation of this study.

### 4.3. Fecal DNA Extraction, 16S rRNA Gene Sequencing, and Microbiota Data Analysis

Bacterial DNA was extracted from frozen fecal material using a QIAamp Fast DNA Stool Mini Kit (Qiagen, Hilden, Germany) with several modifications as previously described [51]. After purification, DNA concentration was measured using a NanoDrop spectrophotometer (Thermo Fisher Scientific, Waltham, MA, USA) diluted to 20 ng/µL. PCR was used to amplify the 16S rRNA V4 regions using DNA and barcoded F515 and the R806 primers as previously described [51]. The PCR products were pooled, purified using the Wizard SV gel and PCR Clean-up System (Promega, Madison, WI, USA), and the DNA concentration was measured on a Qubit fluorometer (Thermo Fisher Scientific, Waltham, MA, USA). The purified amplicons were sequenced on Illumina MiSeq using the PE300 protocol (16S amplicon, 290 bp insert size, paired-end library with overlapping reads, and ligated adapters prior to sequencing) at the Genome Center DNA Technologies Core, University of California, Davis, CA, USA (https://dnatech.genomecenter.ucdavis.edu/).

The DNA sequences files were analyzed with Quantitative Insights into Microbial Ecology 2 (QIIME2) [52]. Although paired-end sequencing was performed, the alignment of the reads was not done. R1 and R2 files were combined and single-ended analyses were performed on the reads. Barcodes sequences were extracted from the reads and used for demultiplexing. The DADA2 plugin [53] was used to identify single-nucleotide resolved Amplicon Sequence Variants (ASVs) within the demultiplexed sequences. A rooted phylogenetic tree was generated based on multiple sequence alignments of the ASVs performed with MAFFT [54]. Alpha diversity rarefaction curves were generated by using the Chao1 diversity index, Phylogenetic Diversity Whole Tree, and observed taxonomic units (OTUs) metrics, determined by the change in numbers of randomly sampled sequences per sample (sampling depth). Asymptotic curves were observed at 25,480, 22,216, and 24,839 DNA sequences per sample at weeks 4, 12, and 20, respectively. These numbers of randomly sampled DNA sequences were used for rarefication. For the beta-diversity, the weighted UniFrac metric was calculated using rarefied data and constructed in QIIME2 and diet groups compared using the PERMANOVA group significance test. For taxonomy classification, the Scikit-learn classifier (classify-sklearn) using the GreenGenes database version 13-8 clustered at 99% was used. Comparisons between bacterial taxa were performed by LefSe analysis (Linear discriminant analysis Effect Size), as described by Segata et al. [55] in the Galaxy online module, with an alpha value for the Kruskal-Wallis test set to 0.01 and a linear discriminant analysis (LDA) log threshold for discriminative features set to 2.0.

### 4.4. Fecal and Plasma Short-Chain Fatty Acid (SCFA) Analysis

Fecal SCFA concentrations were determined by gas-liquid chromatography (HP-6890 Series Gas Chromatography, Hewlett Packard, Palo Alto, CA, USA) according to Canibe et al. [56]. Total fecal SCFA concentration was calculated as the sum of formic acid, acetic acid, propionic acid, isobutyric acid, n-butyric acid, isovaleric acid, and n-valeric acid concentrations. Total fecal organic acids were calculated as the sum of total SCFA and isocaproic acid, n-caproic acid, heptanoic acid, sorbic acid, benzoic acid, DL-lactic acid, succinic acid, and hippuric acid. Total fecal branched-chain fatty acids (BCFA) concentration was calculated as the sum of isobutyric acid, isovaleric acid, and isocapronic acid.

Plasma SCFA concentrations were determined by LC-MS/MS according to Han et al. (2015) [57] with modifications. Ten µL of 75% methanol containing stable isotope internal standards (^13^C_2_ acetic acid, ^13^C_1_ propionic acid, ^13^C_2_ butyric acid, ^13^C_2_ succinic acid, and ^13^C_3_ valeric acid) were mixed with 10 µL of plasma samples, 10 µL of 200 mM 3-nitrophenylhydrazine hydrochloride (3NPH), and 10 µL of 120 mM N-(3-dimethylaminopropyl)-N’-ethylcarbodiimide hydrochloride (EDC). The mixture was shaken for 45 min at room temperature, and 10 µL of 200 mM of Quinic acid was added to quench derivatization. The mixture was shaken for 15 min at room temperature, and 950 µL of 10% methanol was added and mixed, centrifuged for 20 min at 4 °C 29,700× *g,* and 700 µL of supernatant was transferred into HPLC vials. Samples were analyzed on microLC 200 from Eksigent (Framingham, MA, USA) using C18 column (10 cm × 1 mm, 1.2 µm particle size) from Waters (Waters Corporation Milford, MA, USA). The chromatographic conditions started at 5% solvent B (acetonitrile), kept for 1 min, the gradient increased for 4 min to 27% solvent B, then increased for 5 min to 30% solvent B and to 37% solvent B during 5 min. Solvent A was pure water. The total chromatographic run was 15 min, with a flow of 50 µL/min, the column oven set to 30 °C, and the injection volume was 5 µL. MicroLC was interfaced with QTrap 5500 from ABSciex (Framingham, MA, USA) using electrospray ionization (ESI) in negative mode. The flow injection analysis (FIA) was performed to optimize the turbo V source of the instrument, where curtain gas was set to 30 psig, nebulizer gas (Gas1) 50 psig, heater gas (Gas2) 50 psig, temperature was 500 °C, ionization spray operated at −4500 eV, and collision Gas was set to High. Deprotonated molecules were detected in the Multiple Reaction Monitoring (MRM) mode. The compound-dependent parameters were optimized by syringe infusion of pure standards and shown in Table S7. The entrance potential was set to −10 volts and the time of MRM scan to 30 msec for all the compounds. The data analysis was performed in Analyst software 1.6.1 from ABSciex (Framingham, MA, USA). The representative chromatogram is presented in Figure S9.

Total plasma SCFA concentration was calculated as the sum of acetic acid, propionic acid, butyric acid, valeric acid, isobutyric acid, and isovaleric acid. Total fecal organic acids were calculated as the sum of total SCFA and succinic acid. Total plasma BCFA concentration was calculated as the sum of isobutyric acid and isovaleric acid.

### 4.5. Metabolomics Sample Preparation, Ultra-High Performance Liquid Chromatography-Mass Spectrometry (UHPLC/MS)

Blood plasma was prepared by deproteinization of 150 µL sample with 450 µL ice-cold acetonitrile (100% ACN) containing an internal standard mix of glycocholic acid (glycine-1-^13^C) and p-chlorophenylalanine to a final concentration of 0.01 mg/mL. Samples were prepared in 96-well plates with 1 mL wells. Plates were mixed for 1 min, incubated at 4 °C for 10 min, and centrifuged for 25 min at 2250× *g* and 4 °C. Approximatively 400 µL supernatant was transferred to Sirocco Protein precipitation plates (Waters Corporation, Milford, MA, USA). The filtered supernatant was transferred to two 200 µL 96-well plates (65 µL per well), and plates were vacuum centrifuged to dryness (ca. 2.5 h, 805× *g* and 30 °C). Resuspension of the samples was done in a mix of H_2_O:ACN:FA (95:5:0.1) using the same volume before evaporation. A protective film was welded on the plate using a heat sealer, and the plates were centrifuged at 3700 rpm, 4 °C for 25 min before the LC-MS analysis.

Urine samples (180 µL) were mixed with 20 µL ice-cold 100% ACN containing the same internal standard mix as for plasma. After an immediate vortex, the samples were incubated 20 min at 4 °C for protein precipitation. Samples were centrifuged at 13,200× *g* for 10 min; the supernatant was collected and transferred to LC-MS vials with inserts.

Prior to sample preparation, feces were freeze-dried and mechanically milled. Fecal material (50 mg dried samples) was mixed with 500 µL ice-cold 50% ACN with added internal standards and vortexed for 15 min at room temperature. Samples were individually ultra-sonicated using a needle sonicator at 100% amplitude, 0.5 cycles, and 10 pulses (IKA-Werke GmbH & Co. KG, Staufen, Germany). Samples were vortexed for 5 min and set for incubation at 4 °C for 15 min. After a 10-min centrifugation (425× *g*, 4 °C), 200 µL supernatant was transferred to a 10 K Omega filter Nanosep (Pall Laboratory, Pall Corporation, Westborough, MA, USA) and centrifuged until all supernatant was passed through the filter. The filtrate was transferred to LC-MS vials with inserts.

UPLC-MS analysis was performed using a Dionex UltiMate 3000 (Dionex, Sunnyvale, CA, USA) ultra-high pressure liquid chromatography system (UHPLC) coupled with an Impact HD Quadrupole Time-of-Flight (QTOF) mass spectrometer (Bruker Daltonics GmbH, Bremen, Germany) operating in positive electrospray ionization mode (ESI+) and negative electrospray ionization mode (ESI−) using the instrumental parameters described by Curtasu et al. [30].

The chromatographic separation of compounds was performed on an Acquity HSS T3 1.7 µm 100 × 2.1 mm (Waters Corporation, Milford, MA, USA) column equipped with a VanGuard Pre-column, 100 Å, 1.8 µm, 2.1 mm × 5 mm (Waters Corporation, Milford, MA, USA). The column temperature was set to 30 °C, samples were kept in the autosampler at 10 °C for the whole duration of the analysis, and the injection volume was set to 3 µL for plasma, urine, and feces. The chromatographic analysis was performed under a gradient of water/formic acid (100:0.1, *v*/*v*, mobile phase A) and acetonitrile/formic acid (100:0.1, *v*/*v*, mobile phase B). For the analysis of plasma, a linear gradient was used from 5% B to 100% B over 12 min, and 1 min hold at 100%. Urine was analyzed using a multi-step gradient as follows: 5% B to 70% B for 8 min, 70% B to 100% B for 0.5 min, and hold at 100% B for 1 min; gradient was returned to 5% over 0.2 min. For feces analysis, a linear gradient was used from 5% B to 100% B over 17 min and a 1 min hold at 100% followed by a return to 5% over 0.2 min. In all three methods, the column was equilibrated for 2 min at 5% B and the flow rate was set to 400 µL × min^−1^.

### 4.6. Sample Quality Control and Metabolomics Data Pre-Processing

The quality of the chromatographic runs, the UPLC system stability, and the accuracy of sample preparation were monitored using quality control samples (QCs). Serum and urine QCs were prepared by pooling an aliquot amount of samples and subjecting them to the same sample preparation protocol as the samples. The feces QCs were prepared by pipetting aliquots of each sample after sample sonication and centrifugation. The QCs were injected multiple times throughout the analysis as well as at the beginning and end of the analysis and used in the data pre-processing for signal drift correction. Blanks were injected during the chromatographic analysis to monitor any external contaminants from solvents, eluents, and carry-over effects. The sample order was randomized for the chromatographic analysis to eliminate biases in the results and to ensure that each sample group is affected equally.

Mass spectra were calibrated and converted into the mzXML file format. R based XCMS package [58] was used for the extraction of the mass features; peak peaking was performed using ‘centWave’ method and retention time aligned using ‘Obiwarp’; missing values were substituted using the ‘fillPeaks’ method; adducts, fragments, and isotopes were annotated using CAMERA. Exported data tables were filtered to eliminate features present in blanks, retention times were truncated to contain only portions with chromatographic peaks, and masses higher than 700 *m*/*z* were discarded. Plasma, urine, and fecal metabolomics data were normalized using the Van der Kloet [59] procedure based on the quality control samples.

### 4.7. Chemical Solvents and Standards for Metabolomics Analysis

High-performance liquid chromatography (HPLC)-grade solvents and eluents were used for the untargeted metabolomics analysis as follows: HPLC-grade acetonitrile (VWR, West Chester, PA, USA), formic acid (FA, Fluka, Merck KGaA, Darmstadt, Germany), and MilliQ grade water (MilliporeSigma, Burlington, MA, USA). Internal standards included during the sample preparation were Glycocholic acid (Glycine-1-13C), and 4-chloro-DL-phenylalanine (Sigma, Merck KGaA, Darmstadt, Germany) and all other standards used for compound identification were purchased from Sigma-Aldrich (Merck KGaA, Darmstadt, Germany) and Cayman Chemical (Ann Arbor, MI, USA).

### 4.8. Multivariate Data Analysis

Prior to any analysis, all metabolomics datasets were pre-treated by Pareto scaling [60], and preliminary data mining for an overview was performed using consensus principal component analysis (CPCA) [61].

A multivariate classification analysis using sparse (multi-block) Partial Least Squares Regression (sMBPLSR) was utilized in this paper to (a) classify the samples into two diets (LR, HR) and (b) classify the samples into three time points (week 4, week 12, week 20). This sMBPLSR variable selection method allows for the discovery of relevant biomarkers by putting a threshold on loading weight vectors and thus making them sparse. The method was introduced for ordinary PLSR with X (regressors, descriptor variables) and Y (regressands, response variables) blocks by Le Cao [62], improved by Karaman [63], and extended to multi-block [64]. The PLSR method [65] is a widely used dimensionality reduction method that finds new components represented by so-called latent variables for data matrices X and Y. For each component, the PLS model maximizes the co-variance of X and Y, and thereby finds directions of strong co-variation patterns in X and Y. Matrix X here represents metabolomics/metagenomics/SCFAs data or a combination of those. Matrix Y in the case of classification analysis contains indicator variables (0/1 variables), reflecting the groups’ memberships.

The first multi-block analysis was performed using only metabolomics data, where three blocks of feces, plasma, and urine (in both ESI+ and ESI−) were used to classify samples into two diets and three time points. Secondly, four blocks including fecal metabolomics (both ESI+ and ESI−) in combination with SCFAs and metataxonomics data were used to classify samples into two diets. To perform multi-block analysis for all data blocks, a sample-to-sample correspondence needs to be established [64]. Thus, any missing samples in one block were removed in all other blocks. In addition, for every multiblock analysis, each X block was normalized by its Frobenius norm, which equals a square root of the sum of squared values of each matrix entry. Scaling is done to set all the blocks on the same footing so that the measurement unit of a particular block will not have any influence on the MBPLSR model. Even though different blocks of data had a substantially different number of variables, this fact did not influence the analysis’ results. Thus, scaling each block by the number of its variables was not implemented.

In the process of building classification models, there were several parameters to be optimized. The sparsity of the final models is defined by the parameter called the degree of sparsity [63], which is optimized for each component in a model. The degree of sparsity, which yields the minimum misclassification rate (MCR) for classification or minimum root mean square error (RMSE) for regression, was selected as optimal. Then, a number of latent variables were optimized: for each number of latent variables, MCR or RMSE were calculated. The optimal number, A_Opt_, is chosen as the smallest number which does not yield a significantly higher MCR/RMSE than the minMCR/minRMSE. For optimization of all parameters, error estimations, MCR, and RMSE were calculated using leave-one pig-out cross-validation (CV) procedure. Success rate (SR) and R^2^ of cross-validation (SR_CV_ and R_CV_) are used to specify models’ performances. Since the datasets are of moderate size, no external validation was done. However, the selected CV strategy is the most reasonable in this case and provides a good estimation of the models’ performances.

Selected biomarkers of each block were tested for statistical significance in differentiating the corresponding groups (diets and time). To do this, one-way analysis of variance (ANOVA) was applied to the log-transformed columns of each block, which represent the preselected biomarkers. The log transformation is done to compensate for large differences among variables in each block. ANOVA allows comparing the means of groups and returns the p-value for the null hypothesis that the means of the groups are equal. *p*-values ≤ 0.05 were considered significant.

To learn about the correlations among variables representing selected biomarkers of metabolomics, SCFA and microbiota, and other experimental design variables, correlation loading plots were used. To obtain a correlation loading plot, scores of the corresponding model are used and the variables of interest are plotted together with the scores. To statistically test the correlations, correlation heat maps were plotted indicating the statistical significance of the preselected biomarkers. The selection of biomarkers was based on the closeness to the corresponding diets in the score plot. In order to calculate the correlations and statistical significance, the data were represented using only the first two LVs. By this representation, we focus on the first two dimensions which are summarized in the corresponding score plot, while other dimensions are neglected. The total correlation pattern might be slightly different when more LVs are included, but in this study, we focused on the two most important LVs, and thus the main correlation pattern must be captured using these LVs.

Multivariate sMBPLSR and ANOVA analyses were performed by built-in and developed in-house algorithms in Matlab, R2018a (The Mathworks Inc., Natick, MA, USA).

### 4.9. SCFA and Alpha Diversity Statistical Analysis

Differences between the two diets regarding fecal and plasma SCFA and alpha diversity were assessed using Statistical Analysis Software (SAS, version 9.4, SAS Institute Inc., Cary, NC, USA). For SCFA, the effects of diet, time, and their interaction were analyzed using a Linear Mixed Model for repeated measurements:

$$Y_{ijkl}=\mu +\alpha_{i}+\beta_{j}+\alpha \beta_{ij}+\gamma_{k}+\gamma_{l}+\varepsilon_{ijkl}$$

where *Y_ijkl_* is the analyzed variable (SCFA); *µ* is the overall mean; *α_i_* represents the effect of diet (*i* = LR, HR); *β_j_* is time (*j* = 4, 12, 20); *αβ_ij_* is the interaction between diet/nutrient and time; *γ_k_* is the random effect of the block (k = 1, 2, 3, 4); and *γ_l_* is the random component of the individual animal (*l* = 1, 2,…, 30). A heterogeneous autoregressive covariance structure of order 1 was modeled to account for the repeated measurements of time. The residual error component is defined as *ε_ijkl_*. For the analysis of alpha diversity, two similar models were used where (a) *α_i_* represents the effect of diet (*i* = LR, HR); *β_j_* is the sampling depth (*j*), and *αβ_ij_* is the interaction between diet and sampling depth; (b) *α_i_* represents the effect of time (*i* = 4, 12, 20); *β_j_* is the sampling depth (*j*), and *αβ_ij_* is the interaction between time and sampling depth. To account for the repeated measurements of sampling depth, an autoregressive covariance structure was modeled. Values were reported as least squares means (LSMEANS) ± SEM, and statistical significance was set at *p* < 0.05 and *p* < 0.10 are considered trends.

### 4.10. Metabolite Identification

Accurate mass and mass fragmentation patterns of discriminating features were used for compound identification based on queries in the following databases: The Human Metabolome Database (http://www.hmdb.ca/), METLIN (http://metlin.scripps.edu/), and MetFrag database (https://msbi.ipb-halle.de/MetFragBeta/). Annotated features were classified on different levels of identification according to Sumner et al. [66].

## 5. Conclusions

Multi-compartmental metabolomics allowed for the identification of several pathways altered by *ad libitum* intake of a high-fat diet containing fructose compared to one containing HiMaize. Branched-chain keto acids were influenced by fructose intake as lower levels were observed in the plasma metabolome compared to the LR diet, but did not affect circulating BCAA, indicating some specific changes in BCAA metabolism. Irrespective of diet, the time classification revealed a less distinct metabotype at week 12 compared to week 20, indicating a possible adaptive metabolic mechanism operating up to that point. Furthermore, BCAA, glutamine, glutamate, succinate, and several other metabolism products were increased as a time-depended factor during the 5 months dietary intervention and in relation to obesity development and rising levels of glucose and insulin. Overall, the analysis revealed more changes related to amino acid metabolism rather than lipid or carbohydrate metabolism despite the nature of the diets used. This further enhances the importance of some specific amino acids in the development of obesity and responses to high energy diets. The dietary carbohydrate substrate altered significantly specific gut populations, and changes were observed in plasma, urine, and fecal metabolome due to complex interactions at the microbiota-diet-host level. Responses to complex carbohydrates from HiMaize were characterized by known changes in microbiota and SCFA responses in feces and plasma. On the other hand, new and relevant information on fructose digestion and effects on microbiota and multi-compartmental metabolomics were revealed.

## Figures and Tables

**Table 1:** Chemical composition, nutrient intake, and relative energy contribution of the lower-risk (LR) and the high-risk (HR) experimental diets.

	LR	HR		
**Chemical composition (g/kg DM)**				
DM (g/kg, as-fed basis)	917	913		
Ash	63	62		
Protein (N × 6.25)	119	113		
Fat	177	174		
Available carbohydrates	424	555		
**Digestible carbohydrates**				
Available sugars	9	233		
Fructose	0.6	225		
Glucose	1.2	0.8		
Sucrose	7	7		
Starch	415	322		
**Non-digestible carbohydrates**				
Total dietary fiber ^1^	188	100		
Total NSP (soluble NSP)	73 (15)	69 (8)		
RS ^2^	89	2		
AXOS ^3^	3	5		
Fructans	5	6		
Klason lignin	18	18		
**Gross energy (MJ/kg DM)**	20.3	20.7		
**Nutrient intake (g/day) ^5^**			SEM	*p*-value
DM	694	548	78	0.023
Available carbohydrates	295	304	39	0.775
Protein	83	62	9	0.006
Fat	123	95	14	0.015
Total dietary fiber ^1^	130	55	13	<0.0001
**Relative energy contribution (%) ^4^**				
Carbohydrates	41.8	50.8		
Fat	37.9	34.6		
Protein	11.7	10.3		
Total dietary fiber	8.7	4.3		

^1^ Total NSP + fructans + RS + lignin + AXOS; ^2^ RS, resistant starch; determined by enzymatic resistant starch assay (AOAC method 2002.02); ^3^ AXOS, arabinoxylan-oligosaccharides; ^4^ Calculated using the energy conversion factors (FAO) for carbohydrate (17 kJ/g), protein (17 kJ/g), fat (37 kJ/g), and total dietary fiber (8 kJ/g); ^5^ Data presented as LS means; calculated from average feed intake over a 20-week intervention period.

## Data Availability

Mass spectrometry data utilized in this manuscript can be retrieved from the Chorus public repository via: https://chorusproject.org/anonymous/download/experiment/7186696408786408549 (Project name: MERITS WP4 Plasma Metabolomics); https://chorusproject.org/anonymous/download/experiment/6140097670581751756 (Project name: MERITS WP4 Urine Metabolomics); https://chorusproject.org/anonymous/download/experiment/6559124957349905898 (Project name: MERITS WP4 Feces Metabolomics). Gut microbiota 16S RNA sequencing data can be retrieved via the EBI-ENA accession number: ERP124471.

## Supplementary Materials

The following are available online at https://www.mdpi.com/2218-1989/10/11/456/s1, Figure S1: Alpha rarefaction curves and PCoA plots of Weighted UniFrac metrics; differences between collection time points (week 4, week 12, week 20), Figure S2: Score plots of MBPLSR analysis for diet classification models using metabolomics data in positive ionization mode (ESI+), Figure S3: Score plots of MBPLSR analysis for diet classification models using metabolomics data in negative ionization mode (ESI−), Figure S4: Super weights of sMBPLSR analysis for time classification models using metabolomics data in ESI+ and ESI−, Figure S5: sMBPLSR model parameters for a 4-block (metabolome ESI+/ESI−, SCFAs, microbiota) analysis classification of pigs fed LR and HR diets, Figure S6: Correlation loading plot for the variables contributing to the separation of the LR and HR diets using scores of a 4-block model: feces metabolomics in ESI+/ESI−, SCFAs, metataxonomics, Figure S7: Correlation heat map of variables associated with the HR diet intervention, Figure S8: Correlation heat map of variables associated with the LR diet intervention, Figure S9: The representative MRM chromatogram of SCFA standards, Table S1: Short-chain fatty acid measured in feces, Table S2: Short-chain fatty acid measured in fasting plasma, Table S3: sMBPLSR metabolites discriminating between minipigs fed HR and LR diets in plasma, urine, and feces, Table S4: sMBPLSR metabolites discriminating between different collection time points analyzed in plasma, urine, and feces over a five-month dietary intervention period, Table S5: sMBPLSR metabolites discriminating between minipigs fed HR and LR diets in the fecal metabolome, Table S6: Feed ingredients, Table S7: Compound-dependent LC-MS/MS parameters; declustering potential (DP), entrance potential (EP), collision energy (CE), and cell exit potential (CXP).
