# Supplementary material for: Obesity-Related Metabolome and Gut Microbiota Profiles of Juvenile Göttingen Minipigs—Long-Term Intake of Fructose and Resistant Starch
Source: Metabolites. 2020 Nov 12;10(11):456. doi: 10.3390/metabo10110456 (PMC7697781; doi:10.3390/metabo10110456)
Supplement: Supplementary file 1 [file metabolites-10-00456-s001.pdf]

Supplementary materials

Figure S1. Alpha rarefaction curves and PCoA plots of Weighted UniFrac metrics; differences between collection time points (week 4, week 12, week 20)

Figure S2. Score plots of MBPLSR analysis for diet classification models using metabolomics data in positive ionization mode (ESI+)

Figure S3. Score plots of MBPLSR analysis for diet classification models using metabolomics data in negative ionization mode (ESI-)

Figure S4. Super weights of sMBPLSR analysis for time classification models using metabolomics data in ESI+ and ESI-

Figure S5. sMBPLSR model parameters for a 4-block (metabolome ESI+/ESI-, SCFAs, microbiota) analysis classification of pigs fed LR and HR diets

Figure S6. Correlation loading plot for the variables contributing to the separation of the LR and HR diets using scores of a 4-block model: feces metabolomics in ESI+/ESI-, SCFAs, metataxonomics

Figure S7. Correlation heat map of variables associated with the HR diet intervention

Figure S8. Correlation heat map of variables associated with the LR diet intervention

Figure S9. The representative MRM chromatogram of SCFA standards

Table S1. Short-chain fatty acid measured in feces

Table S2. Short-chain fatty acid measured in fasting blood

Table S3. sMBPLSR metabolites discriminating between minipigs fed HR and LR diets in plasma, urine, and feces

Table S4. sMBPLSR metabolites discriminating between different collection time points analyzed in plasma, urine, and feces over a five-month dietary intervention period

Table S5. sMBPLSR metabolites discriminating between minipigs fed HR and LR diets in the fecal metabolome

Table S6. Feed ingredients

Table S7. Compound-dependent LC-MS/MS parameters; declustering potential (DP), entrance potential (EP), collision energy (CE), and cell exit potential (CXP).

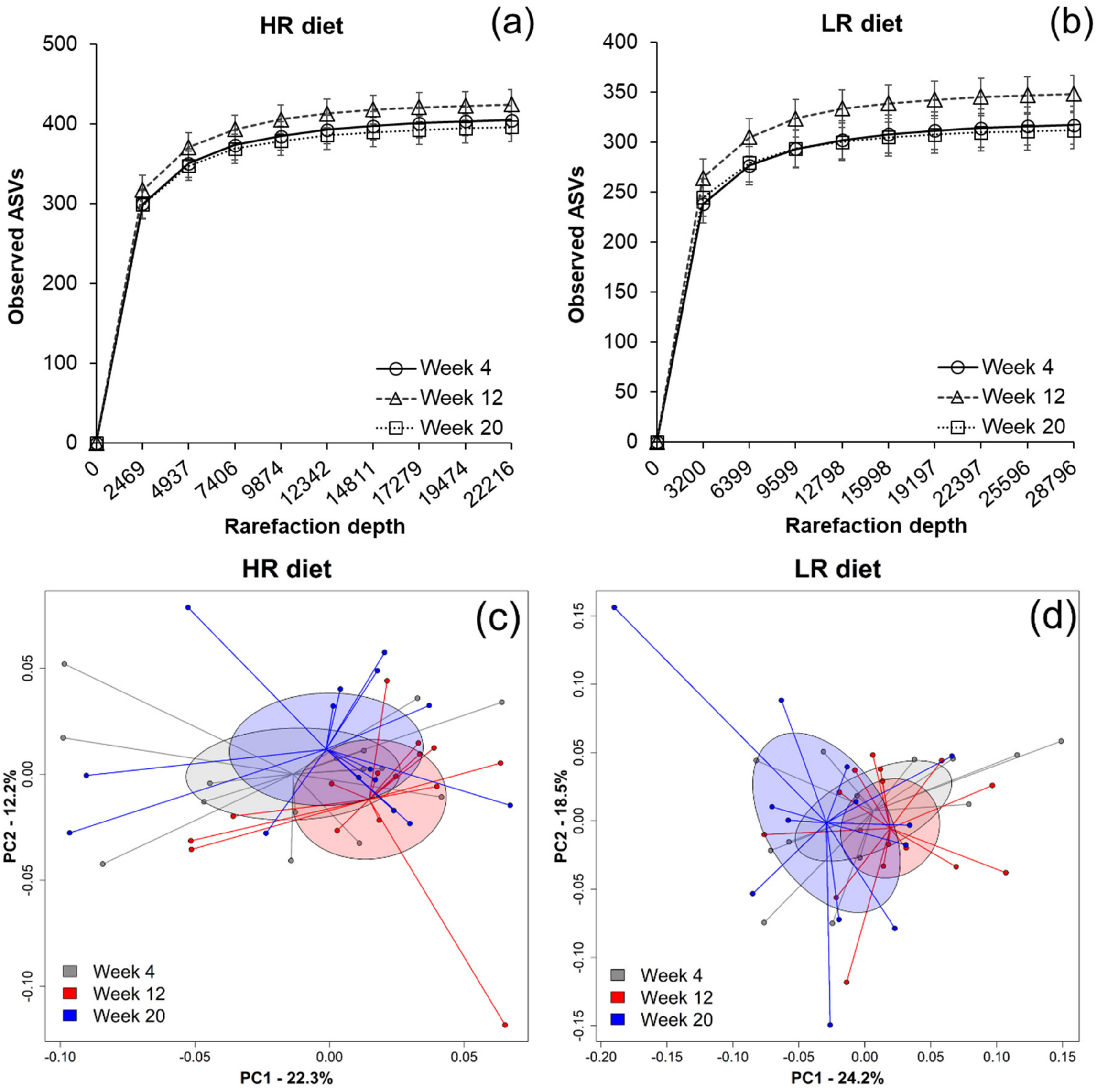

Figure S1. Alpha rarefaction curves (a, b) for each time point (○, week 4; △, week 12; □, week 20) describing the number of observed ASVs (y-axis) as a function of sequencing depth (x-axis); PCoA plots of Weighted UniFrac metrics (c, d) representing individual fecal communities at their respective collection time points (•, week 4; •, week 12; •, week 20). Confidence ellipses are presented in color for each dietary group (99% CI based on standard error); HR, high-risk; LR, lower-risk.

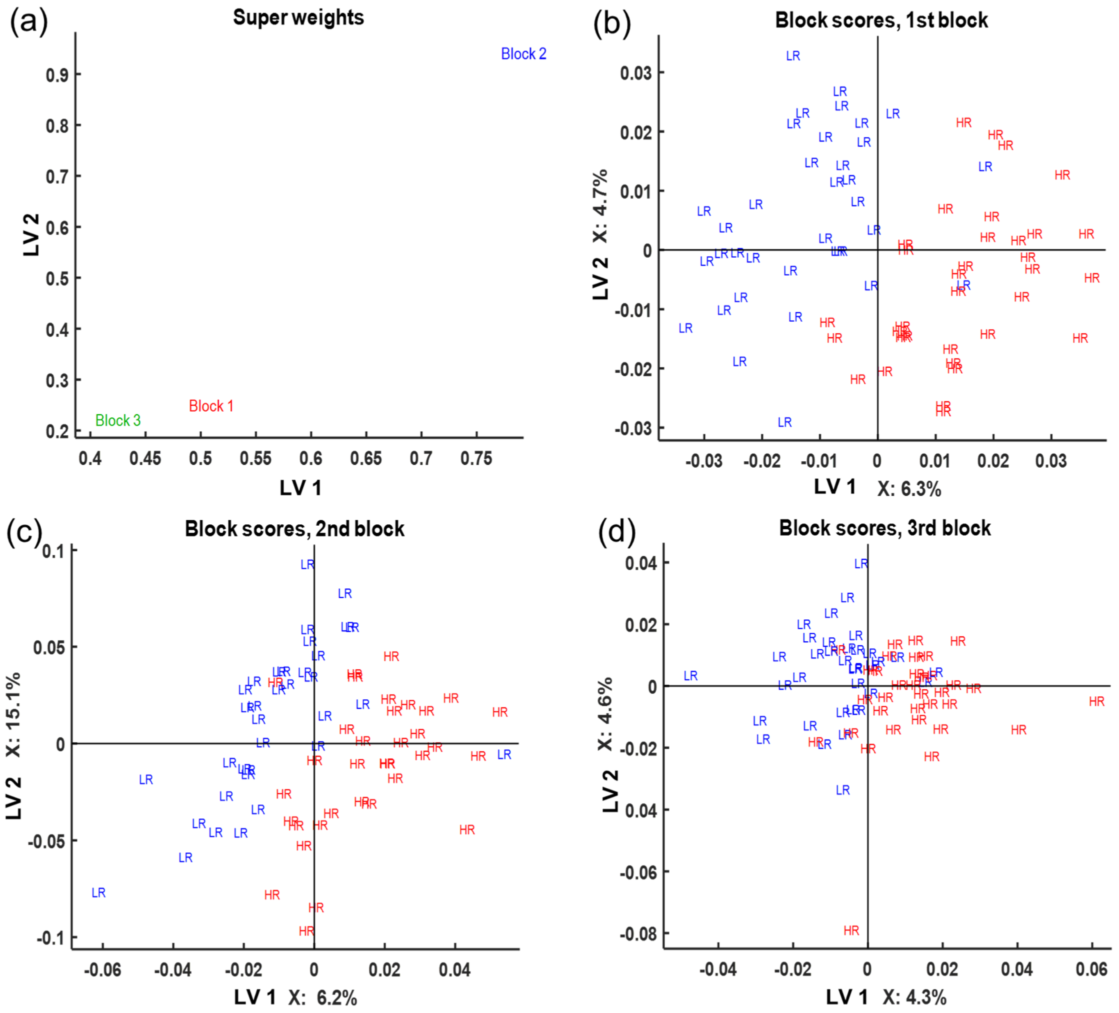

Figure S2. Score plots of sMBPLSR analysis for diet classification models using metabolomics data in positive ionization mode (ESI+): (a) score plot of super weights with block 1 - feces, block 2 - plasma, block 3 - urine; (b, c, d) score plots for each block used in the analysis.

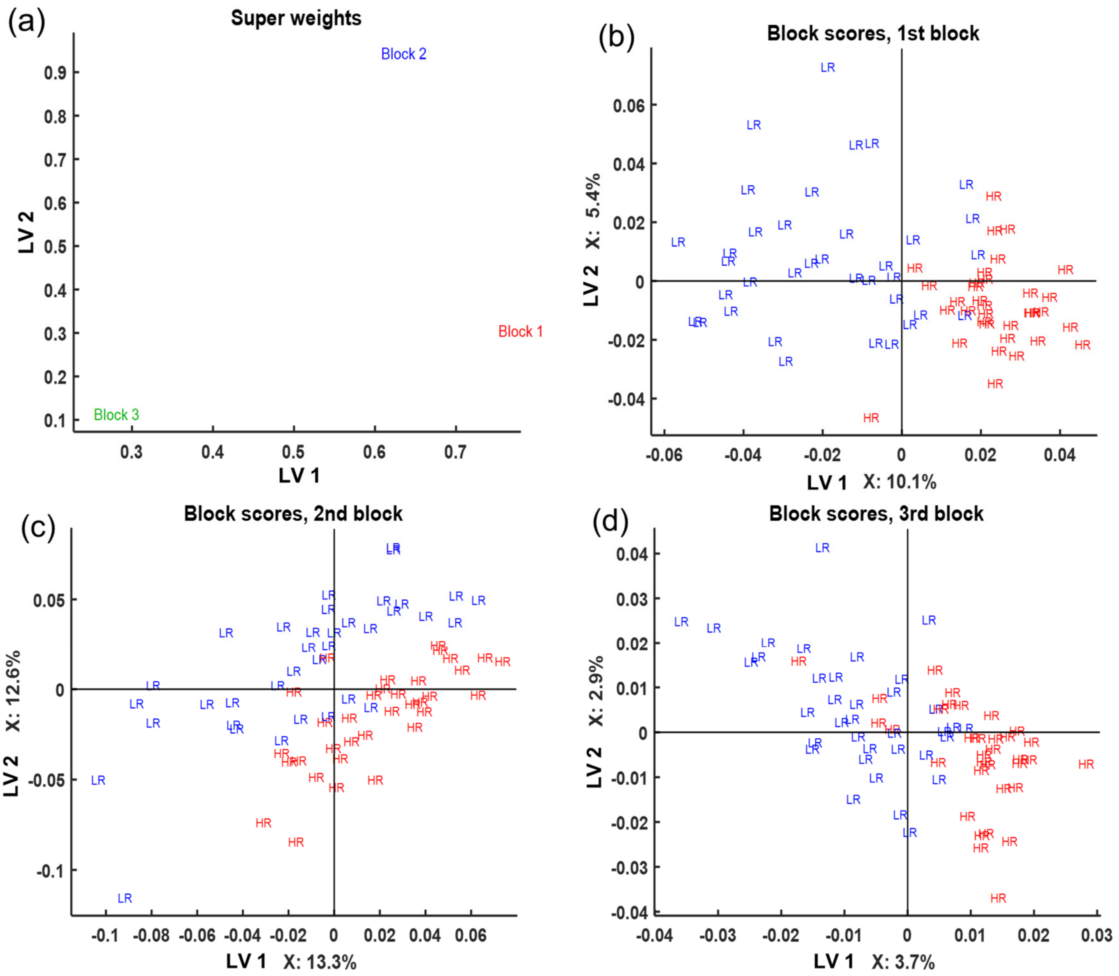

Figure S3. Score plots of sMBPLSR analysis for diet classification models using metabolomics data in negative ionization mode (ESI-): (a) score plot of super weights with block 1 - feces, block 2 - plasma, block 3 - urine; (b, c, d) score plots for each block used in the analysis.

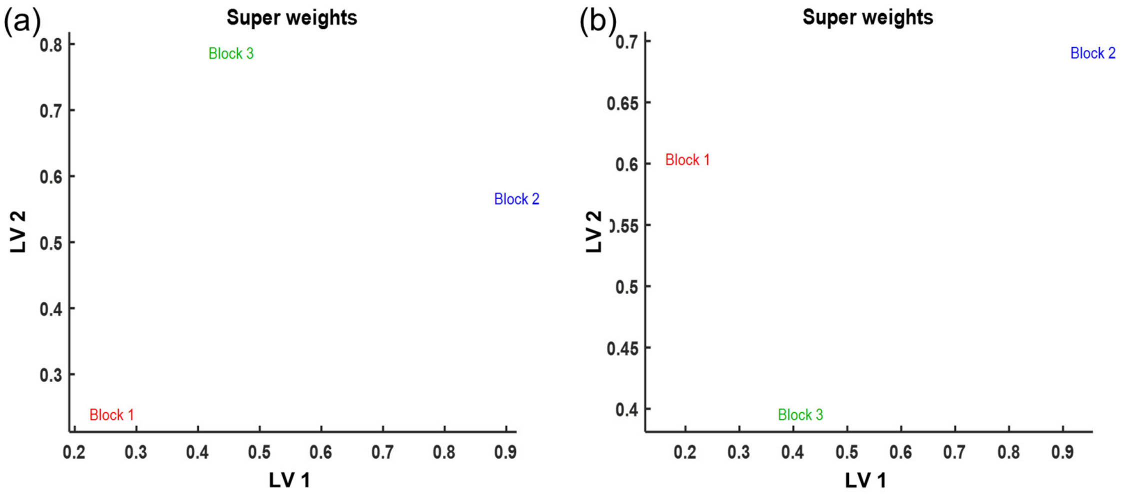

Figure S4. Super weights of sMBPLSR analysis for time classification models using metabolomics data in ESI+ (a) and ESI- (b). Block 1 - feces, block 2 - plasma, block 3 – urine.

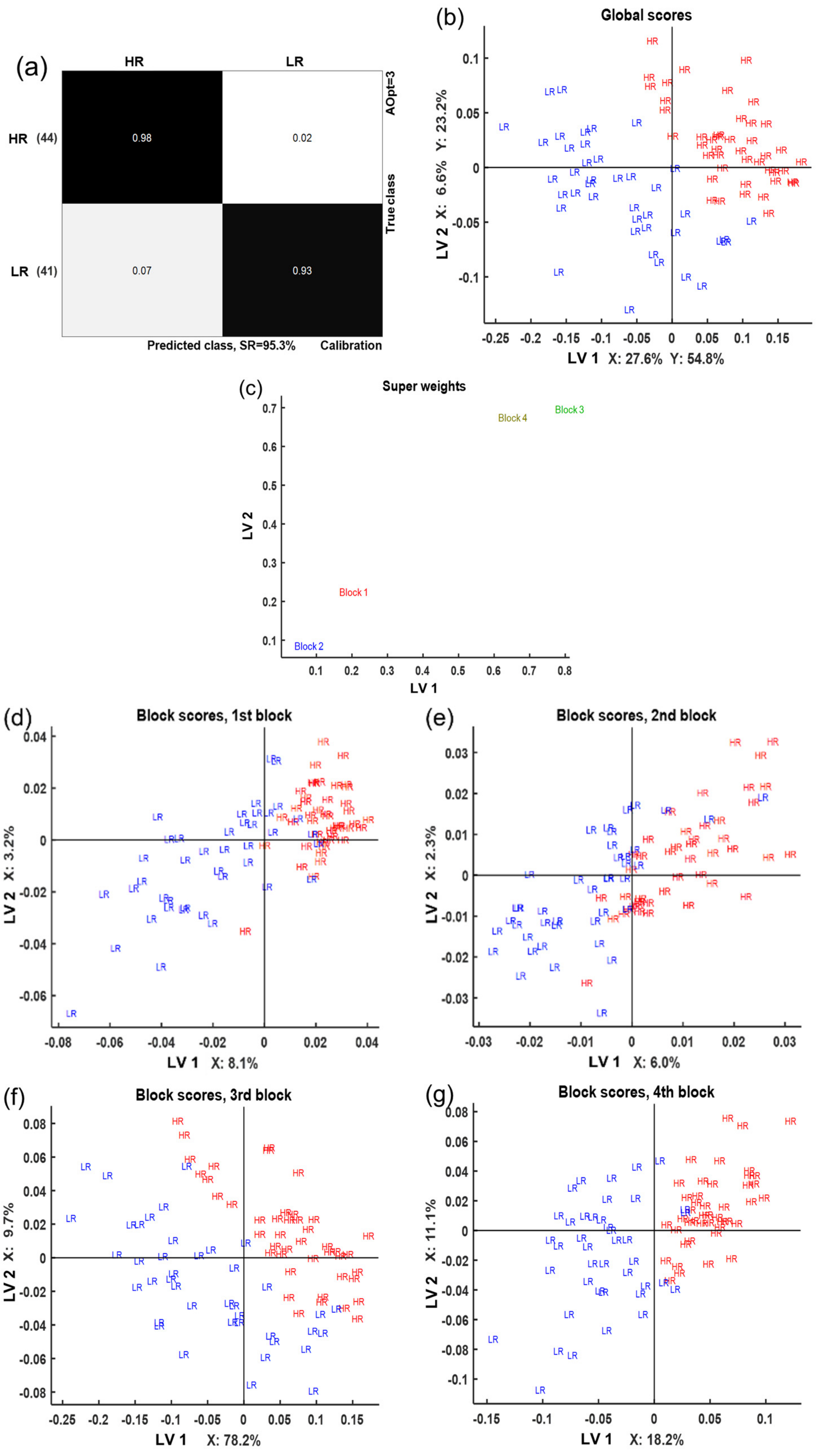

Figure S5. sMBPLSR model parameters for a 4-block (feces metabolome ESI-/ESI+, feces SCFAs, fecal microbiota) analysis classification of pigs fed LR and HR. Confusion matrix (a), global score plot (b), block super weights plot (c) and individual block scores of the fecal metabolome in ESI- (block 1, d), fecal metabolome in ESI+ (block 2, e), SCFAs (block 3, f), microbiota (block 4, g).

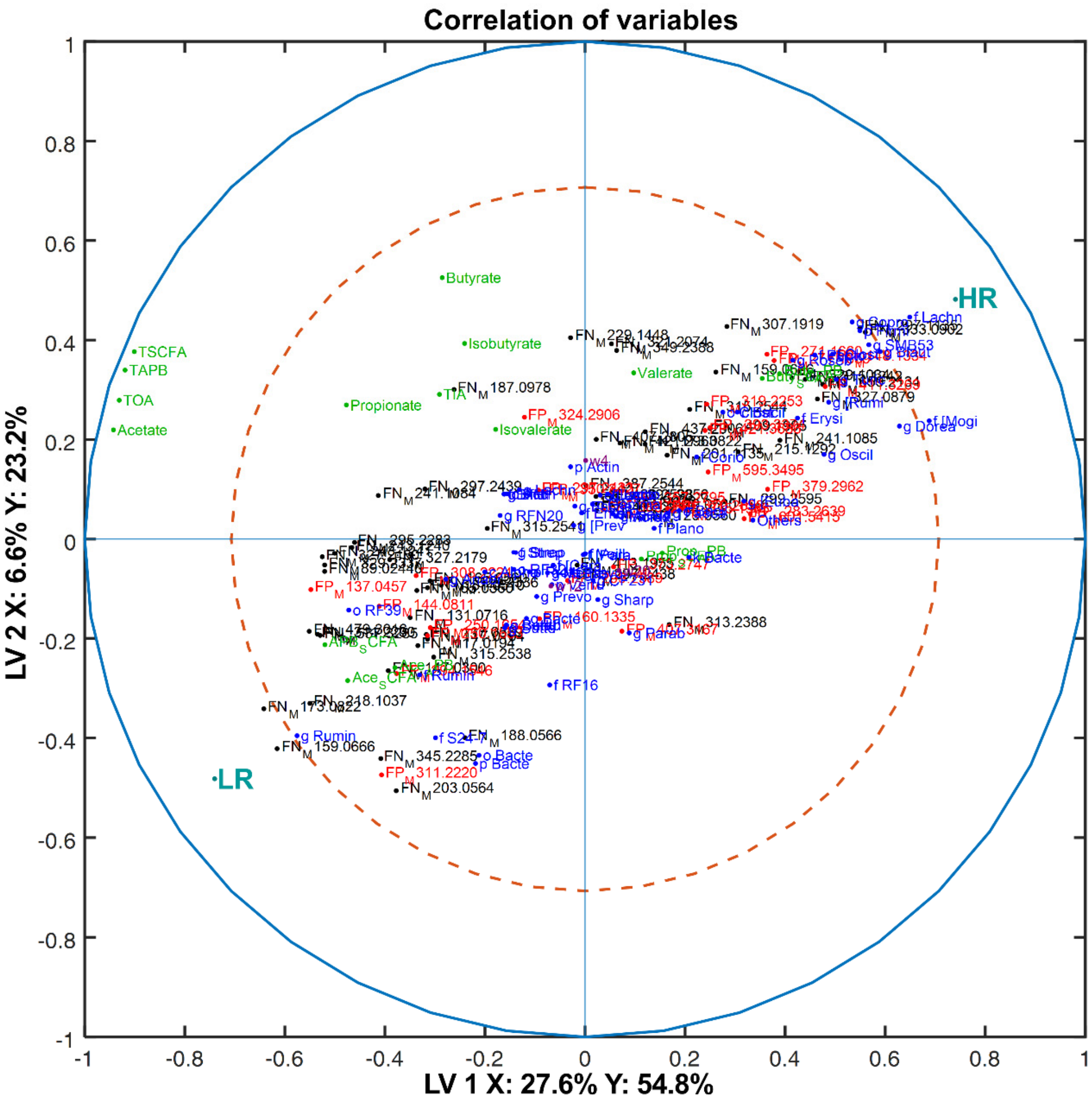

Figure S6. Correlation loading plot for the variables contributing to the separation of the LR and HR diets using scores of a 4-block model: feces metabolomics in ESI+/ESI-, SCFAs, metataxonomics data. The first two components are shown in the plot with the explained variances in sample matrix X and the dietary treatment Y. Variables were colored according to data sets: metataxonomics, blue; SCFAs, green; fecal ESI+ metabolomics, red; fecal ESI- metabolomics, black.

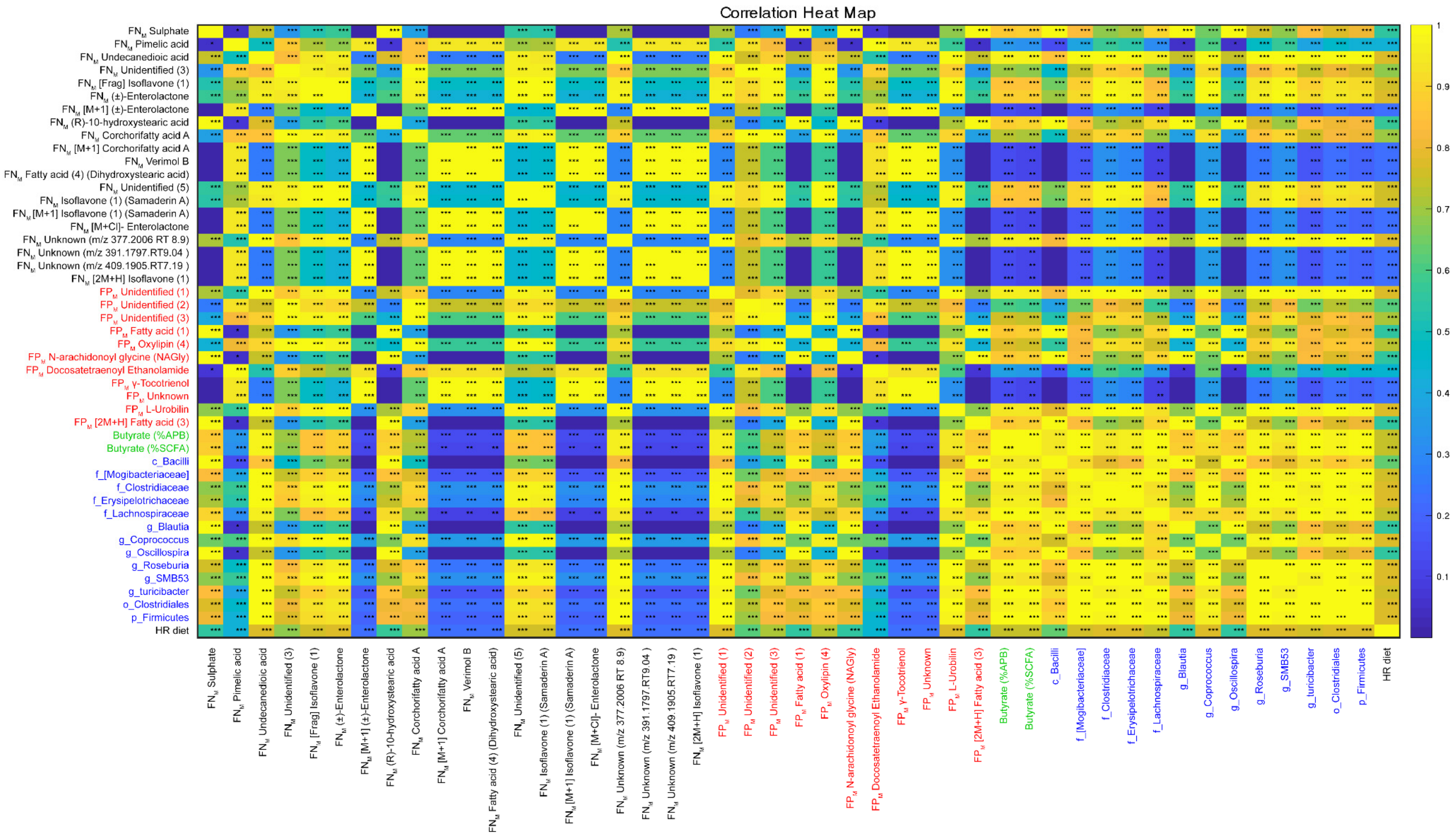

Figure S7. Correlation heat map of variables associated with the HR diet intervention. Variables were colored according to data sets: metataxonomics, blue; SCFAs, green; fecal ESI+ metabolomics, red; fecal ESI- metabolomics, black. Statistical significance value P < 0.05 (*); P < 0.01 (**), P < 0.001 (***).

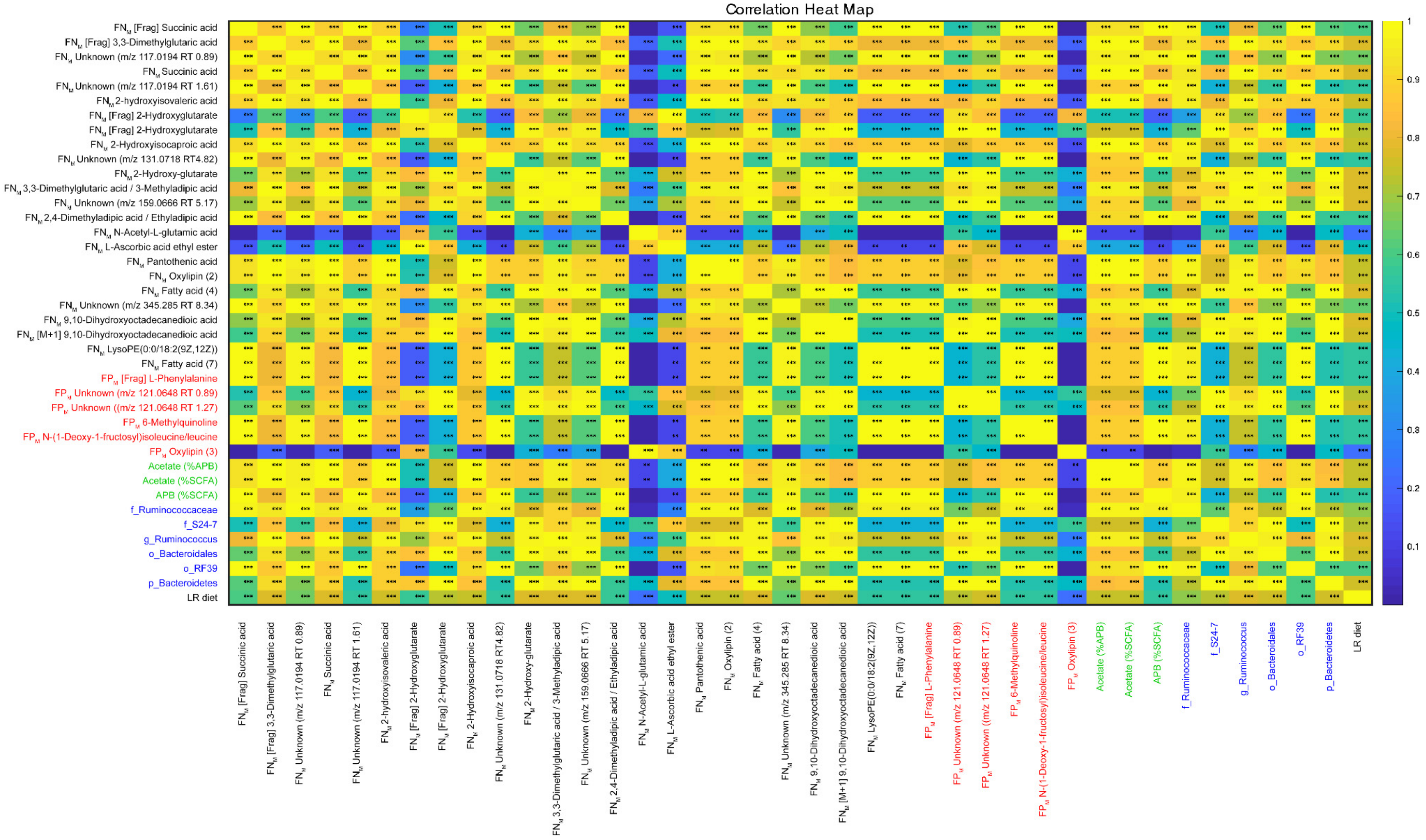

Figure S8. Correlation heat map of variables associated with the LR diet intervention. Variables were colored according to data sets: metataxonomics, blue; SCFAs, green; fecal ESI+ metabolomics, red; fecal ESI- metabolomics, black. Statistical significance value P < 0.05 (*); P < 0.01 (**), P < 0.001 (***).

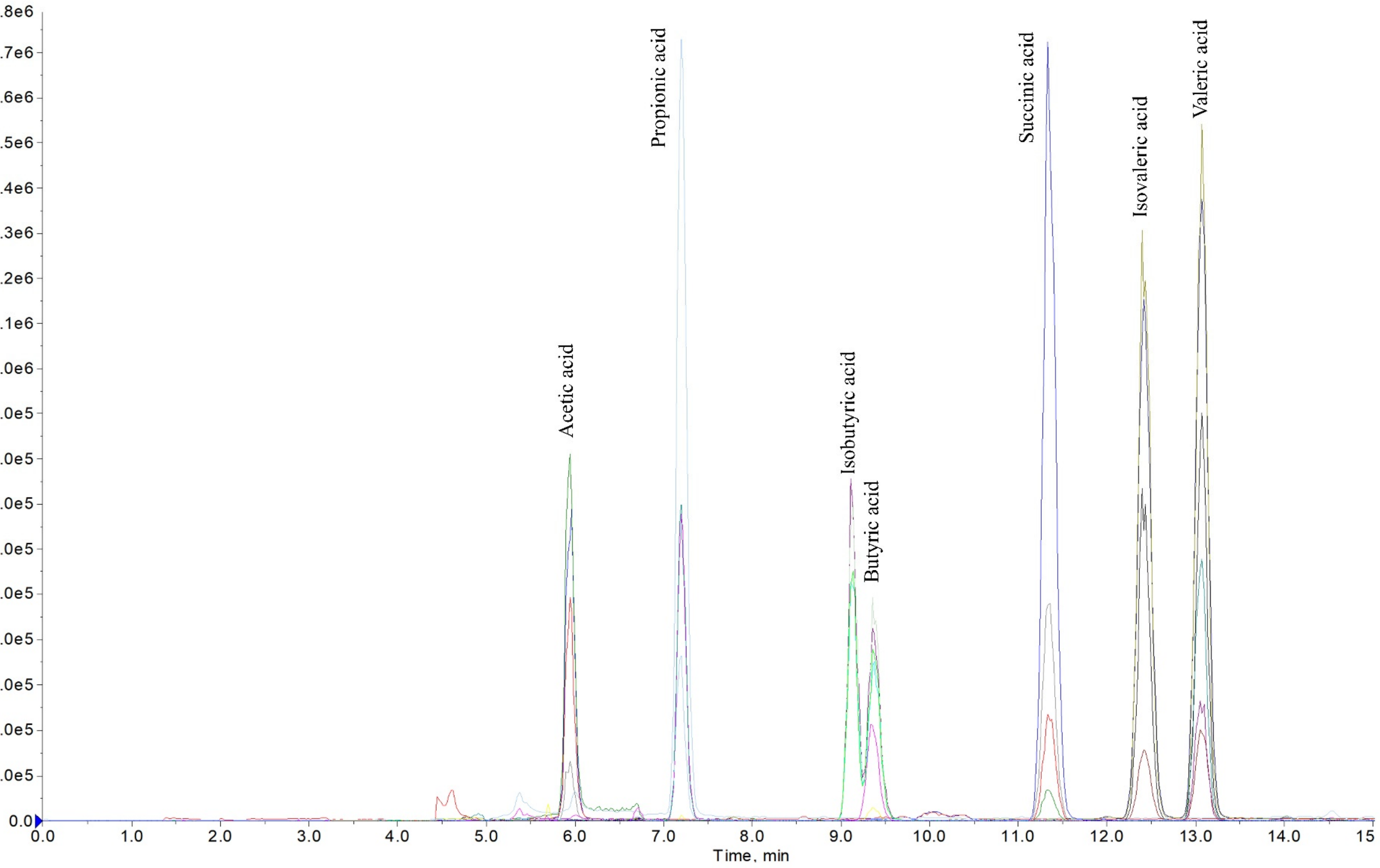

Figure S9. The representative MRM chromatogram of SCFA standards

Table S1. Short-chain fatty acid measured in feces

	HR diet[2]			LR diet[2]			P-value[3]		
	Weeks of intervention			Weeks of intervention			Diet	Week	Week*Diet
Metabolite (mmol/kg)[1]	4	12	20	4	12	20			
Total SCFA	77.2 ± 5.8	67.3 ± 5.8	73.8 ± 6.6	100.1 ± 5.8	100.7 ± 5.6	78.4 ± 7	0.003	0.03	0.02
Total organic acids	78.2 ± 7	67.3 ± 8	74.7 ± 8.8	110.8 ± 7	115.2 ± 7.8	87.9 ± 9	<0.0001	0.08	0.06
Total BCFA	3.4 ± 0.3	3.2 ± 0.3	2.9 ± 0.4	3.1 ± 0.3	3.1 ± 0.3	3.5 ± 0.4	NS	NS	NS
Acetic acid	48.7 ± 5.1	44.3 ± 5.4	45.9 ± 5.3	72 ± 5.1	75.3 ± 5.3	53.8 ± 5.5	<0.0001	0.007	0.007
Propionic acid	8.2 ± 0.8	6.3 ± 0.6	7.2 ± 1.1	9.2 ± 0.8	7.9 ± 0.6	7.4 ± 1.1	NS	0.02	NS
Butyric acid	13.8 ± 1.3	11.1 ± 1.4	14.2 ± 1.6	13.5 ± 1.3	12.6 ± 1.4	11.8 ± 1.6	NS	NS	NS
Valeric acid	2.7 ± 0.3	2.2 ± 0.2	2.4 ± 0.3	2.2 ± 0.3	1.7 ± 0.2	1.7 ± 0.3	0.05	NS	NS
Isovaleric acid	1.5 ± 0.1	1.4 ± 0.1	1.2 ± 0.2	1.2 ± 0.1	1.3 ± 0.1	1.5 ± 0.2	NS	NS	NS
Isobutyric acid	1.8 ± 0.1	1.7 ± 0.1	1.6 ± 0.2	1.7 ± 0.1	1.5 ± 0.1	1.6 ± 0.2	NS	NS	NS
Total APB	71 ± 5.5	61.9 ± 5.4	67.5 ± 6.3	95 ± 5.5	96.1 ± 5.3	73.7 ± 6.7	0.001	0.03	0.02
APB (%TSCFA)	94.7 ± 0.4	95.4 ± 0.4	94.5 ± 0.5	91.9 ± 0.4	91.9 ± 0.4	93 ± 0.5	<0.0001	NS	0.05
Acetate (%TSCFA)	63.9 ± 1.8	66.3 ± 1.7	62.9 ± 2.5	72 ± 1.8	74.8 ± 1.6	69.7 ± 2.6	0.0005	0.03	NS
Propionate (%TSCFA)	10.3 ± 0.6	9.1 ± 0.8	9.4 ± 1	9.1 ± 0.6	8.2 ± 0.7	10 ± 1.1	NS	NS	NS
Butyrate (%TSCFA)	17.8 ± 1.3	17.7 ± 1.4	19 ± 1.6	13.7 ± 1.3	12.5 ± 1.3	14.3 ± 1.6	<0.0001	NS	0.05

[1]Abbreviations: SCFA, short-chain fatty acids; A, acetic acid; P, propionic acid; B, butyric acid; (%TSCFA), proportion in TSCFA; TSCFA, total short-chain fatty acids;

[2]data presented as LSMEANS ± SEM

[3]NS, not significant

Table S2. Short-chain fatty acid measured in fasting plasma

	HR diet[2]			LR diet[2]			P-value[3]		
	Weeks of intervention			Weeks of intervention			Diet	Week	Week*Diet
Metabolite (μM)[1]	4	12	20	4	12	20			
Total SCFA	190.2 ± 14.4	146.4 ± 11.3	140.1 ± 10.5	192.7 ± 14.4	215.8 ± 11.3	185.6 ± 10.2	0.006	0.02	0.009
Total organic acids	215.9 ± 15.6	171.1 ± 12.4	163.5 ± 11.1	222.4 ± 15.6	244.2 ± 12.4	208.7 ± 10.9	0.005	0.006	0.02
Total BCFA	15.9 ± 2.6	10.2 ± 3.1	17.1 ± 3	13.9 ± 2.6	22.2 ± 3.1	25.1 ± 3	0.045	0.03	0.009
Acetic acid	171 ± 13.2	133.7 ± 9.6	120.6 ± 9.3	175.2 ± 13.2	189.7 ± 9.6	156.5 ± 9.1	0.01	0.001	0.020
Propionic acid	1.8 ± 0.2	1.4 ± 0.3	1.6 ± 0.3	2 ± 0.2	2.5 ± 0.3	2.3 ± 0.3	0.007	NS	0.06
Butyric acid	1.6 ± 0.4	1.1 ± 0.3	0.8 ± 0.3	1.7 ± 0.4	1.3 ± 0.3	1.6 ± 0.3	NS	NS	NS
Valeric acid	0.09 ± 0.1	0.08 ± 0.1	0.07 ± 0.1	0.09 ± 0.1	0.11 ± 0.1	0.1 ± 0.1	NS	NS	NS
Succinic acid	25.4 ± 1.9	24.4 ± 1.9	23.3 ± 2.2	29.5 ± 1.9	28.3 ± 1.9	23 ± 2.1	NS	0.05	NS
Isovaleric acid	10.4 ± 2	5.4 ± 2.1	9.8 ± 2.1	7.8 ± 2	13.2 ± 2.1	15.1 ± 2.1	0.08	NS	0.012
Isobutyric acid	5.5 ± 0.8	4.7 ± 1	7.2 ± 1	6.1 ± 0.8	9 ± 1	10 ± 1	0.01	0.0003	0.02
Total APB	174.4 ± 13.5	136.2 ± 9.7	123 ± 9.5	178.9 ± 13.5	193.6 ± 9.7	160.5 ± 9.3	0.01	0.001	0.02
APB (%TSCFA)	91.6 ± 1.4	93.3 ± 1.4	88 ± 2	93.2 ± 1.4	90.2 ± 1.4	86.3 ± 2	NS	0.002	0.04
Acetate (%TSCFA)	89.7 ± 1.4	91.6 ± 1.4	86.4 ± 2.1	91.3 ± 1.4	88.4 ± 1.4	84.1 ± 2.1	NS	0.002	0.04
Propionate (%TSCFA)	0.8 ± 0.2	0.8 ± 0.2	0.5 ± 0.2	0.9 ± 0.2	0.6 ± 0.2	0.9 ± 0.2	NS	NS	0.09
Butyrate (%TSCFA)	0.99 ± 0.1	1 ± 0.2	1.14 ± 0.2	1 ± 0.1	1.2 ± 0.2	1.4 ± 0.2	NS	NS	NS

[1]Abbreviations: SCFA, short-chain fatty acids; A, acetic acid; P, propionic acid; B, butyric acid; (%TSCFA), proportion in TSCFA; TSCFA, total short-chain fatty acids;

[2]data presented as LSMEANS ± SEM

[3]NS, not significant

Table S3. sMBPLSR metabolites discriminating between minipigs fed HR and LR diets in plasma, urine, and feces

LIST NR	IM[1]	MS M/Z[2]	RT[3]	ION	METABOLITE[4]	KEGG[5]	PATHWAY	ID LEVEL	RC HR[6]	RC LR[6]	ANOVA (P-VALUE)
					**PLASMA**						
**1**	POS	102.0552	1.11	[Fragment]	Methionine	C00073	Cysteine and methionine metabolism (map00270)	Level 1	3.22	-3.22	0.000
**2**	POS	102.0916	1.13	[M+H]+	Betaine aldehyde	C00576	Glycine, serine and threonine metabolism (map00260)	Level 2	5.74	-5.74	0.000
**3**	POS	148.0972	1.13	[M+H]+	4-Hydroxyisoleucine	n/a		Level 1	9.23	-9.23	0.000
**4**	POS	194.0818	4.15	[M+H]+	Phenylacetylglycine	C05598	Phenylalanine metabolism (map00360)	Level 1	-0.50	0.50	0.000
**5**	POS	205.0978	3.08	[M+H]+	Tryptophan	C00078	Tryptophan metabolism (map00380)	Level 1	0.25	-0.25	0.076
**6**	POS	357.2800	7.96	[M+H]+	Fatty acid (1) (Tetracosahexaenoic acid / Docosahexaenoic Acid ethyl ester)	n/a	Fatty acids and conjugates metabolism	Level 3	-2.02	2.02	0.000
**7**	POS	391.2855	8.29	[M+H]+	Bile acid (1) (Nutriacholic acid / 12-Ketodeoxycholic acid)	n/a	Bile acid metabolism	Level 3	-0.54	0.54	0.001
**8**	POS	409.1880	3.08	[2M+H]+	Tryptophan	C00078	Tryptophan metabolism (map00380)	Level 1	0.10	-0.10	0.091
**9**	POS	466.3181	6.42	Unknown	Unidentified (4)	n/a		Level 4	0.16	-0.16	0.088
**10**	POS	100.0760	2.33	[M+H]+	δ-Valerolactam	n/a		Level 1	-2.96	2.96	0.752
**11**	POS	100.1125	2.80	[M+H]+	2-Methylpiperidine	n/a		Level 2	0.12	-0.12	0.316
**12**	POS	118.0502	1.02	[M+H]+	Acetylglycine	n/a		Level 1	0.02	-0.02	0.581
**13**	POS	118.0865	0.80	[M+H]+	Betaine	C00719	Glycine, serine and threonine metabolism (map00260)	Level 1	-2.16	2.16	0.283
**14**	POS	132.0770	0.82	[M+H]+	Creatine	C00300	Glycine, serine and threonine metabolism (map00260)	Level 1	-0.95	0.95	0.348
**15**	POS	132.1023	1.44	[M+H]+	Leucine	C00123	Valine, leucine and isoleucine degradation (map00280)	Level 1	-0.09	0.09	0.319
**16**	POS	137.0460	1.10	[M+H]+	Hypoxanthine	C00262	Purine metabolism (map00230)	Level 1	-0.78	0.78	0.480
**17**	POS	153.0663	1.63	[M+H]+	N1-Methyl-2-pyridone-5-carboxamide	C05842	Nicotinate and nicotinamide metabolism (map00760)	Level 2	0.07	-0.07	0.405
**18**	POS	165.0550	1.34	[M+NH4]+	Tyrosine (m/z 182.0809)	n/a		Level 1	-0.92	0.92	0.146
**19**	POS	166.0868	2.35	[M+H]+	Phenylalanine	C00079	Phenylalanine metabolism (map00360)	Level 1	0.73	-0.73	0.655
**20**	POS	180.0659	3.79	[M+H]+	Hippuric acid	C01586	Phenylalanine metabolism (map00360)	Level 1	-0.02	0.02	0.130
**21**	POS	182.0817	1.34	[M+H]+	Tyrosine	C00082	Tyrosine metabolism (map00350)	Level 1	-0.99	0.99	0.170
**22**	POS	204.1236	1.03	[M+H]+	Acetylcarnitine	C02571	Insulin resistance (map04931)	Level 1	-0.13	0.13	0.156
**23**	POS	259.0996	2.62	Unknown	Unidentified (1)	n/a		Level 4	0.03	-0.03	0.916
**24**	POS	273.1152	3.12	Unknown	Unidentified (2)	n/a		Level 4	0.28	-0.28	0.331

**25**	POS	387.1814	8.00	Unknown	Unidentified (3)	n/a		Level 3	0.07	-0.07	0.718
**26**	POS	542.3257	8.84	[M+H]+	LysoPC(20:5)	n/a	Fatty acids and conjugates metabolism	Level 2	0.24	-0.24	0.817
**27**	NEG	103.0401	1.79	[M-H]-	2-Hydroxybutyrate	C05984	Propanoate metabolism (map00640)	Level 1	-3.01	3.01	0.000
**28**	NEG	115.0402	2.29	[M-H]-	α-ketoisovaleric acid	C00141	Valine, leucine and isoleucine degradation (map00280)	Level 1	0.41	-0.41	0.008
**29**	NEG	116.0354	1.05	[M-H]-	L-2-Amino-3-oxobutanoic acid / N-Acetylglycine	C03508	Glycine, serine and threonine metabolism (map00260)	Level 2	-1.53	1.53	0.011
**30**	NEG	117.0558	2.99	[M-H]-	3-Hydroxyisovalerate	C20827	Valine, leucine and isoleucine degradation (map00280)	Level 2	-0.05	0.05	0.095
**31**	NEG	128.0354	1.20	[M-H]-	Pyroglutamic acid, N-Acryloylglycine / 4-Oxoproline	C02237	D-Glutamine and D-glutamate metabolism (map00471)	Level 2	-0.32	0.32	0.076
**32**	NEG	129.0559	3.91	[M-H]-	Ketoleucine	C00233	Valine, leucine and isoleucine degradation (map00280)	Level 1	-1.93	1.93	0.008
**33**	NEG	129.0559	3.56	[M-H]-	Ketoisoleucine (3-Methyl-2-oxovaleric acid)	C00671	Valine, leucine and isoleucine degradation (map00280)	Level 1	-1.17	1.17	0.022
**34**	NEG	178.0512	3.80	[M-H]-	Hippuric acid	C01586	Phenylalanine metabolism (map00360)	Level 1	-1.11	1.11	0.068
**35**	NEG	187.0074	4.47	[M-H]-	p-Cresol sulfate	n/a		Level 1	-0.76	0.76	0.006
**36**	NEG	188.0719	6.16	[M-H]-	3-Indolepropionic acid	n/a		Level 2	-0.41	0.41	0.000
**37**	NEG	192.0668	4.17	[M-H]-	Phenylacetylglycine	C05598	Phenylalanine metabolism (map00360)	Level 1	-3.48	3.48	0.000
**38**	NEG	203.0828	3.10	[M-H]-	Tryptophan	C00078	Tryptophan metabolism (map00380)	Level 1	-0.14	0.14	0.088
**39**	NEG	212.0025	3.89	[M-H]-	Indoxylsulfuric acid	n/a		Level 1	1.66	-1.66	0.010
**40**	NEG	218.1035	2.53	[M-H]-	Pantothenic Acid	C00864	Pantothenate and CoA biosynthesis (map00770)	Level 1	0.58	-0.58	0.016
**41**	NEG	230.9970	4.41	[M-H]-	Vanillin 4-sulfate	n/a		Level 2	4.81	-4.81	0.003
**42**	NEG	391.2856	7.99	[M-H]-	Ursodeoxycholic acid	C07880	Secondary bile acid biosynthesis (map00121)	Level 1	-1.06	1.06	0.012
**43**	NEG	437.2910	7.99	[M+FA]-	Ursodeoxycholic acid	C07880	Secondary bile acid biosynthesis (map00121)	Level 1	-3.49	3.49	0.000
**44**	NEG	464.3018	6.45	[M-H]-	Bile acid (1) (Glycocholic acid / 3a,7b,12a-Trihydroxyoxocholanyl-Glycine)	n/a	Bile acid metabolism	Level 3	2.80	-2.80	0.098
**45**	NEG	89.0245	1.13	[M-H]-	Lactic acid	C00256	Pyruvate metabolism (map00620)	Level 2	-0.24	0.24	0.356
**46**	NEG	130.0875	1.46	[M-H]-	Leucine	C00123	Valine, leucine and isoleucine degradation (map00280)	Level 1	-0.41	0.41	0.199
**47**	NEG	135.0313	1.12	[M-H]-	Hypoxanthine	C00262	Purine metabolism (map00230)	Level 1	0.22	-0.22	0.485
**48**	NEG	145.0144	1.08	[M-H]-	Ketoglutaric acid (oxoglutaric acid)	C00026	D-Glutamine and D-glutamate metabolism (map00471)	Level 1	0.15	-0.15	0.301
**49**	NEG	146.0459	0.82	[M-H]-	Glutamate	C00025	D-Glutamine and D-glutamate metabolism (map00471)	Level 1	-0.21	0.21	0.260
**50**	NEG	164.0719	2.34	[M-H]-	Phenylalanine	C00079	Phenylalanine metabolism (map00360)	Level 1	0.20	-0.20	0.806
**51**	NEG	180.0667	1.35	[M-H]-	Tyrosine	C00082	Tyrosine metabolism (map00350)	Level 1	0.41	-0.41	0.121
**52**	NEG	187.0421	1.12	[M-H]-	1-Hydroxy-2-naphthoic acid	n/a		Level 2	0.40	-0.40	0.559

**53**	NEG	189.0406	1.36	[M-H]-	3-Dehydroquinic acid / 2-Keto-5-epi-valiolone / 4-Hydroxy-4-methyl-2-oxoadipate	n/a		Level 2	0.59	-0.59	0.741
**54**	NEG	201.0381	1.13	Unknown	Unidentified (1)	n/a		Level 4	0.81	-0.81	0.278
**55**	NEG	215.0328	0.81	[M+Cl]-	Fructose	C02336	Amino sugar and nucleotide sugar metabolism (map00520)	Level 1	1.19	-1.19	0.978
**56**	NEG	231.9676	1.13	Unknown	Unidentified (2)	n/a		Level 4	0.71	-0.71	0.281
**57**	NEG	245.0491	5.51	[M+SO3H]-	Unidentified (3) (m/z 165.0929)	n/a		Level 4	-2.56	2.56	0.774
**58**	NEG	249.0229	6.26	[M-H]-	4,4'-Sulfonyldiphenol	n/a		Level 2	-1.14	1.14	0.939
**59**	NEG	255.9599	1.44	[M-H]-	5,7-dichloro-kynurenic acid	n/a		Level 2	-0.08	0.08	0.104
**60**	NEG	267.0735	1.54	[M-H]-	Inosine	C00294	Purine metabolism (map00230)	Level 1	1.48	-1.48	0.110
**61**	NEG	283.0825	4.30	[M-H]-	p-Cresol glucuronide	n/a		Level 2	-1.58	1.58	0.184
**62**	NEG	446.2911	7.08	[M-H2O-H]-	3a,7b,12a-Trihydroxyoxocholanyl-Glycine (m/z 465.3097)	n/a	Bile acid metabolism	Level 2	0.03	-0.03	0.583
**63**	NEG	448.3068	6.87	[M-H]-	Glycoursodeoxycholic acid	n/a	Bile acid metabolism	Level 1	-0.14	0.14	0.788
**64**	NEG	453.2857	7.45	[M+FA]-	Hyocholic acid	n/a	Bile acid metabolism	Level 1	0.87	-0.87	0.165
**65**	NEG	498.2895	6.16	[M-H]-	Bile acid (2) (Tauroursodeoxycholic acid / Taurochenodesoxycholic acid / Taurodeoxycholic acid)	n/a	Bile acid metabolism	Level 3	1.14	-1.14	0.825
**66**	NEG	514.2841	5.84	[M-H]-	Bile acid (3) (Tauroursocholic acid / Taurohyocholic acid)	n/a	Bile acid metabolism	Level 3	0.13	-0.13	0.215
						**URINE**					
**1**	POS	100.0747	2.07	[M+H]+	δ-Valerolactam	n/a		Level 1	0.76	-0.76	0.001
**2**	POS	116.0694	1.19	[M+H]+	Acetamidopropanal	C18170		Level 2	0.22	-0.22	0.000
**3**	POS	128.0178	0.58	[M+H]+	2-Acetylthiazole	n/a		Level 2	-0.04	0.04	0.000
**4**	POS	134.0586	3.48	[M+H]+	6-Hydroxyindole	n/a		Level 2	0.23	-0.23	0.000
**5**	POS	146.1161	0.91	[M+H]+	N,N-dimethyl-L-Valine	n/a		Level 1	-0.37	0.37	0.000
**6**	POS	150.0534	2.75	[M+H]+	5,6-Dihydroxyindole	C05578	Tyrosine metabolism (map00350)	Level 2	0.18	-0.18	0.000
**7**	POS	160.0952	3.04	[M+H]+	Acetyl-DL-Valine	n/a		Level 1	-0.04	0.04	0.033
**8**	POS	170.0582	4.76	Unknown	Unidentified (1)	n/a		Level 4	-2.83	2.83	0.000
**9**	POS	170.0583	5.37	Unknown	Unidentified (2)	n/a		Level 4	-0.47	0.47	0.000
**10**	POS	172.0950	3.76	[M+H]+	N-butanoyl-lhomoserine lactone	n/a		Level 2	-0.51	0.51	0.000
**11**	POS	229.1523	0.95	[M+H]+	Isoleucylproline / Leucylproline	n/a		Level 2	0.64	-0.64	0.003
**12**	POS	237.0845	2.95	[M+H]+	Formylkynurenine	C02700	Tryptophan metabolism (map00380)	Level 2	-0.41	0.41	0.015

**13**	POS	245.0894	4.77	Unknown	Unidentified (4)	n/a		Level 4	-1.70	1.70	0.000
**14**	POS	399.2088	1.94	Unknown	Unidentified (6)	n/a		Level 4	2.70	-2.70	0.070
**15**	POS	399.3547	9.22	Unknown	Unidentified (7)	n/a		Level 4	0.33	-0.33	0.094
**16**	POS	404.1510	3.55	Unknown	Unidentified (8)	n/a		Level 4	0.04	-0.04	0.009
**17**	POS	413.1334	2.29	Unknown	Glucuronated Unidentified (9)	n/a		Level 4	-3.36	3.36	0.020
**18**	POS	430.1291	2.66	Unknown	Unidentified (10)	n/a		Level 4	-0.08	0.08	0.001
**19**	POS	443.1439	2.57	Unknown	Glucuronated Unidentified (12)	n/a		Level 4	0.03	-0.03	0.063
**20**	POS	479.1525	4.09	[M+H]+	4'-O-methyl-(-)-epicatechin-5-O-beta-glucuronide	n/a		Level 2	-1.80	1.80	0.014
**21**	POS	504.2545	3.71	Unknown	Glucuronated Unidentified (13)	n/a		Level 4	-1.63	1.63	0.022
**22**	POS	114.0649	0.74	[M+H]+	Creatinine	C00791	Arginine and proline metabolism (map00330)	Level 1	0.06	-0.06	0.110
**23**	POS	118.0849	0.72	[M+H]+	Betaine	C00719	Glycine, serine and threonine metabolism (map00260)	Level 1	0.65	-0.65	0.360
**24**	POS	118.1213	0.77	[M+H]+	2-Diethylaminoethanol	n/a		Level 2	-0.35	0.35	0.275
**25**	POS	132.0752	0.74	[M+H]+	Creatine	C00300	Glycine, serine and threonine metabolism (map00260)	Level 1	0.01	-0.01	0.319
**26**	POS	134.1161	0.87	[M+H]+	Bis (2-hydroxypropyl) amine	n/a		Level 2	0.06	-0.06	0.308
**27**	POS	137.0443	0.94	[M+H]+	Hypoxanthine	C00262	Purine metabolism (map00230)	Level 1	0.54	-0.54	0.852
**28**	POS	152.0550	0.81	[M+H]+	Guanine	C00242	Purine metabolism (map00230)	Level 1	0.02	-0.02	0.498
**29**	POS	153.0643	1.42	[M+H]+	N-Methyl-2-pyridone-5-carboxamide (Nudifloramide)	n/a		Level 1	0.22	-0.22	0.235
**30**	POS	180.0635	3.54	[M+H]+	Hippuric acid	C01586	Phenylalanine metabolism (map00360)	Level 1	-0.10	0.10	0.311
**31**	POS	194.0791	3.90	[M+H]+	Phenylacetylglycine	C05598	Phenylalanine metabolism (map00360)	Level 1	-0.81	0.81	0.118
**32**	POS	224.0717	2.26	Unknown	Unidentified (3)	n/a		Level 4	0.13	-0.13	0.328
**33**	POS	259.0963	2.43	[M+H]+	Yangonin	C09980	Biosynthesis of phenylpropanoids (map01061)	Level 2	0.34	-0.34	0.164
**34**	POS	283.1722	2.92	[M+H]+	Hexaethylene glycol	n/a		Level 2	0.32	-0.32	0.151
**35**	POS	327.1975	3.15	[M+H]+	Heptaethylene glycol	n/a		Level 2	0.06	-0.06	0.172
**36**	POS	344.2241	3.15	[M+NH4]+	Heptaethylene glycol	n/a		Level 2	0.49	-0.49	0.118
**37**	POS	388.2501	3.35	[M+H]+	Octaethylene glycol	n/a		Level 2	0.67	-0.67	0.185
**38**	POS	432.2760	3.52	Unknown	Unidentified (11)	n/a		Level 4	0.19	-0.19	0.265
**39**	NEG	158.0823	3.04	[M-H]-	2-Methylbutyrylglycine / Isovalerylglycine / Valerylglycine	n/a		Level 2	-0.37	0.37	0.035
**40**	NEG	181.0507	2.93	[M-H]-	Hydroxyphenyllactic acid	C03672	Tyrosine metabolism (map00350)	Level 1	0.12	-0.12	0.002
**41**	NEG	189.0404	1.17	[M-H]-	3-Dehydroquinate	C00944	Phenylalanine, tyrosine and tryptophan biosynthesis (map00400)	Level 2	0.19	-0.19	0.019

**42**	NEG	191.0196	0.84	[M-H]-	Isocitrate	C00311	Citrate cycle (TCA cycle) (map00020)	Level 1	-2.63	2.63	0.000
**43**	NEG	191.0196	0.96	[M-H]-	Citrate	C00158	Citrate cycle (TCA cycle) (map00020)	Level 1	-2.06	2.06	0.009
**44**	NEG	192.0666	3.90	[M-H]-	Phenylacetylglycine	C05598	Phenylalanine metabolism (map00360)	Level 1	-2.43	2.43	0.083
**45**	NEG	201.1132	5.52	[M-H]-	Sebacic acid	C08277	Fatty acids and conjugates metabolism	Level 1	-0.09	0.09	0.028
**46**	NEG	212.0023	3.58	[M-H]-	Indoxylsulfuric acid	n/a		Level 1	0.57	-0.57	0.002
**47**	NEG	218.1034	2.32	[M-H]-	Pantothenic Acid	C00864	Pantothenate and CoA biosynthesis (map00770)	Level 1	0.74	-0.74	0.044
**48**	NEG	227.1289	6.35	[M-H]-	Traumatic acid ((2E)-Dodecenedioic acid)	C16308	alpha-Linolenic acid metabolism (map00592)	Level 2	-0.18	0.18	0.004
**49**	NEG	229.1446	6.75	[M-H]-	Dodecanedioic acid	C02678	Fatty acids and conjugates metabolism	Level 2	-0.04	0.04	0.006
**50**	NEG	235.0724	2.96	[M-H]-	Formylkynurenine	C02700	Tryptophan metabolism (map00380)	Level 2	-0.36	0.36	0.013
**51**	NEG	243.0774	4.76	[M-H]-	Indolylacryloylglycine	n/a		Level 2	-2.17	2.17	0.000
**52**	NEG	289.1656	4.57	[M-H]-	1-Octen-3-yl glucoside	n/a		Level 2	0.10	-0.10	0.042
**53**	NEG	293.1759	7.57	[M-H]-	Unidentified (2)	n/a		Level 4	-2.33	2.33	0.077
**54**	NEG	308.0776	3.47	[M-H]-	Indoxyl glucuronide	n/a		Level 2	1.61	-1.61	0.000
**55**	NEG	324.0724	2.75	[M-H]-	Dihydroxy-1H-indole glucuronide I	n/a		Level 2	2.43	-2.43	0.000
**56**	NEG	135.0311	0.94	[M-H]-	Hypoxanthine	C00262	Purine metabolism (map00230)	Level 1	0.09	-0.09	0.287
**57**	NEG	178.0509	3.54	[M-H]-	Hippuric acid	C01586	Phenylalanine metabolism (map00360)	Level 1	0.11	-0.11	0.376
**58**	NEG	187.0975	4.87	[M-H]-	Azelaic acid	C08261	Fatty acids and conjugates metabolism	Level 1	-1.45	1.45	0.365
**59**	NEG	194.0459	2.89	[M-H]-	Alpha-Hydroxyhippuric acid / 4-Carboxyphenylglycine / 4-Hydroxyhippuric acid	n/a		Level 2	-0.54	0.54	0.167
**60**	NEG	199.0975	5.20	[M-H]-	cis-4-Decenedioic acid	n/a	Fatty acids and conjugates metabolism	Level 2	-0.06	0.06	0.450
**61**	NEG	231.9921	3.00	[M+SO3H]-	3-Hydroxyanthranilic acid (m/z 152.0354)	n/a		Level 2	0.84	-0.84	0.651
**62**	NEG	255.0510	2.48	[M-H]-	Piscidic acid	n/a		Level 2	0.49	-0.49	0.957
**63**	NEG	261.0435	3.36	Unknown	Unidentified (1)	n/a		Level 4	0.19	-0.19	0.324
**64**	NEG	269.0666	3.27	[M-H]-	Phenyl glucuronide	n/a		Level 1	-1.80	1.80	0.461
**65**	NEG	283.0821	4.04	[M-H]-	p-Cresol glucuronide	n/a		Level 2	-0.48	0.48	0.745
**66**	NEG	345.0978	5.52	Unknown	Glucuronide Unidentified (3) (m/z 169.0659)	n/a		Level 4	0.40	-0.40	0.719
**67**	NEG	363.1657	4.73	Unknown	Unidentified (4)	n/a		Level 4	0.01	-0.01	0.248
**68**	NEG	387.2863	7.06	[M+FA]-	Unidentified (5) (m/z 343.2969)	n/a		Level 4	-0.22	0.22	0.152
**69**	NEG	414.0958	5.06	Unknown	Unidentified (6)	n/a		Level 4	0.04	-0.04	0.356
**70**	NEG	433.2074	5.29	Unknown	Unidentified (7)	n/a		Level 4	0.30	-0.30	0.311

**71**	NEG	507.2231	5.45	Unknown	Glucuronide Unidentified (8) (m/z 331.1903)	n/a		Level 4	-0.16	0.16	0.377
**72**	NEG	567.1713	4.03	[2M+H]-	p-Cresol glucuronide	n/a		Level 2	-0.09	0.09	0.698
						**FECES**					
**1**	POS	124.0394	0.88	[M+H]+	Niacin (Nicotinic acid)	C00253	Nicotinate and nicotinamide metabolism (map00760)	Level 1	-0.58	0.58	0.000
**2**	POS	130.0499	0.72	[M+H]+	Pyrrolidone-5-carboxylic acid (Pyroglutamic acid)	C02237	D-Glutamine and D-glutamate metabolism (map00471)	Level 2	-0.36	0.36	0.002
**3**	POS	130.1227	0.75	[M+H]+	L-Pipecolic acid	C00408	Lysine degradation (map00310)	Level 1	0.46	-0.46	0.000
**4**	POS	132.1020	0.91	[M+H]+	N,N-Diethylglycine / beta-Alaninebetaine	n/a		Level 2	0.22	-0.22	0.062
**5**	POS	137.0457	0.89	[M+H]+	Hypoxanthine	C00262	Purine metabolism (map00230)	Level 1	-0.92	0.92	0.000
**6**	POS	144.0811	3.56	[M+H]+	Quinaldine	n/a		Level 2	-0.60	0.60	0.000
**7**	POS	148.1334	0.72	Unknown	Unidentified (1)	n/a		Level 4	2.87	-2.87	0.000
**8**	POS	153.0407	0.90	[M+H]+	Xanthine	C00385	Purine metabolism (map00230)	Level 1	-0.29	0.29	0.000
**9**	POS	160.1335	0.79	[M+H]+	Methacholine / Propionylcholine	C07471		Level 2	-0.18	0.18	0.000
**10**	POS	166.0867	2.15	[M+H]+	Phenylalanine	C00079	Phenylalanine metabolism (map00360)	Level 1	0.08	-0.08	0.003
**11**	POS	188.1761	0.65	[M+H]+	N1-Acetylspermidine	C00612		Level 2	-0.26	0.26	0.000
**12**	POS	220.1185	2.39	[M+H]+	Pantothenic Acid	C00864	Pantothenate and CoA biosynthesis (map00770)	Level 1	-1.01	1.01	0.000
**13**	POS	231.1134	4.26	[M+H]+	Tetrahydro-1-methyl-beta-carboline-3-carboxylic acid (TRP condensation)	n/a		Level 2	0.35	-0.35	0.000
**14**	POS	256.2645	15.92	[M+H]+	Palmitic amide	n/a	Fatty acids and conjugates metabolism	Level 2	0.47	-0.47	0.015
**15**	POS	271.1659	3.50	Unknown	Unidentified (3)	n/a		Level 4	1.57	-1.57	0.000
**16**	POS	271.1660	3.28	Unknown	Unidentified (3)	n/a		Level 4	2.39	-2.39	0.000
**17**	POS	279.2327	14.00	[M+H]+	Fatty acid (1) (Pinolenic Acid / Linolenic acid)	C01595	Linoleic acid metabolism (map00591)	Level 3	2.56	-2.56	0.001
**18**	POS	281.2483	13.18	[M+H]+	Fatty acid (2) (Conjugated Linoleic Acid / Linoelaidic Acid / Linoleic acid)	n/a	Fatty acids and conjugates metabolism	Level 3	-0.36	0.36	0.012
**19**	POS	283.2639	14.41	[M+H]+	Fatty acid (4) Oleic Acid, Elaidic Acid, Vaccenic acid (RT14.41)	C00712	Fatty acid biosynthesis (map00061)	Level 3	2.93	-2.93	0.001
**20**	POS	283.2641	14.16	[M+H]+	Fatty acid (5) Oleic Acid, Elaidic Acid, Vaccenic acid (RT14.16)	C00712	Fatty acid biosynthesis (map00061)	Level 3	-1.02	1.02	0.007
**21**	POS	293.2120	12.10	[M+H]+	Oxylipin (1) (9-OxoOTrE)	n/a	PUFA derived oxylipins	Level 3	-0.82	0.82	0.016
**22**	POS	294.1546	0.89	[M+H]+	N-(1-Deoxy-1-fructosyl)isoleucine/ N-(1-Deoxy-1-fructosyl)leucine	n/a		Level 2	-0.86	0.86	0.000
**23**	POS	295.0968	6.13	[M+H]+	Isoflavone	n/a		Level 3	0.02	-0.02	0.005
**24**	POS	297.2430	10.40	[M+H]+	Oxylipin (3) (9-HODE, 13-HODE)	n/a	PUFA derived oxylipins	Level 3	-0.15	0.15	0.000
**25**	POS	297.2431	11.27	[M+H]+	Oxylipin (4) (9-HODE, 13-HODE)	n/a	PUFA derived oxylipins	Level 3	-0.02	0.02	0.062

**26**	POS	298.0974	2.90	[M+H]+	5′-Deoxy-5′-(methylthio)adenosine	n/a		Level 2	-0.04	0.04	0.012
**27**	POS	299.2589	13.41	[M+H]+	Oxylipin (5) (HOME, 13-oxo-octadecanoic acid, 12-oxo-octadecanoic acid)	n/a	PUFA derived oxylipins	Level 3	-0.28	0.28	0.000
**28**	POS	301.2744	14.41	[M+NH4]+	Fatty acid (6) Oleic Acid, Elaidic Acid, Vaccenic acid (RT14.41)	C00712	Fatty acid biosynthesis (map00061)	Level 3	0.58	-0.58	0.001
**29**	POS	311.2222	10.20	[M+H]+	Oxylipin (8) (9(S)-HpOTrE)	n/a	PUFA derived oxylipins	Level 3	-0.46	0.46	0.000
**30**	POS	313.2747	13.79	[M+H]+	Ricinoleic Acid methyl ester	n/a		Level 2	-0.39	0.39	0.000
**31**	POS	315.1963	12.44	[M+H]+	Lys Pro Ala	n/a		Level 2	0.02	-0.02	0.000
**32**	POS	315.2536	10.47	[M+H]+	Oxylipin (9) ((±)12,13-DiHOME, 9,10-DiHOME)	C14829	Linoleic acid metabolism (map00591)	Level 3	-0.08	0.08	0.000
**33**	POS	319.2253	13.97	[M+H]+	Oxylipin (10) (HEPE, Oxo-ETE, EpETE)	n/a	PUFA derived oxylipins	Level 3	2.81	-2.81	0.000
**34**	POS	324.1448	2.69	[M+H]+	Ferulic acid + L-Pipecolic acid	C01494	Phenylpropanoid biosynthesis (map00940)	Level 3	-0.06	0.06	0.000
**35**	POS	335.2202	13.12	[M+H]+	Oxylipin (14) (HpEPE)	n/a	PUFA derived oxylipins	Level 3	0.18	-0.18	0.000
**36**	POS	335.2203	13.38	[M+H]+	Oxylipin (15) (HpEPE)	n/a	PUFA derived oxylipins	Level 3	0.04	-0.04	0.002
**37**	POS	337.2361	12.01	[M+H]+	Oxylipin (16) (DiHETE, HpETE)	n/a	PUFA derived oxylipins	Level 3	0.49	-0.49	0.000
**38**	POS	347.2432	7.88	[M+H]+	9,10-dihydroxy-Octadecanedioic acid	n/a	Fatty acids and conjugates metabolism	Level 2	-0.19	0.19	0.000
**39**	POS	351.2151	10.22	[M+Na]+	Oxylipin (17) (9,12,13,TriHODE; m/z 329.2336)	n/a	PUFA derived oxylipins	Level 3	-0.01	0.01	0.001
**40**	POS	365.3061	14.79	[M+H]+	2-Arachidonyl Glycerol ether	n/a	Fatty acids and conjugates metabolism	Level 2	0.20	-0.20	0.000
**41**	POS	379.2853	14.19	[M+H]+	Norchenodeoxycholic acid	n/a	Bile acid metabolism	Level 2	0.05	-0.05	0.000
**42**	POS	379.2962	8.38	Unknown	Unidentified (5)	n/a		Level 4	0.79	-0.79	0.000
**43**	POS	393.3374	16.23	[M+NH4]+	Docosatetraenoyl Ethanolamide (DEA) (m/z 375.3264)	n/a	Fatty acids and conjugates metabolism	Level 2	1.64	-1.64	0.000
**44**	POS	407.3167	15.58	[M+H]+	Fatty acid (9) (MG(22:4/0:0/0:0))	n/a	Glycerophospholipid metabolism	Level 3	0.05	-0.05	0.030
**45**	POS	411.3269	17.92	[M+H]+	γ-Tocotrienol	C14155	Ubiquinone and other terpenoid-quinone biosynthesis (map00130)	Level 2	0.77	-0.77	0.000
**46**	POS	454.2939	12.39	[M+H]+	PE(16:0/0:0)	n/a	Glycerophospholipid metabolism	Level 2	-0.20	0.20	0.002
**47**	POS	500.3378	10.70	[M+NH4]+	PC(15:0/0:0) (m/z 482.3287)	C04230	Glycerophospholipid metabolism (map00564)	Level 2	0.37	-0.37	0.000
**48**	POS	595.3495	6.75	[M+H]+	Urobilin	C05793	Porphyrin and chlorophyll metabolism (map00860)	Level 2	1.41	-1.41	0.004
**49**	POS	601.5415	14.41	[2M+H]+	Fatty acid (10) Oleic Acid, Elaidic Acid, Vaccenic acid (RT14.41)	C00712	Fatty acids and conjugates metabolism	Level 3	1.22	-1.22	0.001
**50**	POS	100.0759	2.07	[M+H]+	δ-Valerolactam			Level 1	0.04	-0.04	0.661
**51**	POS	116.0707	0.74	[M+H]+	Proline	C00148	map00330 Arginine and proline metabolism	Level 1	0.11	-0.11	0.405
**52**	POS	282.2801	16.28	[M+H]+	Fatty acid (3) Oleamide / Elaidamide	C19670	Fatty acids and conjugates metabolism	Level 3	0.08	-0.08	0.631

**53**	POS	295.2277	13.42	[M+H]+	Oxylipin (2) (13-OxoODE, 9-OxoODE, 9(S)-HOTrE)	n/a	PUFA derived oxylipins	Level 3	0.09	-0.09	0.103
**54**	POS	299.2589	14.88	[M+H]+	Oxylipin (6) (HOME, 13-oxo-octadecanoic acid, 12-oxo-octadecanoic acid)	n/a	PUFA derived oxylipins	Level 3	-1.96	1.96	0.120
**55**	POS	299.2589	14.64	[M+H]+	Oxylipin (7) (HOME, 13-oxo-octadecanoic acid, 12-oxo-octadecanoic acid)	n/a	PUFA derived oxylipins	Level 3	-0.14	0.14	0.151
**56**	POS	319.2253	14.21	[M+H]+	Oxylipin (11) (HEPE, Oxo-ETE, EpETE)	n/a	PUFA derived oxylipins	Level 3	-0.55	0.55	0.656
**57**	POS	321.2409	14.64	[M+Na]+	Oxylipin (12) (HOME, 13-oxo-octadecanoic acid, 12-oxo-octadecanoic acid)	n/a	PUFA derived oxylipins	Level 3	-0.04	0.04	0.104
**58**	POS	321.2409	14.88	[M+Na]+	Oxylipin (13) (HOME, 13-oxo-octadecanoic acid, 12-oxo-octadecanoic acid)	n/a	PUFA derived oxylipins	Level 3	-0.17	0.17	0.334
**59**	POS	324.2906	14.36	[M+H]+	Linoleoyl Ethanolamide	n/a	Fatty acids and conjugates metabolism	Level 2	0.08	-0.08	0.200
**60**	POS	350.3427	17.80	Unknown	Unidentified (4)	n/a		Level 4	0.03	-0.03	0.218
**61**	POS	357.2793	9.97	[M+H]+	Fatty acid (7) (Docosahexaenoic Acid ethyl ester / Tetracosahexaenoic acid)	n/a	Fatty acids and conjugates metabolism	Level 3	0.00	0.00	0.661
**62**	POS	357.2796	11.54	[M+H]+	Fatty acid (8) (Docosahexaenoic Acid ethyl ester / Tetracosahexaenoic acid)	n/a	Fatty acids and conjugates metabolism	Level 3	-0.06	0.06	0.755
**63**	POS	373.2747	13.21	[M+H]+	3-OXO-CHOL-11-ENIC ACID	n/a	Bile acid metabolism	Level 2	0.15	-0.15	0.132
**64**	POS	381.3117	7.39	[M+NH4]+	Oxylipin (18) (15(S)-HETE Ethanolamide)	n/a	PUFA derived oxylipins	Level 3	-0.02	0.02	0.362
**65**	POS	391.2851	10.36	[M+H]+	Nutriacholic acid	n/a	Bile acid metabolism	Level 2	-0.30	0.30	0.366
**66**	POS	407.2798	8.53	[M+H]+	Bile acid (1) (3-Oxocholic acid / 7-ketodeoxycholic acid)	n/a	Bile acid metabolism	Level 3	-0.03	0.03	0.284
**67**	POS	505.3389	4.65	Unknown	Unidentified (6)	n/a		Level 4	-0.10	0.10	0.520
**68**	POS	561.4891	13.67	Unknown	Unidentified (7)	n/a		Level 4	-0.04	0.04	0.904
**69**	NEG	89.0244	0.89	[M-H]-	Lactic acid	C00256	Pyruvate metabolism (map00620)	Level 2	-0.71	0.71	0.000
**70**	NEG	117.0194	1.20	[M-H]-	Succinic acid	C00042	Citrate cycle (TCA cycle) (map00020)	Level 1	-2.15	2.15	0.000
**71**	NEG	117.0559	2.82	[M-H]-	2-Hydroxy-3-methylbutyric acid (2-hydroxyisovaleric acid)	n/a		Level 1	0.50	-0.50	0.000
**72**	NEG	131.0352	2.34	[M-H]-	Methylsuccinic acid	n/a		Level 2	-0.12	0.12	0.000
**73**	NEG	131.0716	4.29	[M-H]-	2-Hydroxyisocaproic acid	n/a		Level 1	0.36	-0.36	0.000
**74**	NEG	147.0300	0.89	[M-H]-	2-Hydroxy-glutarate	C03196	Lysine degradation (map00310)	Level 1	-1.09	1.09	0.000
**75**	NEG	151.0262	0.89	[M-H]-	Xanthine	C00385	Purine metabolism (map00230)	Level 1	0.41	-0.41	0.003
**76**	NEG	159.0666	3.86	[M-H]-	Pimelic acid	C02656	Biotin metabolism (map00780)	Level 1	0.51	-0.51	0.000
**77**	NEG	159.0666	4.63	[M-H]-	3,3-Dimethylglutaric acid / 3-Methyladipic acid			Level 2	-6.89	6.89	0.000

**78**	NEG	165.0560	5.03	[M-H]-	Phenyllactic acid	n/a		Level 1	-1.63	1.63	0.000
**79**	NEG	173.0822	5.96	[M-H]-	2,4-Dimethyladipic acid / Ethyladipic acid	n/a		Level 2	-0.43	0.43	0.000
**80**	NEG	173.0822	4.92	[M-H]-	Suberic acid	C08278	Fatty acids and conjugates metabolism	Level 2	0.01	-0.01	0.029
**81**	NEG	181.0510	3.13	[M-H]-	Hydroxyphenyllactic acid	C03672		Level 1	0.05	-0.05	0.000
**82**	NEG	181.0511	3.82	[M-H]-	3,4-Dihydroxyhydrocinnamic acid	C10447	Tyrosine metabolism (map00350)	Level 1	0.85	-0.85	0.048
**83**	NEG	188.0566	0.89	[M-H]-	N-Acetyl-L-glutamic acid / Glutarylglycine	C00624	Arginine biosynthesis (map00220)	Level 2	-0.03	0.03	0.055
**84**	NEG	188.0566	1.08	[M-H]-	N-Acetyl-L-glutamic acid / Glutarylglycine	C00624	Arginine biosynthesis (map00220)	Level 2	-0.22	0.22	0.000
**85**	NEG	201.1135	6.86	[M-H]-	Sebacic acid	C08277	Fatty acids and conjugates metabolism	Level 1	0.35	-0.35	0.004
**86**	NEG	203.0564	2.75	[M-H]-	L-Ascorbic acid ethyl ester	n/a		Level 2	-0.45	0.45	0.000
**87**	NEG	215.1292	7.76	[M-H]-	Undecanedioic acid	n/a		Level 2	1.23	-1.23	0.000
**88**	NEG	218.1037	2.32	[M-H]-	Pantothenic Acid	C00864	Pantothenate and CoA biosynthesis (map00770)	Level 1	-1.62	1.62	0.000
**89**	NEG	241.1085	4.85	Unknown	Unidentified (2)	n/a		Level 4	0.93	-0.93	0.000
**90**	NEG	243.1240	6.44	[M-H]-	Fatty acid (3) (4-Oxododecanedioic acid)	n/a	Fatty acids and conjugates metabolism	Level 3	-0.33	0.33	0.000
**91**	NEG	243.1241	5.92	[M-H]-	Fatty acid (2) (4-Oxododecanedioic acid)	n/a	Fatty acids and conjugates metabolism	Level 3	-0.20	0.20	0.000
**92**	NEG	289.1660	5.60	[M-H]-	2,2-bis(2-hydroxyethyl)sebacic acid	n/a	Fatty acids and conjugates metabolism	Level 2	0.52	-0.52	0.001
**93**	NEG	293.2125	13.30	[M-H]-	Oxylipin (1) (9-OxoODE)	n/a	PUFA derived oxylipins	Level 3	0.72	-0.72	0.006
**94**	NEG	295.2283	12.80	[M-H]-	Oxylipin (2) (13-HODE)	n/a	PUFA derived oxylipins	Level 3	2.23	-2.23	0.002
**95**	NEG	297.1135	8.14	[M-H]-	(±)-Enterolactone	n/a		Level 1	5.31	-5.31	0.000
**96**	NEG	297.2438	14.80	[M-H]-	Fatty acid (4) (12-oxo-octadecanoic acid / 6R,7S-Epoxy-octadecanoate)	n/a	Fatty acids and conjugates metabolism	Level 3	-2.76	2.76	0.097
**97**	NEG	299.1867	8.74	[M-H]-	13,14-dihydro-15-keto-tetranor PGF1β	n/a		Level 2	-0.46	0.46	0.076
**98**	NEG	299.2595	14.30	[M-H]-	(R)-10-hydroxystearic acid	n/a	Fatty acids and conjugates metabolism	Level 2	0.56	-0.56	0.001
**99**	NEG	307.1919	11.70	[M-H]-	Corchorifatty acid A	n/a	Fatty acids and conjugates metabolism	Level 2	3.48	-3.48	0.000
**100**	NEG	311.2230	12.60	[M-H]-	Oxylipin (3) ((±)13-HpODE / (±)9-HpODE)	n/a	PUFA derived oxylipins	Level 3	-2.20	2.20	0.000
**101**	NEG	311.2231	11.40	[M-H]-	Oxylipin (4) (9-HpODE / 13-HpODE)	n/a	PUFA derived oxylipins	Level 3	0.35	-0.35	0.051
**102**	NEG	315.1242	5.78	[M-H]-	Verimol B	n/a		Level 2	0.33	-0.33	0.000
**103**	NEG	315.2538	9.97	[M-H]-	Fatty acid (5) (9,10-Dihydroxystearic acid)	n/a	Fatty acids and conjugates metabolism	Level 2	-0.41	0.41	0.000
**104**	NEG	315.2541	10.50	[M-H]-	Fatty acid (6) (12,13-dihydroxy stearic acid)	n/a	Fatty acids and conjugates metabolism	Level 3	-0.31	0.31	0.033
**105**	NEG	315.2544	11.60	[M-H]-	Fatty acid (7) (11,12-dihydroxy stearic acid)	n/a	Fatty acids and conjugates metabolism	Level 3	0.54	-0.54	0.000

**106**	NEG	319.1666	6.54	Unknown	Unidentified (3)	n/a		Level 4	-0.25	0.25	0.023
**107**	NEG	327.0879	5.66	[M-H]-	Isoflavone (1)	n/a	Fatty acids and conjugates metabolism	Level 3	1.70	-1.70	0.000
**108**	NEG	327.2179	10.10	[M-H]-	Corchorifatty acid F	n/a	Fatty acids and conjugates metabolism	Level 2	-1.91	1.91	0.000
**109**	NEG	327.2180	8.96	[M-H]-	Prostaglandin (2,3-Dinor-8-iso prostaglandin F1alpha)	n/a		Level 3	-1.35	1.35	0.000
**110**	NEG	329.1034	6.06	[M-H]-	Isoflavone (2) (Samaderin A)	n/a		Level 3	5.11	-5.11	0.000
**111**	NEG	329.2336	9.71	[M-H]-	Oxylipin (6) (9,10,13-TriHOME/11,12,13-TriHOME/5,8,12-TriHOME)	n/a	PUFA derived oxylipins	Level 3	-1.55	1.55	0.000
**112**	NEG	329.2336	8.79	[M-H]-	Oxylipin (8) (9,10,13-TriHOME/11,12,13-TriHOME/5,8,12-TriHOME)	n/a	PUFA derived oxylipins	Level 3	0.69	-0.69	0.007
**113**	NEG	329.2337	8.14	[M-H]-	Oxylipin (7) (9,10,13-TriHOME/11,12,13-TriHOME/5,8,12-TriHOME)	n/a	PUFA derived oxylipins	Level 3	-3.07	3.07	0.000
**114**	NEG	333.0902	8.14	[M+Cl]-	Enterolactone	n/a		Level 1	0.04	-0.04	0.000
**115**	NEG	345.2285	7.85	[M-H]-	9,10-Dihydroxyoctadecanedioic acid	n/a	Fatty acids and conjugates metabolism	Level 2	-4.63	4.63	0.000
**116**	NEG	349.2388	12.60	[M-H]-	Tetrahydrocorticosterone	n/a		Level 2	0.12	-0.12	0.000
**117**	NEG	377.2000	8.68	Unknown	Unidentified (5)	n/a		Level 4	0.07	-0.07	0.000
**118**	NEG	377.2006	8.90	Unknown	Unidentified (6)	n/a		Level 4	1.40	-1.40	0.000
**119**	NEG	387.2544	11.10	[M-H]-	Bile acid (2) (7alpha-hydroxy-3-oxochol-4-en-24-oic Acid)	n/a	Bile acid metabolism	Level 2	0.62	-0.62	0.045
**120**	NEG	391.2857	11.70	[M-H]-	Deoxycholic acid	n/a	Bile acid metabolism	Level 1	-0.14	0.14	0.090
**121**	NEG	397.1691	14.80	Unknown	Unidentified (7)	n/a		Level 4	-0.31	0.31	0.087
**122**	NEG	409.1904	7.59	Unknown	Unidentified (8)	n/a		Level 4	0.02	-0.02	0.000
**123**	NEG	409.1905	7.19	Unknown	Unidentified (9)	n/a		Level 4	0.35	-0.35	0.000
**124**	NEG	433.2598	11.10	[M+FA]-	Bile acid (8) (7alpha-hydroxy-3-oxochol-4-en-24-oic Acid)	n/a	Bile acid metabolism	Level 3	0.08	-0.08	0.049
**125**	NEG	453.2860	9.21	[M+FA]-	Bile acid (13) (Allocholic acid/Hyocholic acid/Muricholic acid/Ursocholic acid)	n/a	Bile acid metabolism	Level 3	-2.61	2.61	0.020
**126**	NEG	479.3016	11.70	[M-H]-	LysoPE(0:0/18:2(9Z,12Z))	n/a	Fatty acids and conjugates metabolism	Level 2	-0.48	0.48	0.000
**127**	NEG	523.3128	12.60	Unknown	Unidentified (10)	n/a		Level 4	-0.02	0.02	0.000
**128**	NEG	537.3285	13.70	[M-H]-	Fatty acid (9) (PG(20:1(11Z)/0:0))	n/a	Fatty acids and conjugates metabolism	Level 2	-0.51	0.51	0.000
**129**	NEG	659.2134	6.09	[2M+H]-	Isoflavone (3) (Samaderin A)	n/a		Level 3	0.03	-0.03	0.000
**130**	NEG	129.0560	3.42	[M-H]-	Fatty acid (1) (3-Methyl-2-oxovaleric acid / 2-keto-n-caproic acid)	n/a	Fatty acids and conjugates metabolism	Level 3	0.29	-0.29	0.280
**131**	NEG	129.0560	3.84	[M-H]-	Ketoleucine	C00233	Valine, leucine and isoleucine degradation (map00280)	Level 1	0.53	-0.53	0.373
**132**	NEG	146.0459	0.70	[M-H]-	Glutamate	C00025	Alanine, aspartate and glutamate metabolism (map00250)	Level 1	-0.82	0.82	0.724
**133**	NEG	235.0827	2.30	[M-H]-	Purine deoxyribonucleoside	n/a		Level 2	0.19	-0.19	0.363

**134**	NEG	241.1084	6.87	Unknown	Unidentified (1)	n/a		Level 4	0.03	-0.03	0.152
**135**	NEG	297.2439	13.30	[M-H]-	Ricinoleic acid	C08365	Fatty acids and conjugates metabolism	Level 2	-0.37	0.37	0.224
**136**	NEG	301.1662	5.18	[M-H]-	(2S,3S)-3-hydroxy-2-(9-ketodecyl)glutaric acid	n/a		Level 2	0.10	-0.10	0.242
**137**	NEG	313.2387	11.00	[M-H]-	Oxylipin (5) (12,13-DiHOME)	n/a	PUFA derived oxylipins	Level 3	0.35	-0.35	0.565
**138**	NEG	315.2543	11.90	[M-H]-	Fatty acid (8) (11,12-dihydroxy stearic acid)	n/a	Fatty acids and conjugates metabolism	Level 3	0.26	-0.26	0.128
**139**	NEG	375.1850	8.51	Unknown	Unidentified (4)	n/a		Level 4	0.69	-0.69	0.801
**140**	NEG	375.2909	14.00	[M-H]-	Bile acid (1) (Allolithocholic acid / Lithocholic acid)	n/a	Bile acid metabolism	Level 3	0.00	0.00	0.865
**141**	NEG	389.2699	10.30	[M-H]-	Bile acid (3) (Nutriacholic acid / 12-Ketodeoxycholic acid)	n/a	Bile acid metabolism	Level 3	-1.08	1.08	0.304
**142**	NEG	391.2856	9.92	[M-H]-	Ursodeoxycholic acid	n/a	Bile acid metabolism	Level 1	1.99	-1.99	0.683
**143**	NEG	407.2804	9.23	[M-H]-	Bile acid (4) (Allocholic acid/Hyocholic acid/Muricholic acid/Ursocholic acid)	n/a	Bile acid metabolism	Level 3	-0.56	0.56	0.445
**144**	NEG	407.2805	8.09	[M-H]-	Bile acid (5) (Allocholic acid/Hyocholic acid/Muricholic acid/Ursocholic acid)	n/a	Bile acid metabolism	Level 3	0.08	-0.08	0.724
**145**	NEG	407.2805	8.35	[M-H]-	Bile acid (6) (Allocholic acid/Hyocholic acid/Muricholic acid/Ursocholic acid)	n/a	Bile acid metabolism	Level 3	0.36	-0.36	0.836
**146**	NEG	421.2963	14.00	[M+FA]-	Bile acid (7) (Allolithocholic acid / Lithocholic acid)	n/a	Bile acid metabolism	Level 3	0.20	-0.20	0.859
**147**	NEG	435.2753	10.30	[M+FA]-	Bile acid (9) (Nutriacholic acid / 12-Ketodeoxycholic acid)	n/a	Bile acid metabolism	Level 3	-1.04	1.04	0.317
**148**	NEG	437.2906	10.70	[M+FA]-	Bile acid (10) (Murocholic acid, m/z 391.2855)	n/a	Bile acid metabolism	Level 3	0.75	-0.75	0.797
**149**	NEG	437.2910	11.50	[M+FA]-	Chenodeoxycholic acid	n/a	Bile acid metabolism	Level 1	-1.07	1.07	0.841
**150**	NEG	437.2910	9.92	[M+FA]-	Ursodeoxycholic acid	n/a	Bile acid metabolism	Level 1	3.01	-3.01	0.888
**151**	NEG	437.2924	12.60	[M+FA]-	Bile acid (11)	n/a	Bile acid metabolism	Level 3	-0.23	0.23	0.664
**152**	NEG	453.2860	8.40	[M+FA]-	Bile acid (12) (Allocholic acid/Hyocholic acid/Muricholic acid/Ursocholic acid)	n/a	Bile acid metabolism	Level 3	0.25	-0.25	0.420

1 Ionization mode

2 MS M/Z, mass spectrometry mass-to-charge ratio

3 RT, retention time

4Abbreviations: FA, formic acid; OxoOTre, oxo-octadecatrienoic acid; HODE, hydroxyoctadecadienoic acid; HOME, Hydroxy-octadecenoic acid; HpHOTrE, hydroperoxy-octadecatrienoic acid; DiHOME, dihydroxy-octadecenoic acid; HEPE, hydroxy-eicosapentaenoic acid; HpEPE, hydroperoxy-eicosapentaenoic acid; DiHETE, dihydroxy-eicosatetraenoic acid; HpETE, hydroperoxy-eicosatetraenoic acid; TriHODE, trihydroxy-octadecenoic acid; MG, monoacylglyceride; PE, glycerophosphatidylethanolamine; PC, phosphocholine; OxoODE, oxo-octadecadienoic acid; HETE, hydroxy-eicosatetraenoic acid; HpODE, Hydroperoxy-octadecadienoic acid; TriHOME, trihydroxy-octadecenoic acid; LysoPE, Lysophosphatidylethanolamine; PG, phosphoglycerol; LysoPC, lysophosphatidylcholine.

5 KEGG, Kyoto Encyclopedia of Genes and Genomes; n/a, not available

6 RC HR/LR, regression coefficient for the HR/LR group from the MBPLSR models

Table S4. sMBPLSR metabolites discriminating between different collection time points analyzed in plasma, urine, and feces over a five-month dietary intervention period

LIST NR	IM[1]	MS M/Z[2]	RT[3]	ION	METABOLITE[4]	KEGG[5]	PATHWAY	ID LEVEL	RC[6] WEEK 4	RC[6] WEEK 12	RC[6] WEEK 20	P-VAL W4/W12	P-VAL W12/W20	P-VAL W4/W20
							**PLASMA**							
**1**	POS	100.0760	2.33	[M+H]+	δ-Valerolactam	n/a		Level 1	-0.34	-1.30	1.65	0.631	0.071	0.383
**2**	POS	102.0916	1.13	[M+H]+	Betaine aldehyde	C00576	Glycine, serine and threonine metabolism (map00260)	Level 2	0.01	-0.02	0.01	0.968	0.936	0.824
**3**	POS	104.0709	0.80	[M+H]+	γ-aminobutyric acid	C00334	Alanine, aspartate and glutamate metabolism (map00250)	Level 2	-0.32	-0.06	0.38	0.997	0.031	0.024
**4**	POS	104.1072	0.76	[M+H]+	Choline	C00114	Glycine, serine and threonine metabolism (map00260)	Level 1	0.32	0.06	-0.38	0.103	0.073	0.000
**5**	POS	118.0865	0.80	[M+H]+	Betaine	C00719	Glycine, serine and threonine metabolism (map00260)	Level 1	1.53	0.79	-2.32	0.124	0.017	0.000
**6**	POS	130.0502	0.78	[M+H]+	Pyroglutamic acid	C02237	D-Glutamine and D-glutamate metabolism (map00471)	Level 1	-0.07	0.13	-0.06	0.140	0.987	0.184
**7**	POS	132.0770	0.82	[M+H]+	Creatine	C00300	Glycine, serine and threonine metabolism (map00260)	Level 1	0.23	0.04	-0.27	0.584	0.045	0.003
**8**	POS	132.1023	1.44	[M+H]+	Leucine	C00123	Valine, leucine and isoleucine degradation (map00280)	Level 1	-1.91	-0.47	2.38	0.077	0.001	0.000
**9**	POS	137.0460	1.10	[M+H]+	Hypoxanthine	C00262	Purine metabolism (map00230)	Level 1	0.11	-0.10	-0.01	0.251	0.638	0.035
**10**	POS	148.0972	1.13	[M+H]+	4-Hydroxyisoleucine	n/a		Level 1	0.13	-0.25	0.11	0.939	0.840	0.635
**11**	POS	150.0587	1.09	[M+H]+	Methionine	C00073	Cysteine and methionine metabolism (map00270)	Level 1	-0.03	0.05	-0.02	0.607	0.969	0.752
**12**	POS	159.0657	5.22	Unknown	Unidentified (1)	n/a		Level 4	0.11	-0.20	0.09	0.000	0.000	0.478
**13**	POS	160.0762	2.10	[M+H]+	Indoleacetaldehyde	C00637	Tryptophan metabolism (map00380)	Level 2	0.01	-0.02	0.01	0.994	0.903	0.853
**14**	POS	166.0868	2.35	[M+H]+	Phenylalanine	C00079	Phenylalanine metabolism (map00360)	Level 1	0.16	-0.59	0.42	0.649	0.070	0.365
**15**	POS	180.0659	3.79	[M+H]+	Hippuric acid	C01586	Phenylalanine metabolism (map00360)	Level 1	0.02	0.00	-0.03	0.019	0.411	0.000
**16**	POS	205.0978	3.08	[M+H]+	Tryptophan	C00078	Tryptophan metabolism (map00380)	Level 1	-0.79	-0.15	0.94	0.323	0.058	0.001
**17**	POS	209.1908	8.71	[M+H]+	n,n-Tetradecadienal	n/a	Fatty acids and conjugates metabolism	Level 2	0.26	-0.48	0.22	0.000	0.000	0.620
**18**	POS	217.1077	4.84	[M+H]+	γ-Glutamyl-γ-aminobutyraldehyde	C15700	Arginine and proline metabolism (map00330)	Level 2	-2.14	1.56	0.57	0.000	0.404	0.000
**19**	POS	239.0896	4.84	[M+Na]+	γ-Glutamyl-γ-aminobutyraldehyde	C15700	Arginine and proline metabolism (map00330)	Level 2	-1.98	1.30	0.68	0.000	0.224	0.000
**20**	POS	249.1835	8.72	Unknown	Unidentified (2)			Level 3	0.07	-0.13	0.06	0.000	0.004	0.224
**21**	POS	255.0635	4.84	[M+K]+	γ-Glutamyl-γ-aminobutyraldehyde	C15700	Arginine and proline metabolism (map00330)	Level 2	-0.28	0.15	0.12	0.000	0.094	0.000

**22**	POS	269.0886	1.53	[M+H]+	Inosine	C00294	Purine metabolism (map00230)	Level 1	0.05	-0.09	0.04	0.351	0.901	0.163
**23**	POS	294.1555	1.35	[M+H]+	N-(1-Deoxy-1-fructosyl)isoleucine / N-(1-Deoxy-1-fructosyl)leucine	n/a		Level 2	-0.08	-0.01	0.09	0.492	0.028	0.001
**24**	POS	305.1418	4.14	Unknown	Unidentified (3)	n/a		Level 4	-0.15	0.27	-0.13	0.012	0.086	0.704
**25**	POS	357.2800	7.96	[M+H]+	Fatty acid (1) (Tetracosahexaenoic acid / Docosahexaenoic Acid ethyl ester)	n/a	Fatty acids and conjugates metabolism	Level 2	0.09	0.02	-0.10	0.985	0.841	0.743
**26**	POS	376.2611	8.62	[M+H-H2O]+	Sphingosine-1-phosphate	n/a	Fatty acids and conjugates metabolism	Level 3	-0.05	0.09	-0.04	0.109	0.070	0.977
**27**	POS	391.2855	8.29	[M+H]+	Bile acid (Nutriacholic acid / 12-Ketodeoxycholic acid)	n/a	Bile acid metabolism	Level 2	0.08	0.02	-0.10	0.455	0.996	0.401
**28**	POS	455.1899	4.84	[2M+Na]	γ-Glutamyl-γ-aminobutyraldehyde	C15700	Arginine and proline metabolism (map00330)	Level 2	-0.55	0.30	0.24	0.000	0.304	0.000
**29**	POS	479.1584	4.30	[M+H]+	Glucuronated Cmpd (1) (4'-O-methyl-(-)-epicatechin-5-O-beta-glucuronide)	n/a		Level 2	-0.02	0.00	0.03	0.257	0.014	0.000
**30**	NEG	103.0401	1.79	[M-H]-	2-Hydroxybutyrate	C05984	Propanoate metabolism (map00640)	Level 1	-0.23	-0.03	0.26	0.308	0.880	0.129
**31**	NEG	115.0402	2.29	[M-H]-	α-ketoisovaleric acid	C00141	Valine, leucine and isoleucine degradation (map00280)	Level 1	-0.23	-0.03	0.26	0.470	0.182	0.011
**32**	NEG	116.0717	0.96	[M-H]-	Valine	C00183	Valine, leucine and isoleucine degradation (map00280)	Level 1	-0.12	-0.02	0.14	0.037	0.000	0.000
**33**	NEG	117.0558	2.99	[M-H]-	a-Hydroxyisovaleric acid	n/a		Level 1	-0.73	-0.10	0.82	0.002	0.104	0.000
**34**	NEG	128.0354	1.20	[M-H]-	Pyroglutamic acid	C02237	D-Glutamine and D-glutamate metabolism (map00471)	Level 1	-0.95	-0.13	1.07	0.000	0.027	0.000
**35**	NEG	129.0559	3.56	[M-H]-	Ketoisoleucine (3-Methyl-2-oxovaleric acid)	C00671	Valine, leucine and isoleucine degradation (map00280)	Level 1	-1.00	-0.13	1.13	0.177	0.663	0.025
**36**	NEG	129.0559	3.91	[M-H]-	Ketoleucine	C00233	Valine, leucine and isoleucine degradation (map00280)	Level 1	-2.35	-0.31	2.66	0.365	0.072	0.002
**37**	NEG	130.0875	1.46	[M-H]-	Leucine	C00123	Valine, leucine and isoleucine degradation (map00280)	Level 1	-1.17	-0.16	1.32	0.061	0.000	0.000
**38**	NEG	131.0715	4.08	[M-H]-	2-Hydroxyisocaproic acid	n/a		Level 1	-0.32	-0.04	0.36	0.002	0.004	0.000
**39**	NEG	133.0143	1.03	[M-H]-	Malic acid	C00149	Citrate cycle (TCA cycle) (map00020)	Level 1	0.12	0.02	-0.13	0.037	0.000	0.000
**40**	NEG	145.0144	1.08	[M-H]-	Ketoglutaric acid (oxoglutaric acid)	C00026	D-Glutamine and D-glutamate metabolism (map00471)	Level 1	0.85	0.11	-0.96	0.194	0.000	0.000
**41**	NEG	145.0619	0.80	[M-H]-	Glutamine	C00064	D-Glutamine and D-glutamate metabolism (map00471)	Level 2	-0.12	-0.02	0.13	0.033	0.009	0.000
**42**	NEG	146.0459	0.82	[M-H]-	Glutamate	C00025	D-Glutamine and D-glutamate metabolism (map00471)	Level 1	-0.06	-0.01	0.07	0.067	0.362	0.001
**43**	NEG	160.0615	0.84	[M-H]-	2-Aminoadipic acid	C00956	Lysine degradation (map00310)	Level 1	-0.03	0.00	0.03	0.680	0.177	0.027
**44**	NEG	164.0719	2.34	[M-H]-	Phenylalanine	C00079	Phenylalanine metabolism (map00360)	Level 1	-0.01	0.00	0.02	1.000	0.043	0.041
**45**	NEG	178.0512	3.80	[M-H]-	Hippuric acid	C01586	Phenylalanine metabolism (map00360)	Level 1	1.18	0.16	-1.34	0.312	0.191	0.005
**46**	NEG	187.0074	4.47	[M-H]-	p-Cresol sulfate	n/a		Level 1	-0.15	-0.02	0.17	0.019	0.507	0.001

**47**	NEG	199.0978	5.49	[M-H]-	cis-4-Decenedioic acid	n/a		Level 2	0.09	0.01	-0.11	0.559	0.005	0.000
**48**	NEG	203.0828	3.10	[M-H]-	Tryptophan	C00078	Tryptophan metabolism (map00380)	Level 1	-2.04	-0.27	2.32	0.028	0.069	0.000
**49**	NEG	212.0025	3.89	[M-H]-	Indoxyl sulfate	n/a		Level 1	-0.66	-0.09	0.75	0.495	0.053	0.002
**50**	NEG	215.0328	0.81	[M+Cl]-	Fructose	C02336	Amino sugar and nucleotide sugar metabolism (map00520)	Level 1	-0.98	-0.13	1.11	0.118	0.925	0.051
**51**	NEG	230.9970	4.41	[M-H]-	Vanillin 4-sulfate / p-Hydroxyphenylacetic acid sulphate / 2,4-Dihydroxyacetophenone 5-sulfate	C00755	Phenylalanine metabolism (map00360) or C00755	Level 2	-1.13	-0.15	1.28	0.335	0.071	0.001
**52**	NEG	245.0491	5.51	[M+SO3H]-	4-Butoxyphenol or 4-n-Butylresorcinol (m/z 165.0929)	n/a		Level 2	-0.26	-0.03	0.29	0.610	1.000	0.615
**53**	NEG	249.0229	6.26	[M-H]-	4,4'-Sulfonyldiphenol	n/a		Level 2	-4.27	-0.57	4.84	0.000	0.560	0.000
**54**	NEG	255.9599	1.44	Unknown	Unidentified (1)	n/a		Level 4	-0.56	-0.07	0.63	0.001	0.000	0.000
**55**	NEG	257.9570	1.44	Unknown	Unidentified (2)	n/a		Level 4	-0.33	-0.04	0.37	0.000	0.000	0.000
**56**	NEG	276.0171	1.46	[M+SO3H]-	L-Tyrosine / N-Hydroxy-L-phenylalanine (m/z 180.0669)	C00082	Tyrosine metabolism (map00350)	Level 2	-0.15	-0.02	0.17	0.000	0.000	0.000
**57**	NEG	351.0778	1.47	Unknown	Unidentified (3)	n/a		Level 4	-0.14	-0.02	0.16	0.001	0.000	0.000
**58**	NEG	361.1062	1.47	[2M+H]-	Tyrosine	C00082	Tyrosine metabolism (map00350)	Level 1	-0.22	-0.03	0.25	0.001	0.000	0.000
**59**	NEG	391.2856	7.99	[M-H]-	Ursodeoxycholic acid	C07880	Secondary bile acid biosynthesis (map00121)	Level 1	0.13	0.02	-0.15	0.820	0.414	0.780
**60**	NEG	437.2910	7.99	[M+FA]-	Ursodeoxycholic acid	C07880	Secondary bile acid biosynthesis (map00121)	Level 1	0.49	0.07	-0.56	0.991	0.560	0.480
**61**	NEG	453.2857	7.45	[M+FA]-	Hyocholic acid	n/a	Bile acid metabolism	Level 1	0.82	0.11	-0.93	0.257	0.982	0.342
							**URINE**							
**1**	POS	114.0649	0.74	[M+H]+	Creatinine	C00791	Arginine and proline metabolism (map00330)	Level 1	-0.59	-0.11	0.70	0.006	0.304	0.000
**2**	POS	118.0849	0.72	[M+H]+	Betaine	C00719	Glycine, serine and threonine metabolism (map00260)	Level 1	0.21	-0.38	0.17	0.275	0.716	0.656
**3**	POS	118.1213	0.77	[M+H]+	2-Diethylaminoethanol	n/a		Level 2	0.91	0.17	-1.08	0.075	0.027	0.000
**4**	POS	132.0752	0.74	[M+H]+	Creatine	C00300	Glycine, serine and threonine metabolism (map00260)	Level 1	0.01	-0.02	0.01	0.782	0.943	0.541
**5**	POS	132.1001	0.79	[M+H]+	N,N-Diethylglycine	n/a		Level 2	-0.01	-0.04	0.04	0.964	0.008	0.014
**6**	POS	134.1161	0.87	[M+H]+	Bis (2-hydroxypropyl) amine	n/a		Level 2	0.76	0.10	-0.87	0.457	0.111	0.003
**7**	POS	137.0443	0.94	[M+H]+	Hypoxanthine	C00262	Purine metabolism (map00230)	Level 1	-0.10	-0.02	0.12	0.959	0.205	0.311
**8**	POS	172.0950	3.76	[M+H]+	N-butanoyl-lhomoserine lactone	n/a		Level 2	-0.02	0.04	-0.02	0.702	0.400	0.077
**9**	POS	180.0635	3.54	[M+H]+	Hippuric acid	C01586	Phenylalanine metabolism (map00360)	Level 1	0.83	0.16	-0.98	0.242	0.001	0.000
**10**	POS	194.0791	3.90	[M+H]+	Phenylacetylglycine	C05598	Phenylalanine metabolism (map00360)	Level 1	-0.14	0.27	-0.12	0.267	0.480	0.869

**11**	POS	195.0745	1.65	[M+H]+	Aminohippuric acid	n/a		Level 2	0.23	0.04	-0.27	0.070	0.009	0.000
**12**	POS	206.0789	4.81	[M+H]+	Cinnamoylglycine	n/a		Level 1	0.01	0.00	-0.02	0.104	0.021	0.000
**13**	POS	229.1523	0.95	[M+H]+	Isoleucylproline / Leucylproline	n/a		Level 2	-0.02	0.00	0.03	0.919	0.145	0.052
**14**	POS	229.1524	1.39	[M+H]+	Leucylproline / Isoleucylproline	n/a		Level 2	0.13	-0.24	0.11	0.962	0.250	0.367
**15**	POS	237.0845	2.95	[M+H]+	Formylkynurenine	C02700	Tryptophan metabolism (map00380)	Level 2	0.02	0.00	-0.02	0.984	0.073	0.041
**16**	POS	253.0979	3.59	Unknown	Unidentified (1)	n/a		Level 4	-0.35	0.64	-0.29	0.609	0.046	0.316
**17**	POS	253.1521	3.20	Unknown	Unidentified (2)	n/a		Level 4	0.16	0.03	-0.19	0.984	0.023	0.011
**18**	POS	259.0963	2.43	[M+H]+	Yangonin	C09980	Phytochemical compounds	Level 2	-0.15	-0.03	0.18	0.627	0.945	0.780
**19**	POS	283.1722	2.92	[M+H]+	Hexaethylene glycol	n/a	Polyethylene glycols	Level 2	0.06	-0.10	0.05	0.975	0.709	0.550
**20**	POS	291.1384	2.42	Unknown	Unidentified (3)	n/a		Level 4	-0.07	0.14	-0.06	0.206	0.010	0.432
**21**	POS	327.1975	3.15	[M+H]+	Heptaethylene glycol	n/a	Polyethylene glycols	Level 2	0.02	-0.03	0.01	0.931	0.681	0.428
**22**	POS	343.2920	7.07	Unknown	Unidentified (4)	n/a		Level 4	-1.54	2.84	-1.30	0.268	0.000	0.043
**23**	POS	344.2241	3.15	[M+NH4]+	Heptaethylene glycol	n/a	Polyethylene glycols	Level 2	0.12	-0.22	0.10	0.984	0.685	0.556
**24**	POS	371.3233	8.12	Unknown	Unidentified (5)	n/a		Level 4	-1.83	3.36	-1.54	0.073	0.004	0.568
**25**	POS	388.2501	3.35	[M+H]+	Octaethylene glycol	n/a	Polyethylene glycols	Level 2	0.19	-0.35	0.16	0.948	0.748	0.525
**26**	POS	399.2088	1.94	Unknown	Unidentified (6)	n/a		Level 4	-0.33	-0.22	0.55	0.603	0.994	0.621
**27**	POS	399.3547	9.22	Unknown	Unidentified (7)	n/a		Level 4	-1.51	2.79	-1.27	0.102	0.016	0.780
**28**	POS	413.1334	2.29	[M+H]+	Glucuronated unidentified (8) (m/z 237.1060)	n/a		Level 4	-0.74	1.36	-0.62	0.700	0.142	0.522
**29**	POS	413.1335	2.63	[M+H]+	Glucuronated unidentified (9) (m/z 237.1060)	n/a		Level 4	-0.44	0.81	-0.37	0.675	0.074	0.363
**30**	POS	418.2605	3.15	[M+NH4]+	Unidentified (10) (m/z 401.2397)	n/a		Level 4	-0.01	0.02	-0.01	0.587	0.005	0.063
**31**	POS	443.1439	2.57	[M+H]+	Glucuronated unidentified (11)	n/a		Level 4	-0.34	0.63	-0.29	0.644	0.164	0.630
**32**	POS	476.3019	3.67	Unknown	Unidentified (12)	n/a		Level 4	0.07	-0.12	0.06	0.830	0.966	0.649
**33**	POS	479.1525	4.09	[M+H]+	4'-O-methyl-(-)-epicatechin-5-O-beta-glucuronide	n/a		Level 2	-0.02	0.04	-0.02	0.723	0.497	0.942
**34**	POS	504.2545	3.71	[M+H]+	Glucuronated unidentified (13)	n/a		Level 4	-0.22	0.40	-0.18	0.727	0.311	0.773
**35**	POS	685.5773	7.06	[2M+H]+	Unidentified (14) (m/z 343.2962)	n/a		Level 4	-0.46	0.84	-0.38	0.354	0.281	0.997
**36**	NEG	158.0823	3.04	[M-H]-	5-Acetamidopentanoate	C03087	Lysine degradation (map00310)	Level 2	0.20	0.03	-0.23	0.547	0.227	0.016
**37**	NEG	173.0819	4.18	[M-H]-	Suberic acid	C08278	Fatty acids and conjugates metabolism	Level 2	0.07	0.01	-0.08	0.866	0.203	0.059
**38**	NEG	178.0509	3.54	[M-H]-	Hippuric acid	C01586	Phenylalanine metabolism (map00360)	Level 1	1.49	0.20	-1.69	0.532	0.019	0.000
**39**	NEG	191.0196	0.84	[M-H]-	Isocitrate	C00311	Citrate cycle (TCA cycle) (map00020)	Level 1	0.02	0.00	-0.03	0.902	0.299	0.118

**40**	NEG	191.0196	0.96	[M-H]-	Citrate	C00158	Citrate cycle (TCA cycle) (map00020)	Level 1	0.01	0.00	-0.01	0.964	0.267	0.149
**41**	NEG	193.0618	1.65	[M-H]-	Aminohippuric acid	n/a		Level 2	0.90	0.12	-1.02	0.537	0.020	0.000
**42**	NEG	194.0459	3.74	[M-H]-	Salicyluric acid (Alpha-Hydroxyhippuric acid)/ Dopaquinone	C00822	Tyrosine metabolism (map00350) or C07588 (salicyluric acid)	Level 2	0.98	0.13	-1.11	0.102	0.000	0.000
**43**	NEG	199.0975	5.20	[M-H]-	cis-4-Decenedioic acid	n/a		Level 2	0.03	0.00	-0.04	0.910	0.238	0.438
**44**	NEG	204.0666	4.81	[M-H]-	Cinnamoylglycine	n/a		Level 1	0.82	0.11	-0.93	0.155	0.016	0.000
**45**	NEG	212.0023	3.58	[M-H]-	Indoxylsulfuric acid	n/a		Level 1	-1.08	-0.14	1.22	0.829	0.021	0.003
**46**	NEG	216.9812	2.65	[M-H]-	5-Sulfosalicylic acid / 3-hydroxybenzoic acid-3-O-sulphate	n/a		Level 2	0.05	0.01	-0.06	0.012	0.039	0.000
**47**	NEG	230.9968	4.11	[M-H]-	Vanillin 4-sulfate / p-Hydroxyphenylacetic acid sulphate / 2,4-Dihydroxyacetophenone 5-sulfate	C00755	Phenylalanine metabolism (map00360) or C00755	Level 2	0.37	0.05	-0.42	0.461	0.138	0.005
**48**	NEG	235.0724	2.96	[M-H]-	Formylkynurenine	C02700	Tryptophan metabolism (map00380)	Level 2	0.04	0.00	-0.04	0.992	0.095	0.111
**49**	NEG	255.0510	2.48	[M-H]-	Piscidic acid	n/a		Level 2	-0.43	-0.06	0.48	0.000	0.075	0.000
**50**	NEG	313.0565	2.60	[M-H]-	1-Salicylate glucuronide / Beta-D-Glucopyranuronic acid	n/a		Level 2	0.63	0.08	-0.71	0.003	0.000	0.000
**51**	NEG	327.0721	3.54	[M-H]-	Glucuronide - Vanillin/4-Hydroxy-3-methylbenzoic acid/Anisic acid	C00755	Phenylalanine metabolism (map00360) or C00755	Level 2	0.47	0.06	-0.54	0.269	0.004	0.000
**52**	NEG	345.0978	5.52	[M-H]-	Glucuronide Unidentified (m/z 169.0659)	n/a		Level 4	-0.05	-0.01	0.05	0.000	0.700	0.000
**53**	NEG	354.0829	2.80	[M-H]-	Hippuric acid glucuronide	C01586	Phenylalanine metabolism (map00360)	Level 2	0.19	0.03	-0.22	0.013	0.001	0.000
**54**	NEG	627.1196	2.60	[2M+H]-	1-Salicylate glucuronide / Beta-D-Glucopyranuronic acid	n/a		Level 2	0.35	0.05	-0.39	0.002	0.000	0.000
							**FECES**							
**1**	POS	100.0759	2.07	[M+H]+	δ-Valerolactam	n/a		Level 1	0.27	-0.49	0.22	0.302	0.707	0.064
**2**	POS	130.0865	0.8	[M+H]+	Pipecolic acid	C00408	Lysine degradation (map00310)	Level 2	0.14	-0.08	-0.07	0.035	0.847	0.008
**3**	POS	132.102	0.91	[M+H]+	N,N-Diethylglycine / beta-Alaninebetaine	n/a		Level 2	-0.06	0.12	-0.05	0.572	0.441	0.975
**4**	POS	166.0867	2.15	[M+H]+	Phenylalanine	C00079	Phenylalanine metabolism (map00360)	Level 1	-0.02	0.04	-0.02	0.938	0.437	0.648
**5**	POS	205.0977	3.11	[M+H]+	Tryptophan	C00078	Tryptophan metabolism (map00380)	Level 1	-0.06	0.10	-0.05	0.617	0.290	0.834
**6**	POS	256.2645	15.92	[M+H]+	Palmitic amide	n/a	Fatty acids and conjugates metabolism	Level 2	-0.30	0.51	-0.21	0.000	0.817	0.000
**7**	POS	276.1966	11.61	Unknown	Unidentified (1)	n/a		Level 4	-0.08	-0.01	0.09	0.000	0.000	0.000
**8**	POS	280.1394	0.82	[M+H]+	N-(1-Deoxy-1-fructosyl)valine	n/a		Level 2	-0.01	0.02	-0.01	0.959	0.562	0.734
**9**	POS	282.2801	16.28	[M+H]+	Fatty acid (1) (Oleamide / Elaidamide)	n/a	Fatty acids and conjugates metabolism	Level 3	-0.41	0.49	-0.08	0.000	0.917	0.000

**10**	POS	283.2639	14.41	[M+H]+	Fatty acid (2) (Oleic Acid, Elaidic Acid, Vaccenic acid (RT14.41))	n/a	Fatty acids and conjugates metabolism	Level 3	-0.11	0.08	0.02	0.228	0.666	0.036
**11**	POS	284.2957	17.64	[M+H]+	Stearamide	C13846	Fatty acids and conjugates metabolism	Level 2	-0.10	0.19	-0.09	0.001	0.650	0.009
**12**	POS	293.212	12.1	[M+H]+	Oxylipins (1) (9-OxoOTrE)	n/a	PUFA derived oxylipins	Level 3	-0.08	-0.02	0.10	0.617	0.293	0.043
**13**	POS	294.1546	0.89	[M+H]+	N-(1-Deoxy-1-fructosyl)isoleucine/ N-(1-Deoxy-1-fructosyl)leucine	n/a		Level 2	-0.16	0.29	-0.13	0.978	0.596	0.473
**14**	POS	295.2277	13.42	[M+H]+	Oxylipin (2) (13-OxoODE, 9-OxoODE, 9(S)-HOTrE)	n/a	PUFA derived oxylipins	Level 3	-0.41	0.12	0.29	0.151	0.819	0.039
**15**	POS	297.2433	14.17	[M+H]+	Oxylipin (3) (9(R)-HODE)	n/a	PUFA derived oxylipins	Level 3	-0.20	0.37	-0.17	0.626	0.872	0.906
**16**	POS	299.2589	14.88	[M+H]+	Oxylipin (4) (HOME, 13-oxo-octadecanoic acid, 12-oxo-octadecanoic acid)	n/a	PUFA derived oxylipins	Level 3	-0.03	0.00	0.03	0.377	0.241	0.011
**17**	POS	319.2253	13.97	[M+H]+	Oxylipin (5) (HEPE, Oxo-ETE, EpETE)	n/a	PUFA derived oxylipins	Level 3	-0.27	0.16	0.11	0.071	0.974	0.042
**18**	POS	321.1812	6.58	Unknown	Unidentified (2)	n/a		Level 4	-0.12	-0.02	0.14	0.021	0.001	0.000
**19**	POS	391.2851	10.1	[M+H]+	Bile acid (1) (12-Ketodeoxycholic acid / Nutriacholic acid)	n/a	Bile acid metabolism	Level 3	-0.08	0.15	-0.07	0.604	0.958	0.434
**20**	POS	393.3374	16.23	[M+NH4]+	Docosatetraenoyl Ethanolamide (m/z 375.3264)	n/a		Level 2	-0.07	-0.01	0.08	0.350	0.556	0.045
**21**	POS	561.4891	13.67	Unknown	Unidentified (3)	n/a		Level 4	0.00	0.00	0.00	0.593	0.787	0.946
**22**	POS	595.3495	6.75	[M+H]+	Urobilin	C05793	Porphyrin and chlorophyll metabolism (map00860)	Level 2	-0.43	-1.17	1.60	0.362	0.004	0.000
**23**	POS	611.3444	6.31	[M+NH4]+	Urobilinogen	C05790	Porphyrin and chlorophyll metabolism (map00860)	Level 2	-0.11	-0.12	0.22	0.442	0.000	0.000
**24**	NEG	181.0511	3.82	[M-H]-	3,4-Dihydroxyhydrocinnamic acid	C10447	Tyrosine metabolism (map00350)	Level 1	-0.01	0.00	0.01	0.700	0.692	1.000
**25**	NEG	297.2439	13.38	[M-H]-	Ricinoleic acid	n/a	Fatty acids and conjugates metabolism	Level 2	0.99	0.13	-1.12	0.449	0.001	0.000
**26**	NEG	299.2595	14.36	[M-H]-	(R)-10-hydroxystearic acid	n/a	Fatty acids and conjugates metabolism	Level 2	0.11	0.01	-0.13	0.989	0.721	0.633
**27**	NEG	315.2543	11.93	[M-H]-	11,12-DHSA (11,12-dihydroxystearic acid) / isomer	n/a	Fatty acids and conjugates metabolism	Level 2	0.02	0.00	-0.02	0.193	0.040	0.000
**28**	NEG	319.1666	6.54	[M-H]-	Unidentified (1)	n/a		Level 4	-0.27	-0.04	0.30	0.055	0.001	0.000
**29**	NEG	345.2285	7.85	[M-H]-	9,10-Dihydroxyoctadecanedioic acid	n/a	Fatty acids and conjugates metabolism	Level 2	0.02	0.00	-0.02	0.698	0.966	0.540
**30**	NEG	385.1442	5.75	[M-H]-	Unidentified (2)	n/a		Level 4	-0.04	-0.01	0.05	0.012	0.001	0.000
**31**	NEG	389.2699	10.35	[M-H]-	Bile acid (1) (Nutriacholic acid / 12-Ketodeoxycholic acid)	n/a	Bile acid metabolism	Level 3	0.02	0.00	-0.02	0.963	0.859	0.712
**32**	NEG	391.2856	9.92	[M-H]-	Ursodeoxycholic acid	C07880	Secondary bile acid biosynthesis (map00121)	Level 1	-0.32	-0.04	0.36	0.165	0.983	0.116
**33**	NEG	435.2753	10.31	[M+FA]-	Bile acid (2) (Nutriacholic acid / 12-Ketodeoxycholic acid)	n/a	Bile acid metabolism	Level 3	0.38	0.05	-0.43	0.980	0.960	0.888
**34**	NEG	437.291	9.92	[M+FA]-	Ursodeoxycholic acid	C07880	Secondary bile acid biosynthesis (map00121)	Level 1	-0.12	-0.02	0.13	0.185	0.997	0.211

**35**	NEG	593.3347	6.7	[M-H]-	Urobilin	C05793	Porphyrin and chlorophyll metabolism (map00860)	Level 2	-0.17	-0.02	0.19	0.525	0.001	0.000

1 Ionization mode

2 MS M/Z, mass spectrometry mass-to-charge ratio

3 RT, retention time

4Abbreviations: FA, formic acid; OxoOTre, oxo-octadecatrienoic acid; OxoODE, oxo-octadecadienoic acid; HOTrE, hydroxy-octadecatrienoic acid; HODE, hydroxyoctadecadienoic acid; HOME, Hydroxy-octadecenoic acid; HEPE, hydroxy-eicosapentaenoic acid; Oxo-ETE, oxo-eicosatetraenoic acid; EpETE, epoxy-eicosatetraenoic acid.

5 KEGG, Kyoto Encyclopedia of Genes and Genomes; n/a, not available

6 RC Week 4/RC week 12/RC week 20, regression coefficient for the weeks 4, 12, and 20 from the sMBPLSR models

Table S5. sMBPLSR metabolites discriminating between minipigs fed HR and LR diets in the fecal metabolome

LIST NR	IM[1]	MS M/Z[2]	RT[3]	ION	METABOLITE[4]	KEGG[5]	PATHWAY	ID LEVEL	RC[6] HR	RC[6] LR	P-VAL ANOVA
**1**	POS	137.0457	0.89	[M+H]+	Hypoxanthine	C00262	Purine metabolism (map00230)	Level 1	0.00	0.00	0.000
**2**	POS	144.0811	3.56	[M+H]+	6-Methylquinoline / quinaldine / 2-Naphthylamine	n/a		Level 2	-0.05	0.05	0.000
**3**	POS	148.1334	0.72	Unknown	Unidentified (1)	n/a		Level 4	0.23	-0.23	0.000
**4**	POS	160.1335	0.79	[M+H]+	Methacholine / Propionylcholine	n/a		Level 2	-0.04	0.04	0.000
**5**	POS	250.1653	2.09	[M+NH4]+	Isobutyryl-L-carnitine/Butyryl-L-carnitine (m/z 232.1548)	n/a	Fatty acids and conjugates metabolism	Level 2	0.03	-0.03	0.000
**6**	POS	250.1654	1.70	[M+NH4]+	Isobutyryl-L-carnitine/Butyryl-L-carnitine (m/z 232.1548)	n/a	Fatty acids and conjugates metabolism	Level 2	0.01	-0.01	0.000
**7**	POS	271.1659	3.50	Unknown	Unidentified (2)	n/a		Level 4	0.07	-0.07	0.000
**8**	POS	271.1660	3.28	Unknown	Unidentified (3)	n/a		Level 4	0.19	-0.19	0.000
**9**	POS	283.2639	14.41	[M+H]+	Fatty acid (1) (Oleic Acid, Elaidic Acid, Vaccenic acid (RT14.41))	n/a	Fatty acids and conjugates metabolism	Level 3	0.10	-0.10	0.001
**10**	POS	294.1546	0.89	[M+H]+	N-(1-Deoxy-1-fructosyl)isoleucine/ N-(1-Deoxy-1-fructosyl)leucine	n/a		Level 2	-0.01	0.01	0.000
**11**	POS	297.2433	14.17	[M+H]+	Oxylipin (1) (HODE)	n/a	PUFA derived oxylipins	Level 3	0.00	0.00	0.101
**12**	POS	299.2589	14.88	[M+H]+	Oxylipin (2) (HOME, 13-oxo-octadecanoic acid, 12-oxo-octadecanoic acid)	n/a	PUFA derived oxylipins	Level 3	-0.23	0.23	0.120
**13**	POS	311.2220	7.88	[M+H]+	Oxylipin (3) (13S-HpOTrE(gamma)/13(S)-HpOTrE)	n/a	PUFA derived oxylipins	Level 3	-0.01	0.01	0.000
**14**	POS	315.1963	12.44	[M+H]+	Lys Pro Ala	n/a		Level 2	0.08	-0.08	0.000
**15**	POS	319.2253	13.97	[M+H]+	Oxylipin (4) (HEPE, Oxo-ETE, EpETE)	n/a	PUFA derived oxylipins	Level 3	0.35	-0.35	0.000
**16**	POS	324.2906	14.36	[M+H]+	Linoleoyl Ethanolamide	n/a	Fatty acids and conjugates metabolism	Level 2	0.03	-0.03	0.200
**17**	POS	350.3427	17.80	Unknown	Unidentified (4)	n/a		Level 4	0.00	0.00	0.218
**18**	POS	357.2795	9.71	[M+H]+	Docosahexaenoic Acid ethyl ester / THA	n/a	Fatty acids and conjugates metabolism	Level 3	-0.06	0.06	0.574
**19**	POS	373.2747	13.21	[M+H]+	Bile acid (1) (3-OXO-CHOL-11-ENIC ACID)	n/a	Bile acid metabolism	Level 3	0.12	-0.12	0.132
**20**	POS	379.2853	14.19	[M+H]+	Norchenodeoxycholic acid	n/a	Bile acid metabolism	Level 2	0.04	-0.04	0.000
**21**	POS	379.2962	8.38	[M+NH4]	N-arachidonoyl glycine (NAGly)	n/a	Fatty acids and conjugates metabolism	Level 2	0.00	0.00	0.000
**22**	POS	389.2695	11.15	[M+H]+	Bile acid (2) (6β-Hydroxy-3-oxochol-4-en-24-oic Acid)	n/a	Bile acid metabolism	Level 3	0.05	-0.05	0.058
**23**	POS	393.3374	16.23	[M+NH4]	Docosatetraenoyl Ethanolamide (m/z 375.3264)	n/a	Fatty acids and conjugates metabolism	Level 2	0.15	-0.15	0.000

**24**	POS	407.3167	15.58	[M+H]+	Fatty acids (2) (MG(22:4/0:0/0:0))	n/a	Fatty acids and conjugates metabolism	Level 3	0.05	-0.05	0.030
**25**	POS	411.3269	17.92	[M+H]+	γ-Tocotrienol	C14155	Ubiquinone and other terpenoid-quinone biosynthesis (map00130)	Level 2	0.00	0.00	0.000
**26**	POS	595.3495	6.75	[M+H]+	L-Urobilin	C05793	Porphyrin and chlorophyll metabolism (map00860)	Level 2	0.33	-0.33	0.004
**27**	POS	601.5415	14.41	[2M+H]+	Fatty acid (3) (Oleic Acid, Elaidic Acid, Vaccenic acid (RT14.41))	n/a	Fatty acids and conjugates metabolism	Level 3	0.01	-0.01	0.001
**28**	NEG	89.0244	0.89	[M-H]-	Lactic acid	C00256	Pyruvate metabolism (map00620)	Level 2	-0.24	0.24	0.000
**29**	NEG	117.0194	1.20	[M-H]-	Succinic acid	C00042	Citrate cycle (TCA cycle) (map00020)	Level 1	-0.44	0.44	0.000
**30**	NEG	117.0559	2.82	[M-H]-	2-hydroxyisovaleric acid (2-Hydroxy-3-methylbutyric acid)	n/a		Level 1	-0.17	0.17	0.000
**31**	NEG	129.0560	3.42	[M-H]-	3-Methyl-2-oxovaleric acid / 2-keto-n-caproic acid	n/a		Level 3	0.02	-0.02	0.280
**32**	NEG	129.0560	3.84	[M-H]-	Ketoleucine	C00233	Valine, leucine and isoleucine degradation (map00280)	Level 1	0.05	-0.05	0.373
**33**	NEG	131.0716	4.29	[M-H]-	2-Hydroxyisocaproic acid	n/a	Leucine metabolism	Level 1	-0.61	0.61	0.000
**34**	NEG	147.0300	0.89	[M-H]-	2-Hydroxy-glutarate	C03196	Lysine degradation (map00310)	Level 1	-0.40	0.40	0.000
**35**	NEG	159.0666	4.63	[M-H]-	3,3-Dimethylglutaric acid / 3-Methyladipic acid	n/a		Level 2	-1.10	1.10	0.000
**36**	NEG	159.0666	3.86	[M-H]-	Pimelic acid	C02656	Biotin metabolism (map00780)	Level 1	0.24	-0.24	0.000
**37**	NEG	165.0560	5.03	[M-H]-	D-(+)-3-Phenyllactic acid	C05607	Phenylalanine metabolism (map00360)	Level 1	-1.09	1.09	0.000
**38**	NEG	165.0560	5.63	[M-H]-	Desaminotyrosine / 4-Hydroxyphenyl-2-propionic acid	n/a		Level 2	-0.04	0.04	0.000
**39**	NEG	173.0822	5.96	[M-H]-	2,4-Dimethyladipic acid / Ethyladipic acid	n/a		Level 2	-0.04	0.04	0.000
**40**	NEG	173.0822	4.92	[M-H]-	Suberic acid	C08278	Fatty acids and conjugates metabolism	Level 2	0.05	-0.05	0.029
**41**	NEG	181.0510	3.13	[M-H]-	Hydroxyphenyllactic acid	C03672		Level 1	-0.25	0.25	0.000
**42**	NEG	187.0978	5.91	[M-H]-	Nonanedioic acid	n/a		Level 2	0.08	-0.08	0.926
**43**	NEG	188.0566	1.08	[M-H]-	N-Acetyl-L-glutamic acid	C00624	Arginine biosynthesis (map00220)	Level 2	-0.09	0.09	0.000
**44**	NEG	201.1135	6.86	[M-H]-	Sebacic acid	C08277	Fatty acids and conjugates metabolism	Level 1	0.08	-0.08	0.004
**45**	NEG	203.0564	2.75	[M-H]-	L-Ascorbic acid ethyl ester	n/a		Level 2	-0.16	0.16	0.000
**46**	NEG	215.1292	7.76	[M-H]-	Undecanedioic acid	n/a	Fatty acids and conjugates metabolism	Level 2	0.14	-0.14	0.000
**47**	NEG	218.1037	2.32	[M-H]-	Pantothenic acid	C00864	Pantothenate and CoA biosynthesis (map00770)	Level 1	-0.19	0.19	0.000
**48**	NEG	229.1448	8.63	[M-H]-	Dodecanedioic acid	n/a	Fatty acids and conjugates metabolism	Level 2	0.14	-0.14	0.006
**49**	NEG	231.9925	3.20	Unknown	Unidentified (1)	n/a		Level 4	0.09	-0.09	0.005

**50**	NEG	241.1084	6.87	Unknown	Unidentified (2)	n/a		Level 4	-0.01	0.01	0.152
**51**	NEG	241.1085	4.85	Unknown	Unidentified (3)	n/a		Level 4	0.16	-0.16	0.000
**52**	NEG	243.1240	6.44	[M-H]-	Fatty acid (1) (4-Oxododecanedioic acid)	n/a	Fatty acids and conjugates metabolism	Level 3	-0.03	0.03	0.000
**53**	NEG	243.1241	5.92	[M-H]-	Fatty acid (2) (4-Oxododecanedioic acid)	n/a	Fatty acids and conjugates metabolism	Level 3	-0.06	0.06	0.000
**54**	NEG	295.2283	12.82	[M-H]-	Oxylipin (1) (13-HODE)	n/a	PUFA derived oxylipins	Level 3	-0.04	0.04	0.002
**55**	NEG	297.1135	8.14	[M-H]-	(±)-Enterolactone	n/a		Level 1	0.88	-0.88	0.000
**56**	NEG	297.2438	14.82	[M-H]-	Fatty acid (3) (12-oxo-octadecanoic acid / 6R,7S-Epoxy-octadecanoate)	n/a	Fatty acids and conjugates metabolism	Level 3	-0.07	0.07	0.097
**57**	NEG	297.2439	13.38	[M-H]-	Ricinoleic acid	n/a	Fatty acids and conjugates metabolism	Level 2	-0.13	0.13	0.224
**58**	NEG	299.2595	14.36	[M-H]-	(R)-10-hydroxystearic acid	n/a	Fatty acids and conjugates metabolism	Level 2	0.21	-0.21	0.001
**59**	NEG	307.1919	11.79	[M-H]-	Corchorifatty acid A	n/a	Fatty acids and conjugates metabolism	Level 2	0.54	-0.54	0.000
**60**	NEG	311.2230	12.60	[M-H]-	Oxylipin (2) ((±)13-HpODE / (±)9-HpODE)	n/a	PUFA derived oxylipins	Level 3	-0.42	0.42	0.000
**61**	NEG	313.2388	13.49	[M-H]-	Oxylipin (3) (9,10-DHOME / 12,13-DHOME)	n/a	PUFA derived oxylipins	Level 3	0.02	-0.02	0.111
**62**	NEG	315.1242	5.78	[M-H]-	Verimol B	n/a		Level 2	0.03	-0.03	0.000
**63**	NEG	315.2538	9.97	[M-H]-	Fatty acid (4) (9,10-Dihydroxystearic acid)	n/a	Fatty acids and conjugates metabolism	Level 3	-0.06	0.06	0.000
**64**	NEG	315.2541	10.50	[M-H]-	Fatty acid (5) (12,13-dihydroxy stearic acid)	n/a	Fatty acids and conjugates metabolism	Level 3	-0.18	0.18	0.033
**65**	NEG	315.2544	11.60	[M-H]-	Fatty acid (6) (11,12-dihydroxy stearic acid)	n/a	Fatty acids and conjugates metabolism	Level 3	0.05	-0.05	0.000
**66**	NEG	321.2074	11.24	Unknown	Unidentified (4) (possible 7,10,13,16,19-Docosapentaynoic acid)	n/a		Level 4	0.06	-0.06	0.001
**67**	NEG	327.0879	5.66	Unknown	Unidentified (5) (possible isoflavone)	n/a		Level 4	0.30	-0.30	0.000
**68**	NEG	327.2179	10.17	[M-H]-	Corchorifatty acid F	n/a	Fatty acids and conjugates metabolism	Level 2	-0.21	0.21	0.000
**69**	NEG	327.2180	8.96	[M-H]-	Prostaglandin (2,3-Dinor-8-iso prostaglandin F1alpha)	n/a		Level 3	-0.15	0.15	0.000
**70**	NEG	329.1034	6.06	[M-H]-	Isoflavone (1) (Samaderin A)	n/a		Level 3	0.87	-0.87	0.000
**71**	NEG	329.2336	9.71	[M-H]-	Oxylipin (4) (9,10,13-TriHOME/11,12,13-TriHOME/5,8,12-TriHOME)	n/a	PUFA derived oxylipins	Level 3	-0.63	0.63	0.000
**72**	NEG	329.2337	8.14	[M-H]-	Oxylipin (5) (9,10,13-TriHOME/11,12,13-TriHOME/5,8,12-TriHOME)	n/a	PUFA derived oxylipins	Level 3	-0.71	0.71	0.000
**73**	NEG	333.0902	8.14	[M+Cl]-	Enterolactone	n/a		Level 2	0.01	-0.01	0.000
**74**	NEG	345.2285	7.85	[M-H]-	9,10-Dihydroxyoctadecanedioic acid	n/a	Fatty acids and conjugates metabolism	Level 2	-1.06	1.06	0.000
**75**	NEG	349.2388	12.69	[M-H]-	Tetrahydrocorticosterone	n/a		Level 2	0.06	-0.06	0.000

**76**	NEG	387.2544	11.10	[M-H]-	Bile acid (1) (7alpha-hydroxy-3-oxochol-4-en-24-oic Acid)	n/a	Bile acid metabolism	Level 3	0.65	-0.65	0.045
**77**	NEG	391.2856	9.92	[M-H]-	Ursodeoxycholic acid	n/a		Level 1	-0.32	0.32	0.683
**78**	NEG	407.2805	8.35	[M-H]-	Bile acid (2) (Allocholic acid/Hyocholic acid/Muricholic acid/Ursocholic acid)	n/a	Bile acid metabolism	Level 3	-0.09	0.09	0.836
**79**	NEG	409.1905	7.19	Unknown	Unidentified (6)	n/a		Level 4	0.01	-0.01	0.000
**80**	NEG	413.1955	9.03	Unknown	Unidentified (7)	n/a		Level 4	-0.13	0.13	0.004
**81**	NEG	421.2963	14.03	[M+FA]-	Bile acid (3 (Allolithocholic acid / Lithocholic acid)	n/a	Bile acid metabolism	Level 3	-0.02	0.02	0.859
**82**	NEG	433.2598	11.10	[M+FA]-	Bile acid (4) (7alpha-hydroxy-3-oxochol-4-en-24-oic Acid)	n/a	Bile acid metabolism	Level 3	0.38	-0.38	0.049
**83**	NEG	437.2906	10.73	[M+FA]-	Bile acid (5) (m/z 391.2855; Murocholic acid / Deoxycholic acid / Chenodeoxycholic Acid)	n/a	Bile acid metabolism	Level 3	-0.06	0.06	0.797
**84**	NEG	437.2910	9.92	[M+FA]-	Ursodeoxycholic acid	n/a		Level 1	-0.25	0.25	0.888
**85**	NEG	479.3016	11.72	[M-H]-	LysoPE(0:0/18:2(9Z,12Z))	n/a	Fatty acids and conjugates metabolism	Level 2	-0.01	0.01	0.000
**86**	NEG	537.3285	13.75	[M-H]-	Fatty acid (7) (PG(20:1(11Z)/0:0))	n/a	Fatty acids and conjugates metabolism	Level 3	-0.05	0.05	0.000
**87**	NEG	659.2134	6.09	[2M-H]-	Isoflavone (2) (Samaderin A)	n/a		Level 3	0.00	0.00	0.000

1 Ionization mode

2 MS M/Z, mass spectrometry mass-to-charge ratio

3 RT, retention time

4Abbreviations: UI, Unidentified compound; FA, formic acid; HODE, hydroxyoctadecadienoic acid; HOME, Hydroxy-octadecenoic acid; HpHOTrE, hydroperoxy-octadecatrienoic acid; HEPE, hydroxy-eicosapentaenoic acid; Oxo-ETE, oxo-eicosatetraenoic acid; EpETE, epoxy-eicosatetraenoic acid; HpODE, Hydroperoxy-octadecadienoic acid; DiHOME, dihydroxy-octadecenoic acid; TriHOME, trihydroxy-octadecenoic acid; LysoPE, Lysophosphatidylethanolamine; PG, phosphoglycerol;

5 KEGG, Kyoto Encyclopedia of Genes and Genomes; n/a, not available

6 RC HR/LR, regression coefficient for the HR/LR group from the MBPLSR models

Table S6. Feed ingredients

	Diet	
	**LR**	**HR**
Ingredients, g/kg (as-fed basis)*		
Wheat starch	232.8	232.8
Whole grain wheat (milled)	150	150
Wheat bran (finely milled)	125	125
Wheat gluten	65	65
LT Fishmeal	20	20
HiMaize® 260 (60% resistant starch)	**200**	-
Fructose crystalline (food grade)	-	**200**
Lard 92/15	150	150
Monocalcium phosphate 17/22.7	36.7	36.7
Limestone (39 % Ca)	9.0	9.0
Salt (NaCl)	6.5	6.5
Lysine monohydrochloride	1.7	1.7
Choline chloride (70 %)	1.3	1.3
Vitamin premix **	2.0	2.0

LR, lower-risk diet; HR, high-risk diet

* Formulated to supply 55% energy from carbohydrates, 10% energy from protein and 35% energy from fat

** amount per kg additive: 1000 IE vitamin A, 1000 IE vitamin D3, 31500 mg alpha-tocopherol, 34615,4 mg DL-alphatocopherolacetate, 1050 mg vitamin B1, 1050 mg vitamin B2, 1575 mg vitamin B6, 10,5 mg vitamin B12, 5250 mg Ca-D-pantothenic acid, 10500 mg niacin, 26.25 mg biotin, 1050 mg vitamin K3, 42000 mg Fe as $FeSO_4$, 7500 mg Cu as $CuSO_4$, 21000 mg Mn as MnO.

Table S7. Compound-dependent LC-MS/MS parameters; declustering potential (DP), entrance potential (EP), collision energy (CE), and cell exit potential (CXP).

	Q1 mass	Q3 mass	DP	EP	CE	CXP
Acetic acid quantifier	193.8	151.9	-80	-15	-15	-15
Acetic acid qualifier	193.8	46.0	-80	-15	-50	-15
$^{13}C_2$ acetic acid quantifier	196.0	152.0	-100	-15	-24	-13
$^{13}C_2$ acetic acid qualifier	196.0	121.8	-100	-15	-24	-10
Propionic acid quantifier	208.0	136.9	-20	-10	-24	-9
Propionic acid qualifier	208.0	46.0	-20	-10	-50	-20
$^{13}C_1$ propionic acid quantifier	209.0	137.0	-20	-10	-25	-9
$^{13}C_1$ propionic acid qualifier	209.0	165.1	-20	-10	-18	-11
Isobutyric acid quantifier	221.9	178.9	-100	-2	-18	-15
Isobutyric acid qualifier	221.9	42.0	-60	-2	-70	-19
Butyric acid quantifier	221.9	178.9	-100	-2	-18	-15
Butyric acid qualifier	221.9	42.0	-60	-2	-70	-19
$^{13}C_2$ butyric acid quantifier	224.0	205.8	-100	-10	-20	-12
$^{13}C_2$ butyric acid qualifier	224.0	180.0	-100	-10	-18	-15
Succinic acid quantifier	387.1	234.1	-30	-10	-25	-12
$^{13}C_2$ succinic acid qualifier	387.1	97.8	-30	-10	-47	-10
$^{13}C_2$ succinic acid quantifier	389.1	99.9	-145	-10	-40	-10
Succinic acid qualifier	389.1	236.1	-145	-10	-24	-15
Isovaleric acid quantifier	235.9	137.0	-15	-15	-30	-15
Isovaleric acid qualifier	235.9	46.0	-15	-15	-40	-15
Valeric acid quantifier	235.9	137.0	-15	-15	-30	-15
Valeric acid qualifier	235.9	46.0	-15	-15	-40	-15
$^{13}C_3$ valeric acid quantifier	239.0	136.9	-120	-10	-27	-15
$^{13}C_3$ valeric acid qualifier	239.0	151.8	-120	-10	-24	-12
